# Synthesis and Surface Properties of Piperidinium-Based Herbicidal Ionic Liquids as a Potential Tool for Weed Control

**DOI:** 10.1021/acs.jafc.3c00356

**Published:** 2023-03-06

**Authors:** Marta Wojcieszak, Anna Syguda, Aneta Lewandowska, Agnieszka Marcinkowska, Katarzyna Siwińska-Ciesielczyk, Michalina Wilkowska, Maciej Kozak, Katarzyna Materna

**Affiliations:** † Faculty of Chemical Technology, Poznan University of Technology, Berdychowo 4, Poznan 60-965, Poland; ‡ Department of Biomedical Physics, Faculty of Physics, Adam Mickiewicz University in Poznań, Uniwersytetu Poznańskiego 2, Poznan 61-614, Poland

**Keywords:** herbicidal ionic liquids, static contact angle, sliding angle, dicamba, zeta potential

## Abstract

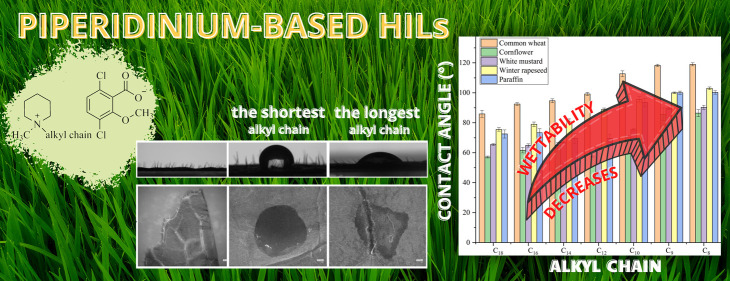

A series of piperidinium-based herbicidal ionic liquids (HILs) were synthesized and investigated. The designed HILs, obtained with high yields, consisted of cation 1-alkyl-1-methylpiperidinium with surface activity and a commercially available herbicidal anion: (3,6-dichloro-2-methoxy)benzoates (dicamba). The above-mentioned compounds were characterized in terms of surface activity and phytotoxicity. Preliminary results were obtained at higher wettability for all HILs when compared to the wettability of commercial Dicash, with HIL having 18 atoms in the carbon chain being the best effectiveness in wetting surfaces (weeds and crop leaves), whereby a drop of HILs with short alkyl chains (C_8_–C_10_) could not slide down a leaf. Our findings present that wettability or mobility of HILs drops varied depending on the plant species. Moreover, in this study, by zeta potential and atomic force microscopy measurements, we provide conclusive evidence to demonstrate that alkyl chain elongation plays a significant role in the evolution of surface properties of HILs.

## Introduction

Agriculture is a basic sector of the economy. Many problems hinder agricultural development, the most important of which is weed infestations in fields.^1^ This infestation is the result of intensive fertilization of plants, which increases crop yields but promotes the development of weeds. An effective method of weed control is the use of chemical preparations in the form of herbicides.^2^ An extremely important aspect of the use of herbicides in plant production is the possibility that weeds will become resistant to plant protection agents.^3−5^ One of the most common herbicides is dicamba, (3,6-dichloro-2-methoxy)benzoic acid, which was developed in the early 1960s as a selective herbicide for pre- and postemergence control of weeds in cereal crops.^6−9^ After application, dicamba can translocate through all broadleaf weeds, causing their destruction.^9^ Herbicides based on dicamba have high vapor pressures (2.6 × 10^–8^ atm at 25 °C),^10^ suggesting substantial volatility after application.^7,11,12^

In recent years, the efforts of various research groups have focused on the transformation of herbicides into herbicidal ionic liquids (HILs) for use in crop production.^13−19^ HILs are organic salts with herbicidal anions that exist in a molten state at temperatures below 100 °C.^15,16^ The main function of HILs is to protect crops by enhancing the activities of herbicides and reduce their environmental risk.^9,18,20,21^ Ionic liquids in which the anions exhibit herbicidal activity have been widely reported in the literature.^22−24^ An attractive solution for increasing the effectiveness of HILs is bifunctional herbicidal ionic liquids, which are a combination of an anion with herbicidal activity and a cation with surface properties.^25,26^ This combination produces an increase in the surface area covered by a herbicide and improved adhesion of the preparation to the surface of plant leaves. The aforementioned result was observed by our previous work where we analyzed morpholinium HILs and their wettability of the leaf surface.^21^ The influence of the length or nature of the alkyl chain on the surface activity of ionic liquids is well known.^27,28^ Studies have shown that the length of the carbon chain in a surfactant impacts the interfacial properties, which translates into a significant reduction in surface tension and excellent wetting ability.^29,30^ Continuing this thought, to contribute toward understanding the wetting phenomenon, the values of contact angle (CA) are established experimentally. The CA values are very useful for characterizing droplets deposited on a surface^31−33^ and are determined by interactions in the liquid (forming the droplets), which spreads not only on the plant surface under consideration but also on the area covered by the herbicide spray. The CA values are crucial for determining the type of interaction between a test surface and a wetting compound.^34,35^ Surface properties are an important influence factor for the effectiveness of HILs, because the physical and chemical attributes of HILs can result in different effects on a leaf surface. In the case of herbicides for which the efficacy depends on the quality of leaf coverage, low product retention can result in failure of weed control.^36^ The spraying process includes the formation of droplets, the retention and spread of these droplets over a plant, and the penetration of the active substance through relevant parts of the leaf structure until the target is effectively reached.^33−36^

The aim of this study was to focus on a series of novel piperidinium-based HILs that consist of amphiphilic cations and anions with potential herbicidal activity. The synthesis, thermal analysis, surface activity, phytotoxicity, atomic force microscopy (AFM) analysis, and zeta potential of the studied HILs are presented. The main purpose of designing compounds was to develop research on the surface properties of HILs, in particular, to expand the topic of wettability. To our knowledge, the wettability of biological surfaces by HILs has been described for the first time in our previous work.^21^ The aforementioned wettability tests were based on the studied static CA on biological systems. Seeing the potential of our research, we wanted to go one step further and focus on the issue of drop mobility of novel piperidinium-based HILs on the surfaces of weed leaves which to the best of our knowledge has not been reported thus far. Considering the problem of formulation may runoff from sprayed surfaces, it seems reasonable to study the mobility of new formulations of HILs from the weed leaves surface, which is crucial for effective crop protection.

## Materials and Methods

### Materials

1-Methylpiperidine (CAS 626-67-5) 98%, 1-bromooctane (CAS 111-83-1) 98%, 1-bromononane 98% (CAS 98-639-58), 1-bromodecane (CAS 112-29-8) 98%, 1-bromododecane (CAS 143-15-7) 97%, 1-bromotetradecane (CAS 112-71-0) 97%, 1-bromohexadecane (CAS 112-82-3) 98%, 1-bromooctadecane (CAS 112-89-0) 98%, reagents for two-phase system titration: [dimidium bromide (CAS 95-518-67-2) 95%, patent blue V sodium salt (CAS 20262-76-4) 97%, and sodium dodecylsulfate(VI) (CAS 151-21-3) 98%] were purchased from Sigma-Aldrich. (3,6-Dichloro-2-methoxy)benzoic acid (dicamba) (CAS 1918-00-9) 95% was purchased from Organika-Sarzyna (Poland). Acetone (CAS 67-64-1) 99%, ethyl acetate (CAS 141-78-6) 99%, chloroform (CAS 67-66-3) 98.5%, silver(I) nitrate(V) (CAS 7761-88-8) 99%, and sodium bicarbonate (CAS 144-55-8) 99% were purchased from Avantor.

### Synthesis of 1-Alkyl-1-methylpiperidinium Bromides

First, 0.05 mol of 1-methylpiperidine was dissolved in 5 cm^3^ of acetone and placed in a 250 cm^3^ round-bottomed flask, to which 0.0525 mol of the appropriate alkyl bromide and 10 cm^3^ of acetone were added. The reaction was carried out in acetone under reflux for 24 h. The flasks were then placed in a refrigerator for 24 h. The resulting products were vacuum-filtered and washed with a small quantity of cold ethyl acetate. The obtained compounds with C_8_H_17_ to C_10_H_21_ substituents were dried in a vacuum desiccator over P_2_O_5_, and the remaining products were dried in a vacuum oven at 60 °C for 24 h.

### Synthesis of 1-Alkyl-1-methylpiperidinium (3,6-Dichloro-2-methoxy)benzoates

A reagent mixture consisting of 0.01 mol of dicamba in acid form, 20 cm^3^ of distilled water, and 0.011 mol of a 10% aqueous solution of sodium bicarbonate was mixed in a round-bottomed flask equipped with a magnetic stirring bar, a reflux condenser, and an addition funnel. The mixture was heated at 50 °C until the solution became clear. Afterward, 0.01 mol of 1-alkyl-1-methylpiperidinium bromide dissolved in 20 cm^3^ of water was added to the solution, which was stirred for 30 min at room temperature. Then, the product was extracted from the aqueous phase with 50 cm^3^ of chloroform and washed with distilled water until bromide ions were no longer detected using AgNO_3_. The chloroform was removed, and the product was dried under reduced pressure at 60 °C for 24 h.

### NMR Analysis

Proton nuclear magnetic resonance (^1^H NMR) spectra were recorded using a Bruker Ascend 400 MHz NanoBay spectrometer operating at 400 MHz with tetramethylsilane as the internal standard CDCl_3_ as a solvent. Carbon-13 nuclear magnetic resonance (^13^C NMR) spectra were obtained with the same instrument at 100 MHz. All analyses were performed at Adam Mickiewicz University, Poznan (Poland).

### Thermal Analysis

Thermogravimetric analysis (TGA) was used to study the thermal stability of the ionic liquids. Measurements were made on a Tarsus TG 209 F3 analyzer (NETZSCH-Geratebau GmbH, Germany) in the temperature range of 30–600 °C. Approximately 10 mg of a sample was placed in a platinum crucible and heated at a rate of 10 °C/min under a nitrogen atmosphere (the flow rates of the protective and purge gases were 10 and 20 mL/min, respectively).

The thermal transition temperatures were determined by differential scanning calorimetry (DSC) using a DSC1 instrument (Mettler-Toledo, Greifensee, Switzerland). Before measurements were performed, 5–10 mg of a synthesized ionic liquid were placed in an aluminum pan and sealed with a pan lid. Then, the sample was cooled with an intracooler to −80 °C at a cooling rate of 10 °C/min and heated at the same rate under a nitrogen atmosphere.

### Surface Activity Studies

The surface properties (surface tension and CA) of the samples were measured using a DSA 100 analyzer (Krüss, Germany, accuracy ± 0.01 mN/m) at 25 °C. The sample temperature was monitored using a Fisherbrand FBH604 thermostatic bath (Fisher, Germany, accuracy ± 0.1 °C). The surface tension of the samples was determined based on the shape of an axisymmetric drop placed at the tip of a needle. An image of the drop was taken and digitized. The surface tension (γ in mN/m) was determined by using the Laplace equation to analyze the drop profile.

The parameters of the critical micelle concentration (CMC) and the surface tension at the CMC (γ_CMC_) were determined from the intersection of two straight lines drawn in the low- and high-concentration regions of the surface tension curves (γ vs log *C* curves) using linear regression analysis.

Surface excess concentrations at the saturated interface (Γ_max_), the minimum surface occupied by a molecule at the interface (*A*_min_), Gibbs free energy of the adsorption layer (Δ*G*^0^_ads_), CMC/C_20_ ratio, and the adsorption efficiency, pC_20_, have been presented in our previous reports.^22,37,38^

The static CA can be determined from the image of a drop on a test surface. Liquid drops are deposited on a solid hydrophobic surface. The actual drop shape and contact line are determined, and the drop shape is fitted to a mathematical model from which the CA is calculated.

In this study, the sliding angle (SA) was determined using the tilting plate method (using an angle of inclination of up to 90° with a resolution of 0.01°, an accuracy of 0.3° ± 0.1°, and a tilt speed range of 0.5°–50°/s). Initially, the drop was deposited on a test surface placed on a table. The table was then slowly tilted to increase the angle of inclination of the surface. At first, the drop did not move but deformed to an extent that depends on the liquid density, drop volume, and surface tension. At a particular angle of inclination, the drop started to move and slid or rolled across the surface. The entire process was digitalized.

The static CA and SA were performed on the adaxial and abaxial sides of leaves.

In this study, paraffin (a model surface used in laboratory) and biological systems, common wheat (*Triticum aestivum* L.), cornflower (*Centaurea cyanus* L.), winter rapeseed (*Brassica napus* L.), and white mustard (*Sinapis alba* L.), were analyzed as the solid phase.

### Zeta Potential Measurement

The zeta potential of the ionic liquid aggregates in aqueous solution was measured at 25 °C on a Zetasizer Nano-ZS (Malvern Instrument Ltd.) equipped with an autotitrator. The zeta potential was estimated using the Smoluchowski equation. The pH of the ionic liquid aggregates was automatically adjusted by an automatic titrator using hydrochloric acid (0.02 mol/L) or sodium hydroxide (0.02 mol/L). The test samples were solutions of HILs at CMC concentrations.

### Statistical Analysis

Statistical analysis was carried out using the standard error of the mean (SEM) value method; that is, the standard errors in the mean were estimated. The SEM was calculated using the equation given below:
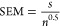
where SEM is the standard error of the mean, *s* is the sample standard deviation, and *n* is the number of samples.

### Observation of the Microstructure

A digital microscope (VHX-7000 series, Keyence) was used to observe the morphology of the leaf surfaces.

### Atomic Force Microscopy

The samples of the analyzed herbicidal ionic liquids were dissolved in water, and small volumes (3 μL) of the test solutions containing different concentrations of HIL were deposited on freshly prepared mica substrates and dried. Topographic images were collected using a NanoWizard IV (JPK, Germany) atomic force microscope and Tap150AL AFM cantilevers (Ted Pella, Inc., Redding, USA). The experimental AFM data obtained for the analyzed HILs were processed and analyzed using Gwyddion v2.58 image processing software.^39^

### Phytotoxicity

Phytotoxicity of synthesized 1-alkyl-1-methylpiperidinium (3,6-dichloro-2-methoxy)benzoates in relation to dicotyledonous plants was measured on the basis of measurements of shoot and root growth inhibition of the plant. Cornflower (*Centaurea cyanus* L.) was used as a model dicotyledonous plant. Commercial product—Dicash (480 g of dicamba in the form of dimethylammonium salt per 1 L) was used as the reference sample. The tests were carried out using vertical plastic Phytotoxkit containers (Tigret company, Belgium). The sand was previously screened and cleaned by washing it several times with tap water and deionized water and then dried in a dryer for 24 h at 105 °C. Each container was filled with 130 ± 0.1 g of sand. 0.25 mmols of the tested compound was placed in a 100 mL graduated flask. Consequently, the initial solutions of the tested compounds were prepared at a concentration of 2.5 mmol/L. Then a 10-fold dilution was made by taking 10 mL of the initial solution and diluting it in a 100 mL volumetric flask. In the next stage, a 10-fold dilution was made again. Finally, 0.025 mmol/L use solutions of the compounds were obtained. Afterward, 25 mL of the prepared solutions were taken and used to water the sand in a container, which corresponded to 0.0048 mmol of the tested compound per kg of dry sand. One of the containers was prepared as a “0” control, which contained only deionized water. Next, 10 seeds of tested plants were planted in each container and incubated for 7 days at 25 °C. Seven days after sowing, the lengths of the shoots and roots were measured. The tests were performed according to the PN-ISO 11269-1 (1998) standard.

## Results and Discussion

### Synthesis

**Scheme 1:**
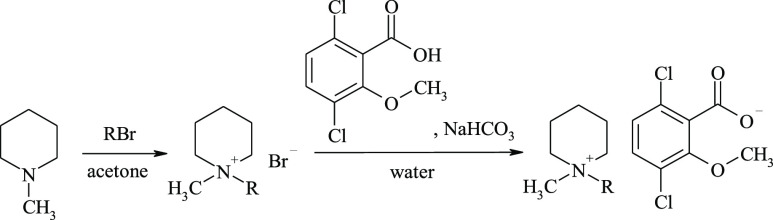
Two-Step Synthesis of Piperidinium-Based HILs

The first step in the preparation of piperidinium ionic liquids with a (3,6 dichloro-2-methoxy)benzoate anion (dicamba) was to synthesize the precursors, 1-alkyl-1-methylpiperidinium bromides. The reaction course is shown in Scheme 1. The quaternization of 1-methylpiperidine with alkyl bromides was carried out in a polar solvent to accelerate the formation of the polar product. The reaction was of the S_N_2-type. The substrates dissolved very well in acetone, from which the resulting product crystallized. The product was filtered and washed with cold ethyl acetate to elute unreacted starting materials. White crystalline solids were obtained. The compounds with C_8_H_17_ to C_10_H_21_ substituents were highly hygroscopic and therefore dried in a vacuum desiccator over a P_2_O_5_ drying agent, whereas the remaining compounds were considerably less hygroscopic. The surfactant content of the precursors of the ionic liquids was determined by a two-phase titration. The yields and melting points of the bromides are given in Table S1 (Supporting Information). During the second stage of the preparation scheme, ionic liquids were obtained by a metathesis reaction. Long-chain quaternary salts have been effectively obtained by performing a metathesis reaction and isolating the synthesis product from the reaction mixture with chloroform;^9^ therefore, the same procedure was adopted in this study. The sodium salt of dicamba has been more effectively obtained using sodium bicarbonate than NaOH^9^ because the inertness of NaHCO_3_ precludes the formation of Hoffman elimination products from the 1-alkyl-1-methylpiperidinium cation in the presence of the residual excess neutralizing agent. The obtained compounds were very viscous liquids. The yield and surfactant content of these compounds were determined analogously to those of the precursors and are given in Table 1.

**Table 1:**
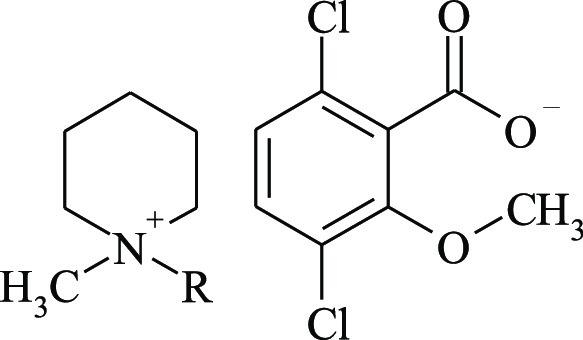
Synthesized 1-Alkyl-1-methylpiperidinium (3,6-Dichloro-2-methoxy)benzoates

C_*n*_	R	surfactant content (%)	yield (%)
C_8_	C_8_H_17_	93.0	85
C_9_	C_9_H_19_	95.5	90
C_10_	C_10_H_21_	98.5	95
C_12_	C_12_H_25_	98.0	97
C_14_	C_14_H_29_	97.0	96
C_16_	C_16_H_33_	94.5	94
C_18_	C_18_H_37_	93.5	93

Proton and carbon nuclear magnetic resonance spectra were obtained to determine the structures of the prepared compounds. Tables 2 and 3 show the signals from the protons and carbon atoms for the representative ionic liquid (C_10_) and its precursor, and for the rest of the compounds in Tables S2–S13, whereas all the NMR spectra are presented in Figures S1–S27 in the Supporting Information. All the expected signals were found in the spectra, and no signals of organic pollutants were observed. The only additional peak that appeared in the proton spectra was that of water. In the ^1^H NMR spectra, the chemical shifts of the protons located closest to the quaternary nitrogen atom differed depending on the anion type. The chemical shifts of the analogous protons of the ionic liquid were all lower than those of the precursor. This result shows higher proton shielding for the dicamba anion than the bromide anion, as has been previously reported.^21^ However, in the ^13^C NMR spectra, the analogous carbon atoms for the ionic liquid, except for 10 and 11, had slightly higher chemical shifts (Table 3) than the precursor.

**Table 2:**
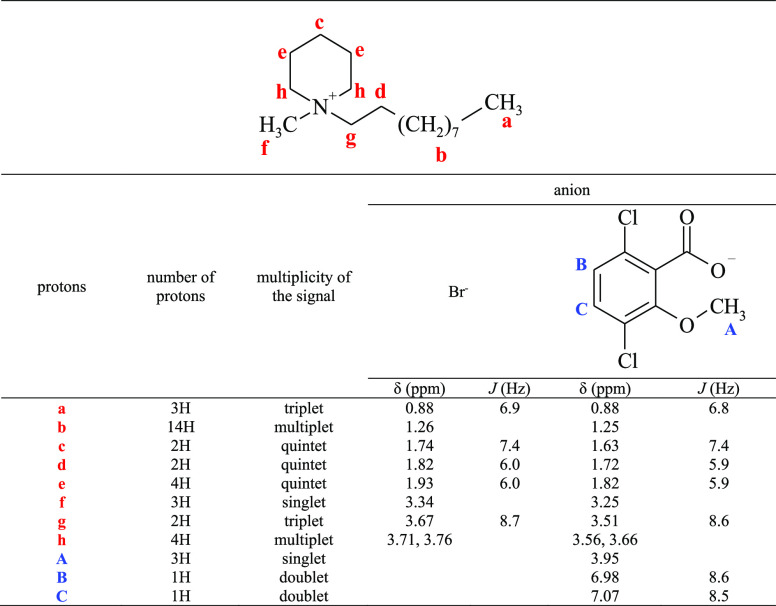
Chemical Shifts (δ) and Coupling Constants (*J*) in the ^1^H NMR Spectra of 1-Decyl-1-methylpiperidinium Bromide and (3,6-Dichloro-2-methoxy)benzoate (CDCl_3_)

**Table 3:**
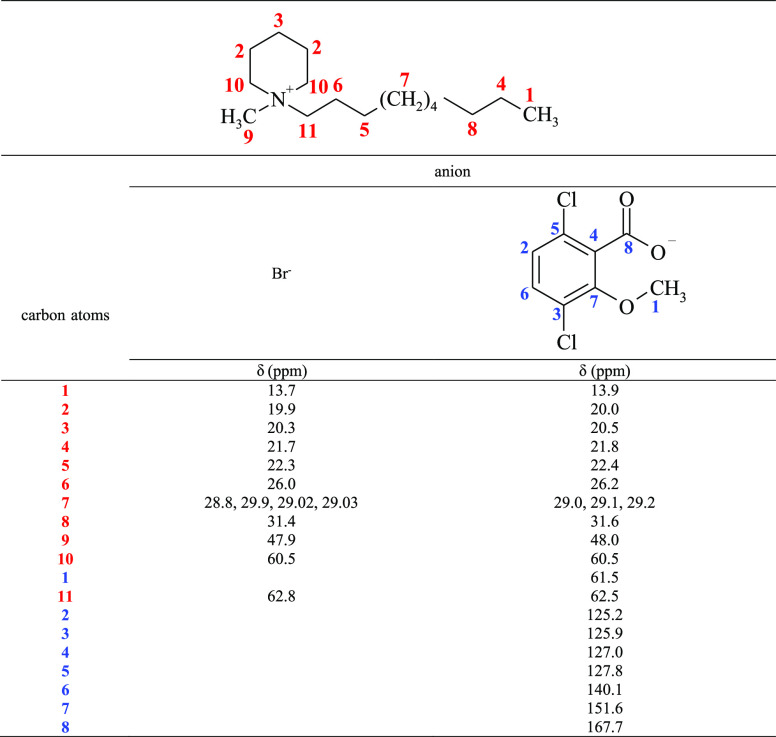
Chemical Shifts (δ) in the ^13^C NMR Spectra of 1-Decyl-1-methylpiperidinium Bromide and (3,6-Dichloro-2-methoxy)benzoate (CDCl_3_)

### Thermal Analysis

The thermal properties of the synthesized ionic liquids were investigated using DSC and TGA. The phase transition temperatures were determined from the DSC thermograms. The crystallization (*T*_c_) and melting (*T*_m_) temperatures were estimated from the maximum values of the exothermic peak on cooling and the endothermic peak on heating, respectively. The cold crystallization (*T*_cc_) temperature was determined from the maximum values of the exothermic change during the heating cycles, and solid–solid transition temperature (*T*_s–s_) was determined from the maximum of the endothermic change during the heating cycles. The glass transition (*T*_g_) was determined as the midpoint of the change in the heat capacity during the heating cycle. The results are presented in Table 4, and representative DSC thermograms of the investigated HILs are presented in Figure S29. Glass transition temperatures were observed for all the synthesized HILs except C_18_. For the C_8_–C_10_ HILs, the glass transition is the only transition observed in the studied temperature range. Crystallization in these HILs is restricted by van der Waals attractive and dispersive interactions between hydrocarbon chains, as well as by multiple rotational modes.^40^ HILs with substituents containing long alkyl chains (C_12_–C_18_) exhibit additional melting and crystallization transitions, such that the structural order increases with the hydrocarbon chain length. This order is related to microphase separation between the ionic and hydrophobic parts of the HIL.^40^ The C_12_ and C_14_ HILs undergo transitions during heating that can be identified as cold crystallization at 24 and 10 °C with melting points at 42 and 31 °C, respectively. The higher homologues (C_16_ and C_18_) melt during heating and crystallize upon cooling. Solid–solid polymorphic transitions appear before the melting transition that are associated with changes in the conformation of the long hydrocarbon chain of the substituent. These conformational changes cause a change in the density of the sample before the melting transition.^41^ Additionally, the longer the alkyl substituent chain is, the higher the melting temperature of the HIL is.

**Table 4:** Glass Transition Temperature (*T*_g_), Cold Crystallization Temperature (T_cc_), Solid–Solid Transition Temperature (*T*_s–s_), Melting Temperature (*T*_m_), and Crystallization Temperature (*T*_c_) of the Obtained ILs Determined from DSC Thermograms (at a Heating/Cooling Rate of 10 °C/min)

C_*n*_	*T*_g_ (°C)	*T*_cc_ (°C)	*T*_s–s1_ (°C)	*T*_s–s2_ (°C)	*T*_m_ (°C)	*T*_c1_ (°C)	*T*_c2_ (°C)	*T*_c3_ (°C)
C_8_	–37.7							
C_9_	–39.2							
C_10_	–48.6							
C_12_	–46.2	25.8			42.8			
C_14_	–39.8	8.7			31.1			
C_16_	–29.0		–0.1	12.9	43.4	–7.3		
C_18_			–1.7	37.3	61.2	–1.0	29.6	36.2

The beginning of the thermal decomposition of the studied HILs was determined as the temperature at which a 5% mass loss occurred. The temperature at which a 50% mass loss occurred was also estimated. The results are presented in Table S14. Generally, all the synthesized HILs are stable over the temperature range of 173–180 °C and are thermally degraded by a multistep process (Figure S29). The HIL thermal stability increases with the length of the hydrocarbon chain of the substituent. An opposite trend has generally been reported in the literature, that is, the temperature at which thermal decomposition occurs decreases with increasing alkyl chain length. There are, however, some reports of trends similar to that presented here.^42,43^ The authors of these latter studies suggest that this trend is caused by progressive fragmentation of hydrocarbon substituents, which leads to less volatile decomposition products. The thermal degradation of the C_8_ and C_9_ HILs was studied. The first step of the degradation of these compounds ends at approximately 250 °C. The exact temperatures are at 252 °C for C_8_ with an 87% mass loss and 253 °C for C_9_ with an 87% mass loss. The second step ends at 420 °C with an 8% mass loss, 430 °C with a 9% mass loss and 420 °C with a 14% mass loss. The C_10_, C_12_, and C_14_ HILs exhibit similar two-step degradation: the first step ends at 251 °C with a 73% mass loss (C_10_), 250 °C with a 72% mass loss (C_12_) and 270 °C with a 74% mass loss (C_14_), followed by a second step in the range of 251–432 °C (23% mass loss), 250–445 °C (20% mass loss), and 270–450 °C (22% mass loss). The thermal stability of the HIL with a longer alkyl chain, i.e., C_16_, is different, i.e., higher, from the other herbicidal ionic liquids. The first step in thermal decomposition ends at the highest temperature of 303 °C with a 70% mass loss, and the second step ends at 435 °C with a 26% mass loss. The thermal decomposition of C_18_ with the longest alkyl chain exhibits similarities to that of C_14_. The first stage of decomposition ends at 285 °C with a 79% mass loss, and the second step ends at 443 °C with a 17% mass loss.

### Surface Properties

Surface tension measurements were carried out to investigate the interactions at the water–air interface and used to determine several parameters, including the CMC, surface tension at the CMC (γ_CMC_), adsorption efficiency (pC_20_), CMC/C_20_ ratio, Gibbs free energy of the adsorption layer (Δ*G*^0^_ads_), excess concentration (Γ_max_), and minimum surface area occupied by a molecule at the interface (*A*_min_), as will be described later. The values of these surface properties for the series of piperidinium ionic liquids are summarized in Table 5.

**Table 5:** Surface Activity of Synthesized HILs

C_*n*_	CMC (mmol/L)	CMC/C_20_	γ_CMC_ (mN/m)	pC_20_	Γ_max_ × 10^6^ (mol/m^2^)	*A*_min_ × 10^19^ (m^2^)	Δ*G*^0^_ads_ (kJ/mol)
C_8_	60.25	6.5	34.4	2.03	6.26	2.65	–13.5
C_9_	24.73	5.9	32.0	2.37	9.82	1.69	–13.6
C_10_	18.50	5.9	36.1	2.50	7.30	2.28	–15.8
C_12_	4.43	4.9	37.5	3.04	6.62	2.51	–18.9
C_14_	1.27	4.7	38.2	3.57	6.82	2.43	–21.9
C_16_	0.42	4.1	37.5	4.00	8.75	1.90	–23.3
C_18_	0.08	1.8	39.8	4.35	8.25	2.01	–25.3

**Figure 1:**
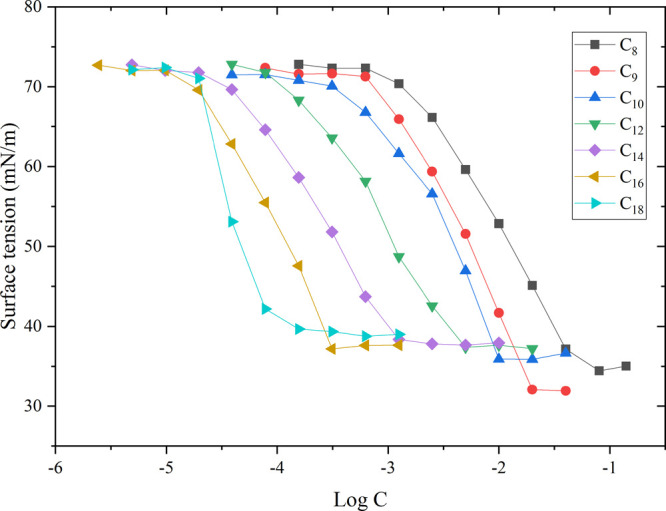
Surface tension versus log concentration of the synthesized HILs at 25 °C.

The surface tension curve for the synthesized HILs is presented in Figure 1. In theory, the surface tension first gradually decreases as the ionic liquid concentration increases. This region is called premicellar,^44^ and the concentration at which premicellar aggregates form is called the critical aggregation concentration. Beyond this region, the surface tension stops changing at a well-defined concentration, known as the CMC. When the concentrations exceed the CMC, the surface tension remains stable and does not change significantly with increasing surfactant concentration. This regime is called the postregion.^45^

In this study, the surface tension of aqueous solutions (γ_CMC_) of the analyzed HILs decreased from the value for water (72.8 mN/m) to plateau at a minimum ranging from 32.0 to 39.8 mN/m. Surface tension measurements for HILs are important as an efficient way of obtaining information on the intrinsic energy involved in the interactions between cations and anions.^46−48^ At constant HIL concentrations, the surface tension increases with the alkyl chain length. The critical surfactant concentration associated with the micellization process is a crucial parameter that can be determined from the surface tension–concentration profile in aqueous solution. The critical micelle concentrations of the analyzed HILs are summarized in Table 5. The CMC appears to decrease with increasing alkyl chain length. The micellization process is the result of two forces. One is the attraction force between compounds with surface activity and water molecules. This force increases the tendency of ionic liquid molecules to localize in the bulk of the solution. Above observation was also observed in 2012 by Tariq et al.^46^ The second force is the repulsion between cyclic groups and water molecules. This force promotes the presence of compound molecules at the air/water interface. The CMCs range from 0.08 to 60.25 mmol/L. The C_18_ HIL has the highest tendency to form micelles at low concentrations. Similarly, to conventional surfactants, increasing the number of carbon atoms in the molecules of the analyzed ionic liquids increases their tendency to form in a water solution. Therefore, increasing the hydrophobicity of the molecules promotes their adsorption at the air–aqueous interface until a saturated state is reached. Note that all the relationships presented above between the CMC and the length of the alkyl substituent follow the Stauff–Klevens rule.^49^

The parameter used to evaluate the surface activity of HILs is the adsorption efficiency (pC_20_). In the literature,^46,50^ higher pC_20_ values have been reported to indicate a higher affinity of HIL molecules for adsorption at the air–water interface. As expected, increasing the length of the alkyl chains causes an increase in the hydrophobicity of the compound molecules. This result is reflected in the increase of the pC_20_ values. Correspondingly, the highest pC_20_ of 4.35 was obtained for compound C_18_. In general, the adsorption process can also be characterized using the CMC/C_20_ ratio.^48^ Increasing the length of the alkyl group caused the CMC/C_20_ ratio to decrease from 6.5 to 1.8. The negative values of Δ*G*^0^_ads_ presented in Table 5 show that micelle formation is a spontaneous process. A similar situation was noted in 2012 by Zdziennicka et al.^50^ Δ*G*^0^_ads_ decreases with the increase in the length of the alkyl chain for all the ionic liquids, showing that the aggregation is driven by the hydrophobic effect.

The values of *A*_min_ for the HILs increase from 1.69 to 2.65 × 10^–19^ m^2^, whereas Γ_max_ ranges from 6.26 to 9.82 × 10^–6^ mol/m^2^. Interestingly, high values of the maximum surface excess and low values of the minimum surface area suggest that surface-active ionic liquid molecules have a high tendency to adsorb at various interfaces, i.e., at air–water or solid–water interfaces. Consequently, knowledge of the tendency of HILs to adsorb at different interfaces can improve their utility for many applications, for example, as agricultural herbicides.^51,52^

The wettability of a plant surface is assessed by the CA. Observation of static CA values is essential for the application of appropriate chemical formulations of spray solutions. CA values depend on factors that are primarily related to the morphology of leaves (the adaxial and abaxial sides of a leaf).^53^ All the synthesized HILs were analyzed in terms of the behavior of spray solutions on the leaf surfaces of three popular species of weeds that are widespread in Polish fields: cornflower (*Centaurea cyanus* L.), winter rapeseed (*Brassica napus* L.) and white mustard (*Sinapis alba* L.). The leaves of common wheat (*Triticum aestivum* L.) were also tested. Dicash (dicamba dimethylammonium salt, 480 g/L) contains an active ingredient analogous to the investigated HILs and was used as a reference.

**Figure 2:**
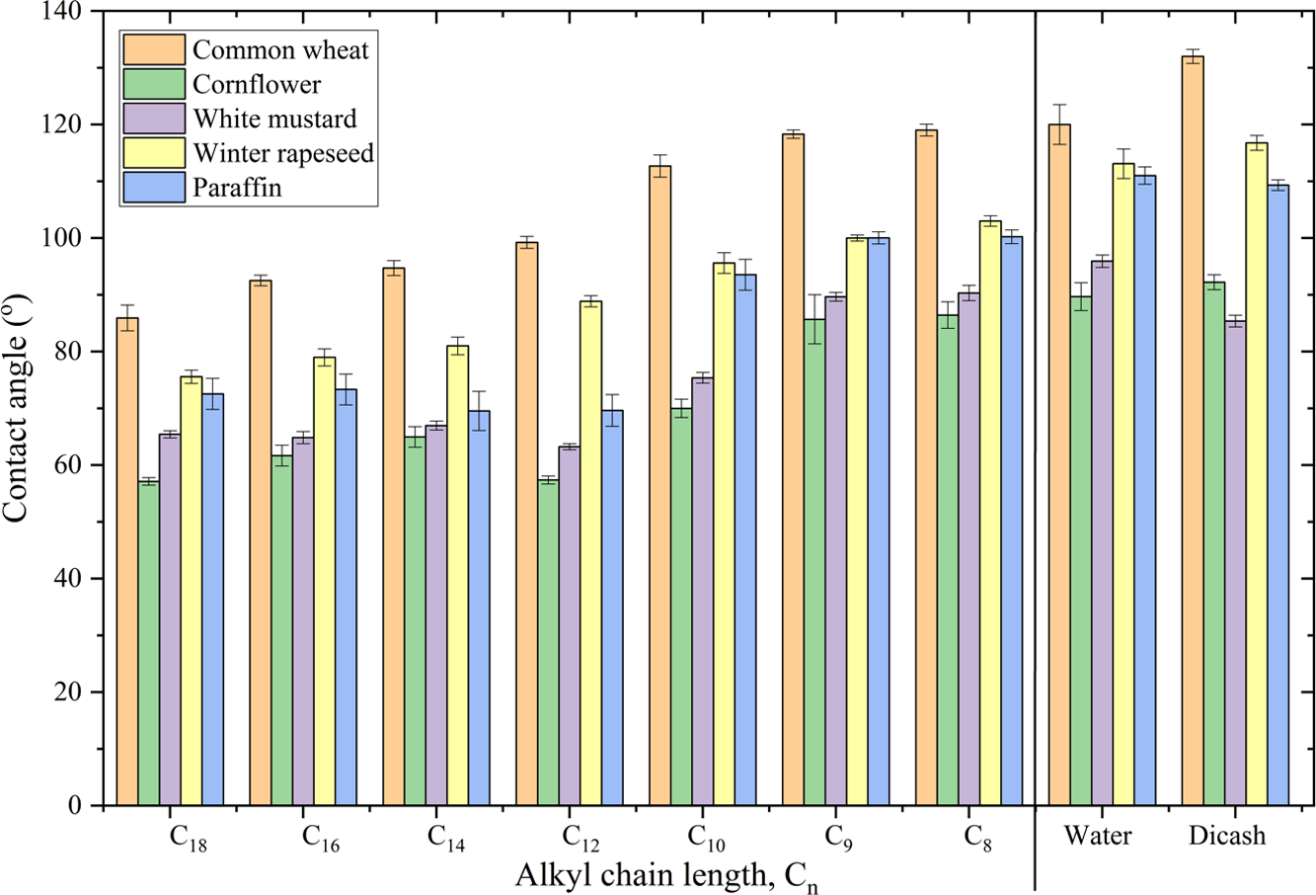
Static CA values on the adaxial surface of leaves (more details are provided in Table S3, Supporting Information).

The results presented in Figure 2 and Table S15 show that the wettability of paraffin and the leaf surfaces by the studied HILs depended on the alkyl chain length. In general, compound C_8_ had the lowest ability to wet the analyzed surfaces (the CA values were as follows: common wheat, 119.0°; winter rapeseed, 103.0°; cornflower, 86.4°; white mustard, 90.3°; and paraffin, 100.0°). The highest wettability was recorded for the C_18_ HIL (the CA values were as follows: common wheat, 85.9°; winter rapeseed, 75.6°; cornflower, 57.1°; white mustard, 65.4°; and paraffin, 73.0°).

**Figure 3:**
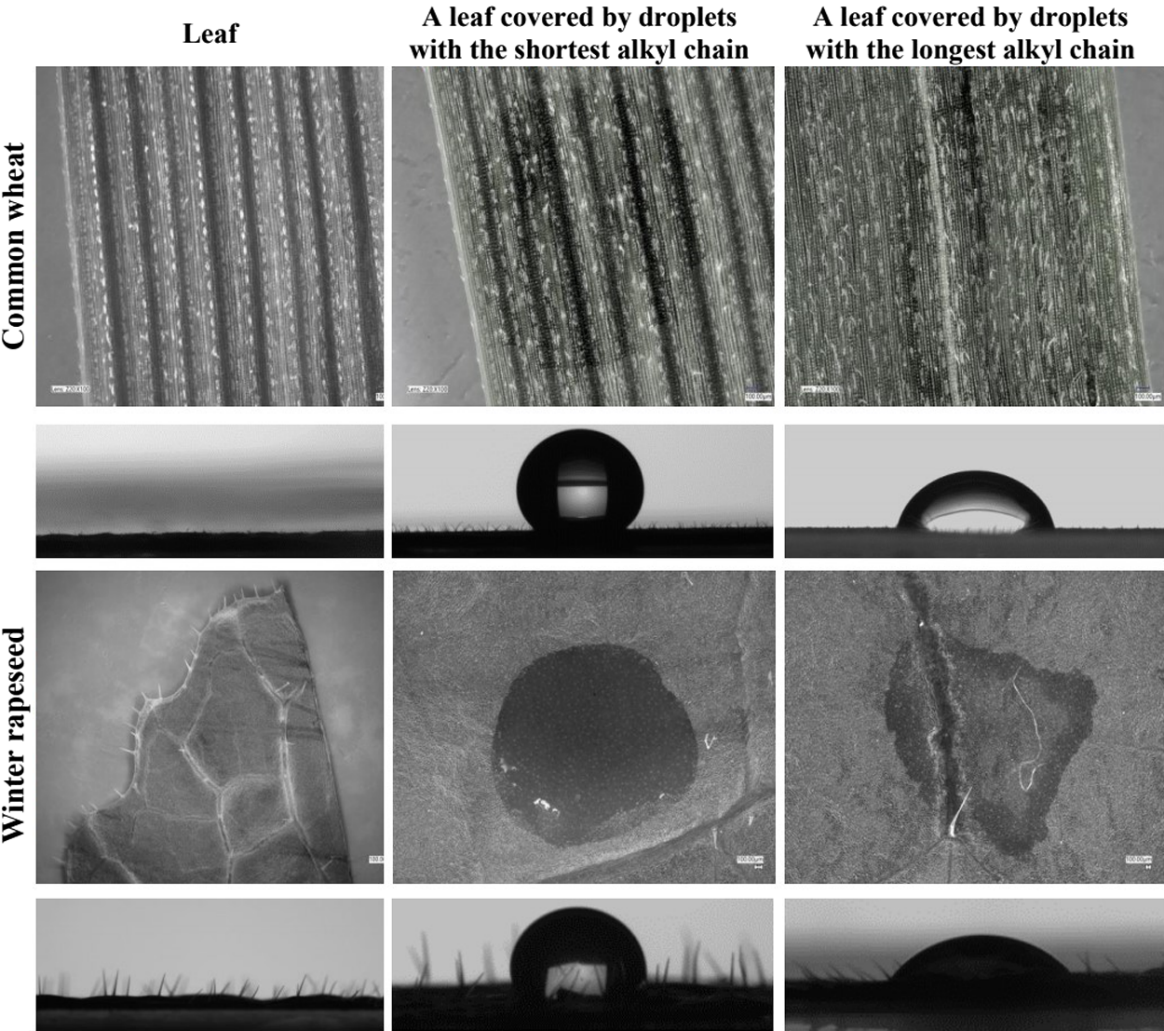
Digital microscope images for the adaxial side of leaves. The images were obtained under magnification. The bar represents 100 μm.

Special attention should be given to the biological aspects of the wettability analysis of the leaf surfaces (see Figure 3). Generally, the variation in the CA values of the HILs was due to differences in the plant morphologies, which are determined by the plant chemical compositions.^54,55^ Common wheat belongs to the *Poaceae* family,^56^ cornflower belongs to the *Asteraceae* family, whereas white mustard and winter rapeseed are part of the *Brassicaceae* family.^57−59^ It should be emphasized that the CA for a given substance is related to the morphological structure of the plant, that is, the adaxial and abaxial surfaces of the leaf. Theoretically, the two sides of a leaf are morphologically different.^60,61^ That is, the sides of a leaf have different degrees of hydrophilicity and hydrophobicity. The exact CA values on the adaxial and abaxial leaf surfaces are summarized in the Supporting Information (Table S15). The CA values only differed slightly between the leaf sides. This result indicates comparable performance for piperidinium-based herbicidal ionic liquids for the two leaf sides.

Paraffin has a hydrophobic surface, which can however serve as a model surface to simulate biological systems. The values of CA for all the solutions on paraffin ranged from 73.0 to 100.0°. Thus, we concluded that paraffin is not an ideal surface for simulating leaf surfaces.^21,38^

A comparison of the values of CA recorded for water and the Dicash solution showed a difference in the wettability of the leaf surfaces of weeds and paraffin. Compared to the CA values recorded for the C_18_ HIL solution, the values of CA of the Dicash solution were markedly higher on the surfaces of common wheat or winter rapeseed (by up to 40°) and cornflower or white mustard (by up to 20°), that is, the Dicash solution exhibited worse wetting properties. It follows that the presence of a cation with surface properties increased the wettability of the surfaces analyzed. A similar relationship was observed when pure aqueous solutions were used.

**Figure 4:**
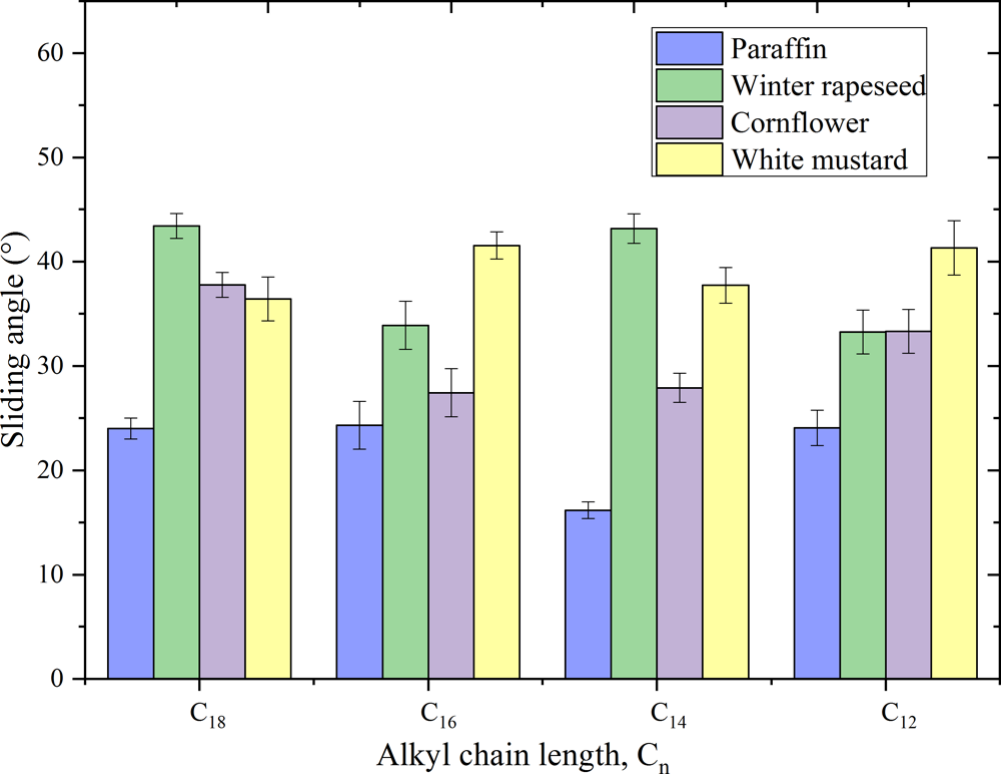
SAs of HILs on the adaxial side of the weed leaf surface and paraffin.

Static CA values have been commonly used as a measure of surface hydrophobicity but are not sufficient for evaluating the sliding ability of drops on surfaces.^62−64^ Thus, SA values are determined to analyze the behavior of a drop on a leaf and, more specifically, drop mobility on a biological surface.^65^Figure 4 shows the measured SA values on the adaxial side of the weed leaf surface and paraffin.

A distinct trend cannot be identified in the SA values of the HILs on cornflower, winter rapeseed, and white mustard leaves. However, a drop of HILs with short alkyl chains (C_8_–C_10_) could not slide down a leaf even at 90°, which was the maximum limit of the tilt angle of the sampling stage.^66^ Thus, the surface microstructure, i.e., the roughness of a micropillar-structure, undoubtedly plays a significant role in the sliding behavior of liquid drops on plant leaves.^65^ Moreover, there is an important distinction between paraffin and the surfaces of weeds. The data (Table S16) showed that the drops slid on paraffin, regardless of the quantity of HILs used to wet the test surface.

These results are preliminary indications of the applicability of HILs to agriculture by identifying a crucial issue that, to the best of our knowledge, has not been sufficiently reported in the literature.

### Characterization of Aggregates: Zeta Potential Measurement

The zeta potential (ζ) is a useful parameter for determining the chemical charge of micelles formed in IL solutions. Positive zeta potentials were measured for the studied HILs (except for compounds C_8_ and C_9_, which exhibited negative ζs). Therefore, the micelles possessed a positive surface electric charge at the CMC.

**Figure 5:**
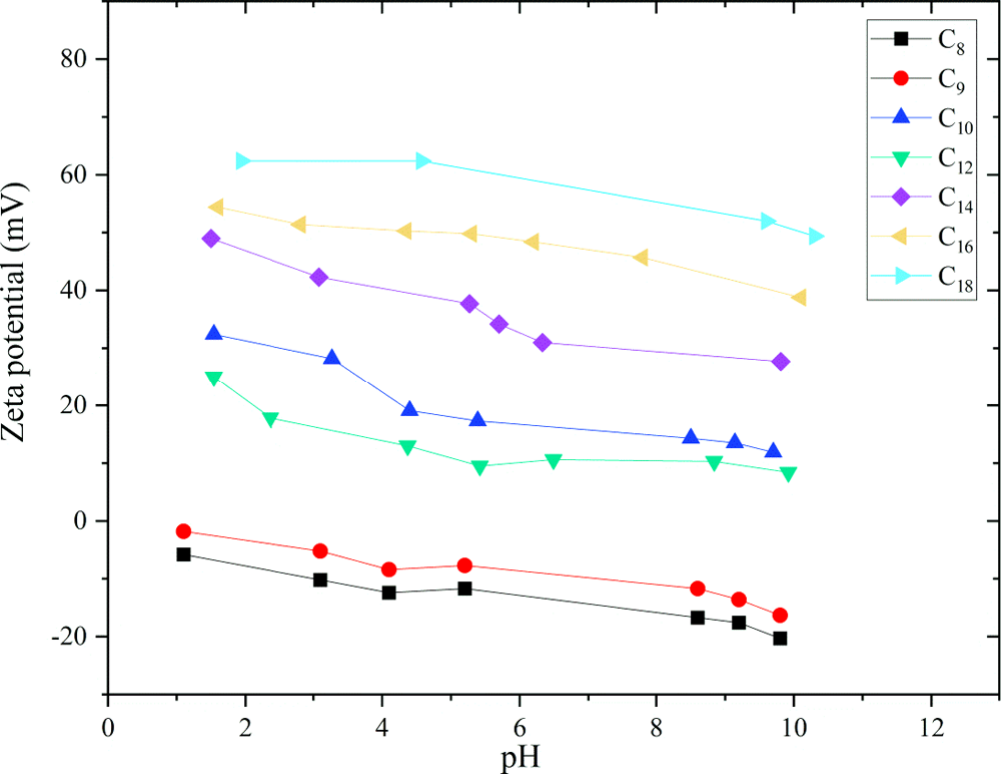
Zeta potential of aggregates of the analyzed HILs.

In the literature, zeta potentials have been reported to depend on the length of the alkyl chain of HILs (Figure 5). In general, negative zeta potentials are observed for short alkyl chains (C_8_–C_10_), and positive zeta potentials are observed for long chains (C_12_–C_18_).^67^ The piperidinium-based HILs followed this trend, except for the compound with an alkyl substituent containing 10 carbon atoms.

### AFM Topographic Analysis

**Figure 6:**
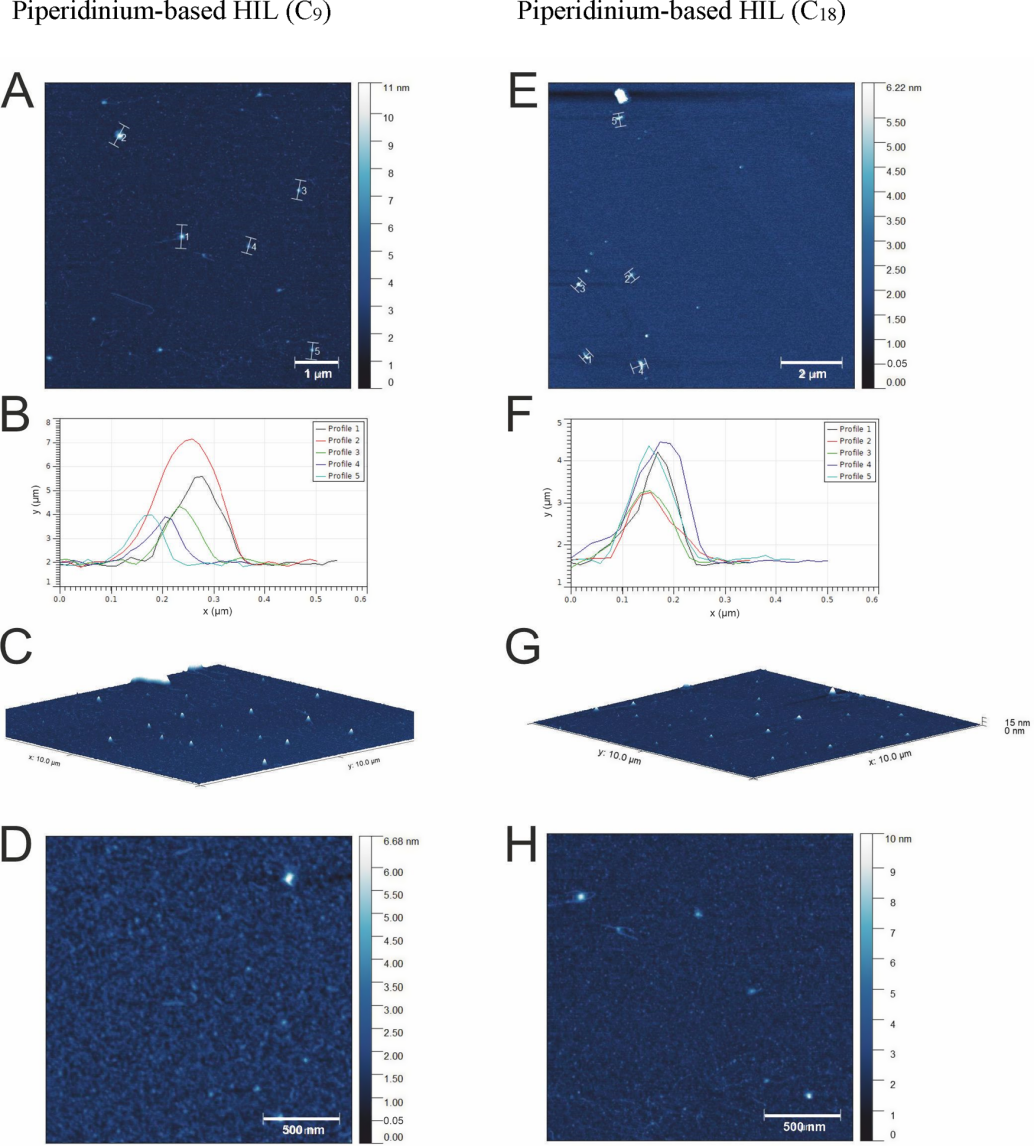
AFM results for piperidinium-based HILs deposited on mica surfaces, showing the surface coverage by piperidinium-based molecules with different alkyl chain lengths (C_9_ and C_18_). (A, E) Topography of selected areas for piperidinium-based HILs with different alkyl chains. (B, F) Five profile curves for selected round deposits. (C, G) 3D view of the test surfaces. (D, H) Topography of selected areas for piperidinium-based HILs with small surface areas.

**Figure 7:**
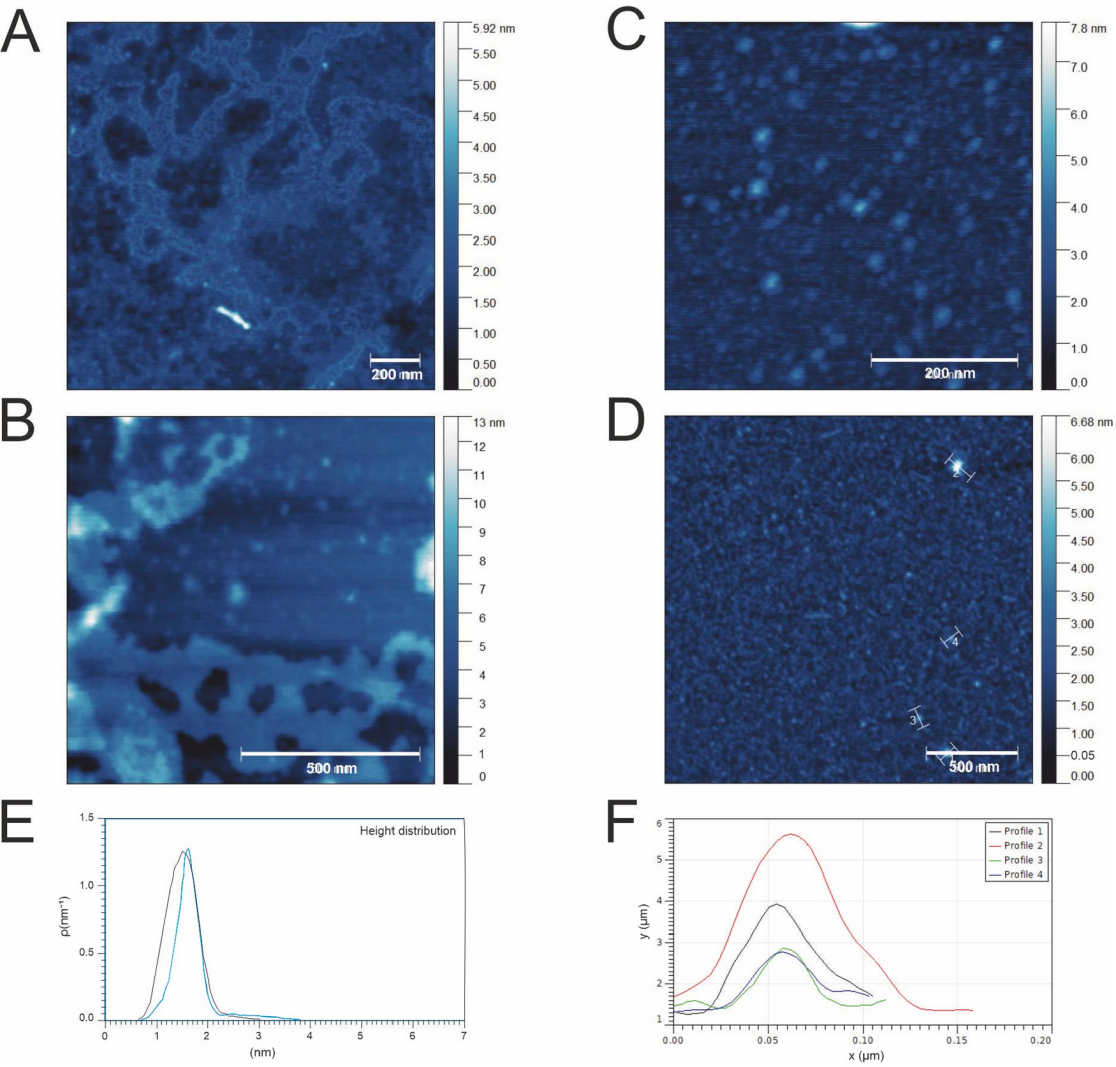
AFM studies of morpholinium-based HILs (A, B) and piperidinium-based HILs (C, D) deposited on mica surfaces. (E) Height distribution of selected samples with different cations (black curve—morpholinium-based HILs, blue curve—piperidinium-based HILs). (F) Selected profile curves for topography D with round deposits.

The potential application of HILs to plant protection is determined by both the physical properties and adherence to the leaf surface of HILs. AFM imaging was applied to characterize these properties, whereby the topography of the tested ionic liquids deposited on the mica surface was visualized. Selected topographic images of deposits of the studied HILs are presented in Figures 6 and 7. To investigate the differences resulting from the alkyl chain length in the investigated piperidinium-based HILs, AFM imaging was performed on HILs containing C_9_ and C_18_ chains with the same dicamba anion. Additionally, the coverage of the dried mica surface of the piperidinium-based HILs was compared against those of morpholinium-based HILs that were measured in a previous study.^21^

The topography of a large surface (see Figure 6A and E) covered with a piperidinium derivative of a short alkyl chain (C_9_) at the same concentration used for the leaf tests shows a dense distribution of symmetric round deposits (micelles) on the surface and is compared against the distribution of similar structures observed for a HIL with a longer alkyl chain (C_18_).

By comparison, the smaller field of view of the test area for the same samples (Figure 6D,H) shows more precise and homogeneous coverage of the mica surface. The surface coverage is almost uniform. Thus, HILs with a long alkyl chain provide more effective surface coverage than compound with a short chain (C_9_).

The results of the AFM studies on piperidinium-based HILs were compared with those described in our previous work.^21^Figure 7 shows the results for herbicides based on dicamba anions with similar alkyl chain lengths (C_9_ and C_10_) and different cations. The morpholinium derivatives containing the dicamba anion show irregular surface coverage due to island formation and the absence of micellar deposits. The observed height distribution values suggest similar heights for the structures formed from both the morpholinium and piperidinium derivatives. These results show that the piperidinium derivatives enable more homogeneous surface coverage than the morpholinium derivatives.^21^

### Phytotoxicity

**Figure 8:**
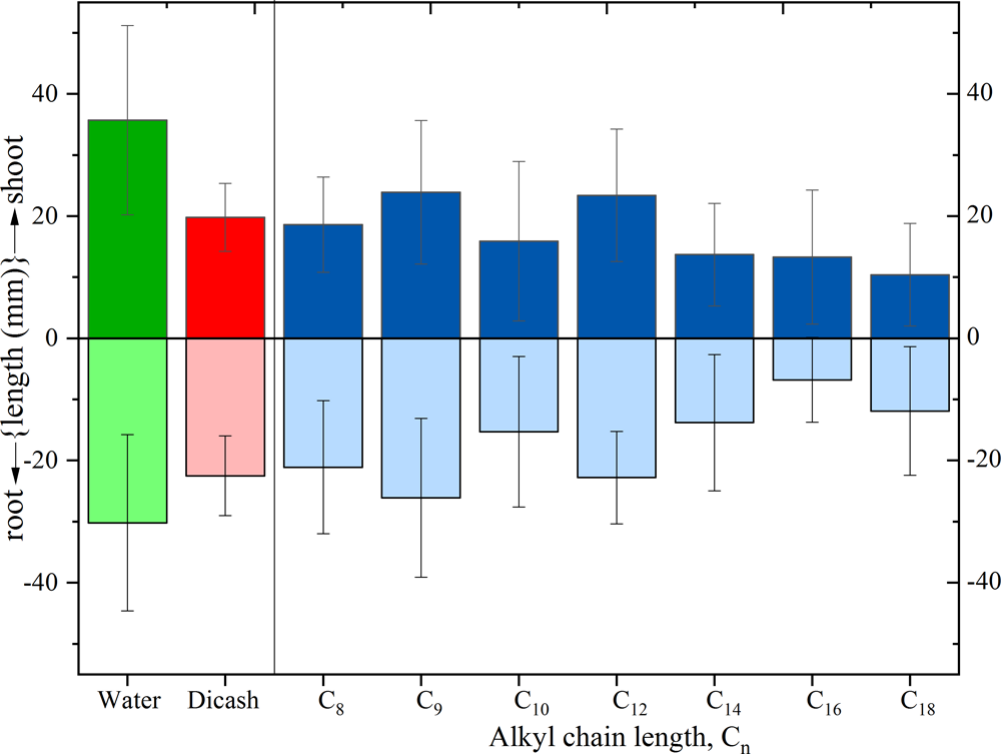
Average root and shoot length for seedlings cornflower (*Centaurea cyanus* L.) in sand with the addition of 1-alkyl-1-methylpiperidinium (3,6-dichloro-2-methoxy)benzoates and Dicash.

To determine the phytotoxicity of synthesized HILs, germination tests were performed. Cornflower (*Centaurea cyanus* L.) was used, which is a popular weed and a model dicotyledonous plant. Sterilized sand was used as a substrate to eliminate the impact of additional soil components. Plants could only get their nutrients from their reserve materials. Deionized water was used as a control, while a commercial product—Dicash—was used as a comparative herbicide. The effects of the ionic liquids and the comparative herbicide are shown in Figure 8 and in Figures S30–S36.

All the synthesized piperidinium-based ionic liquids retained their phytotoxicity, although the activity of the ionic liquids containing 8, 10, 14, 16, and 18 carbon atoms in the alkyl substituent of the cation is significantly better than that of the comparative preparation. The 1-hexadecyl-1-methylpiperidinium (3,6-dichloro-2-methoxy)benzoate) (C_16_) proved to be the best in inhibiting the growth of the root, while the 1-methyl-1-octadecylpiperidinium (3,6-dichloro-2-methoxy)benzoate) (C_18_) was the best in inhibiting the growth of the shoot.

## Supporting Information Available

^1^H and ^13^C NMR spectra with characterization, the results of thermal stability of obtained HILs; results of the static contact angles for the adaxial and abaxial sides of leaves and for paraffin, the sliding angles for the adaxial and abaxial sides of leaves; the figures of TG and DTG curves of the investigated HILs (PDF)
