# Supplementary material for: Synthesis and Surface Properties of Piperidinium-Based Herbicidal Ionic Liquids as a Potential Tool for Weed Control
Source: J Agric Food Chem. 2023 Mar 6;71(11):4550–60. doi: 10.1021/acs.jafc.3c00356 (PMC10037321; doi:10.1021/acs.jafc.3c00356)
Supplement: Supplementary file 1 — jf3c00356_si_001.pdf [file jf3c00356_si_001.pdf]

# SUPPORTING INFORMATION

# Synthesis and surface properties of piperidinium-based herbicidal ionic liquids as a potential tool for weed control

*Marta Wojcieszak,*[*,†] *Anna Syguda,*[†] *Aneta Lewandowska,*[†] *Agnieszka Marcinkowska,*[†]

*Katarzyna Siwińska-Ciesielczyk,*[†] *Michalina Wilkowska,*[‡] *Maciej Kozak,*[‡]

*Katarzyna Materna,*[†]

[†] Poznan University of Technology, Faculty of Chemical Technology, Berdychowo 4, 60-965, Poznan, Poland.

[‡] Department of Biomedical Physics, Faculty of Physics, Adam Mickiewicz University in Poznań, Uniwersytetu Poznańskiego 2, Poznan 61-614, Poland.

e-mail: marta.d.wojcieszak@doctorate.put.poznan.pl

## IDENTIFICATION OF COMPOUNDS

**Table S.1.** Synthesized 1-alkyl-1-methylpiperidinium bromides

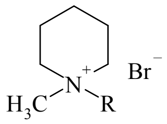


| $C_n$ | surfactant content (%) | yield (%) | melting point (°C) |
|---|---|---|---|
| $C_8$ | 96.5 | 86 | 191.6-192.4 |
| $C_9$ | 98.0 | 82 | 200.0-201.5 |
| $C_{10}$ | 99.0 | 81 | 192.6-192.8 |
| $C_{12}$ | 96.5 | 91 | 194.6 (decomposition) |
| $C_{14}$ | 98.0 | 93 | 196.0 (decomposition) |
| $C_{16}$ | 97.0 | 92 | 192.5 (decomposition) |
| $C_{18}$ | 97.0 | 95 | 193.2 (decomposition) |

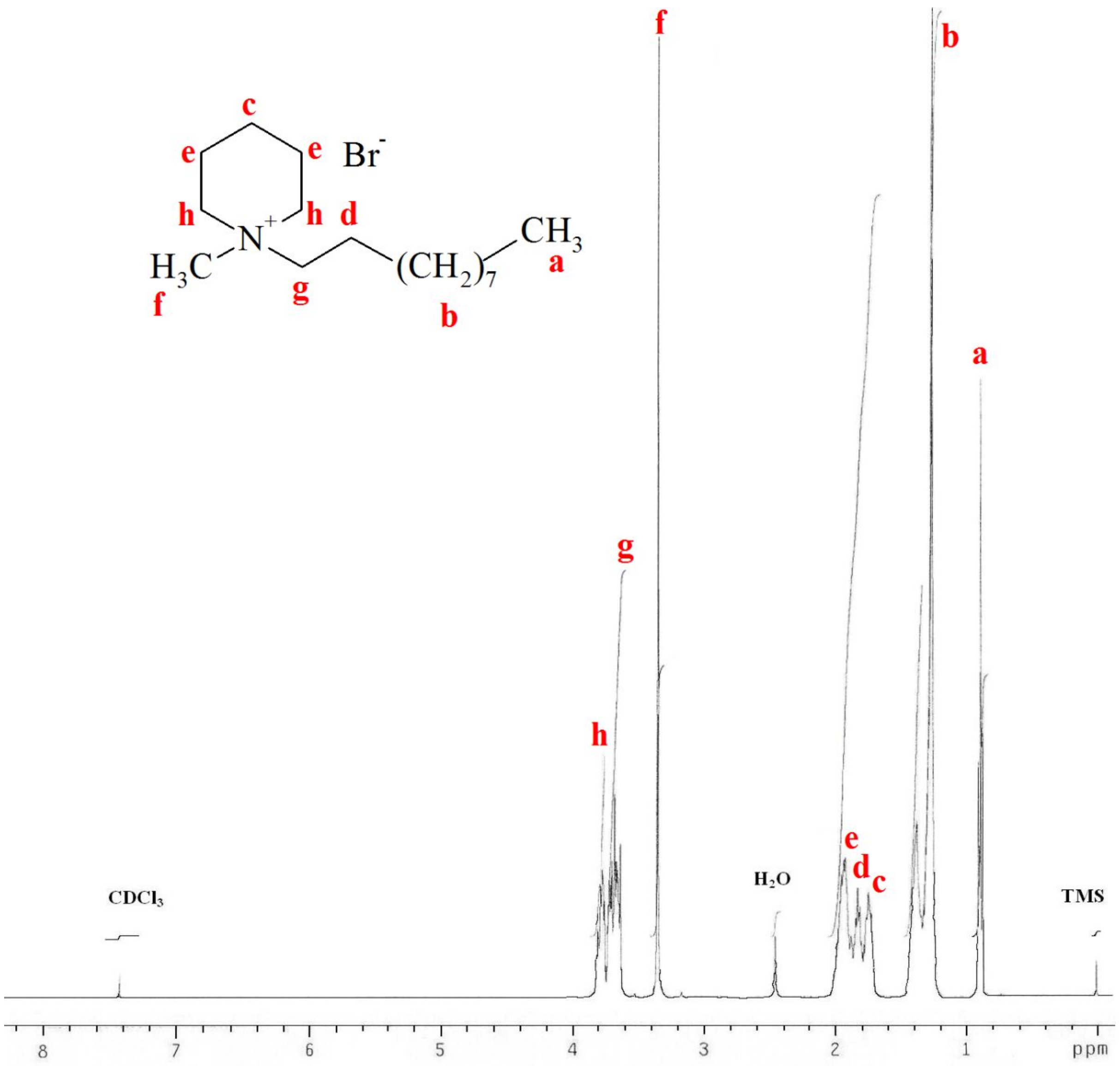


**Figure S.1.** $^{1}$H NMR spectrum of 1-decyl-1-methylpiperidinium bromide.

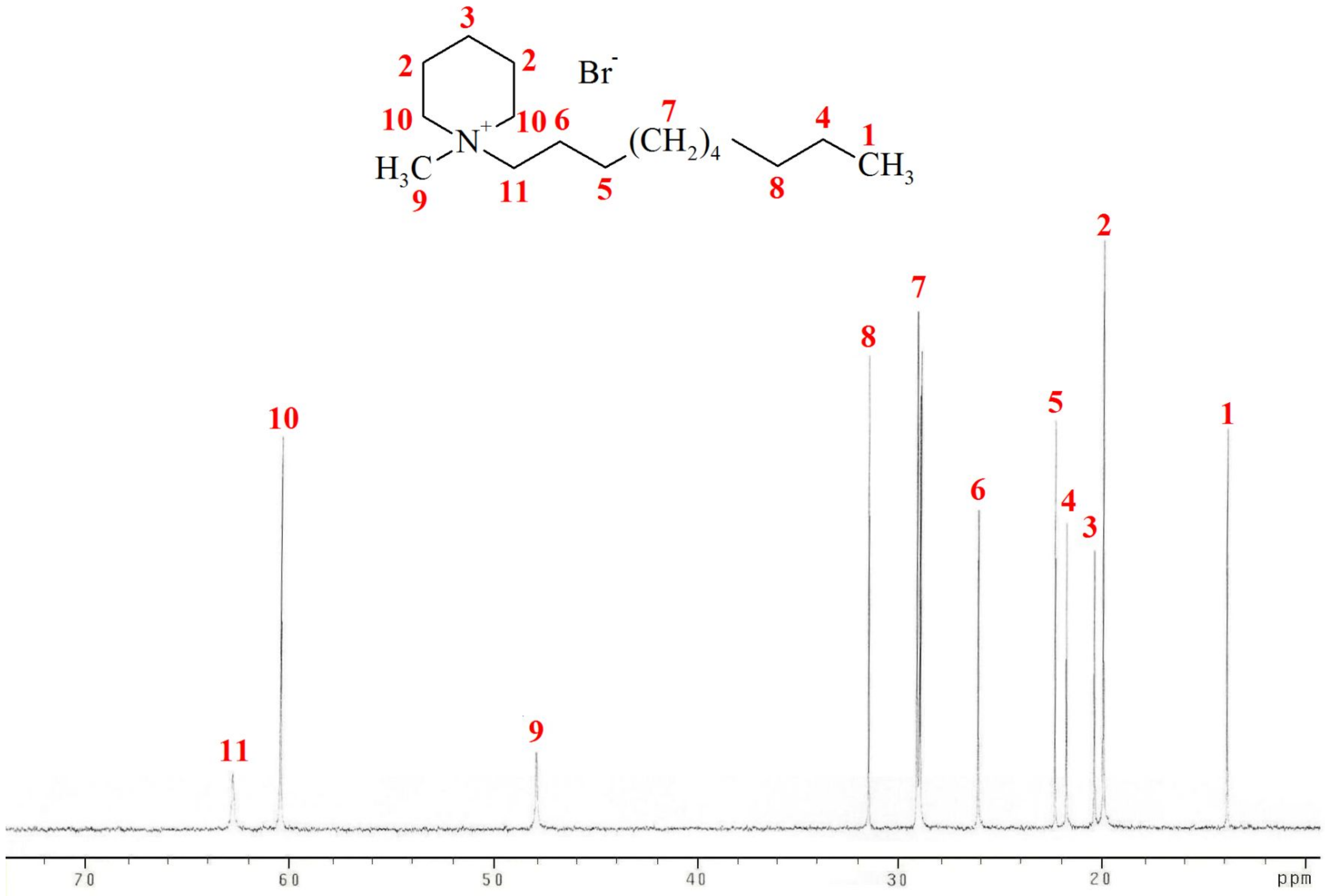


**Figure S.2.** $^{13}C$ NMR spectrum of 1-decyl-1-methylpiperidinium bromide.

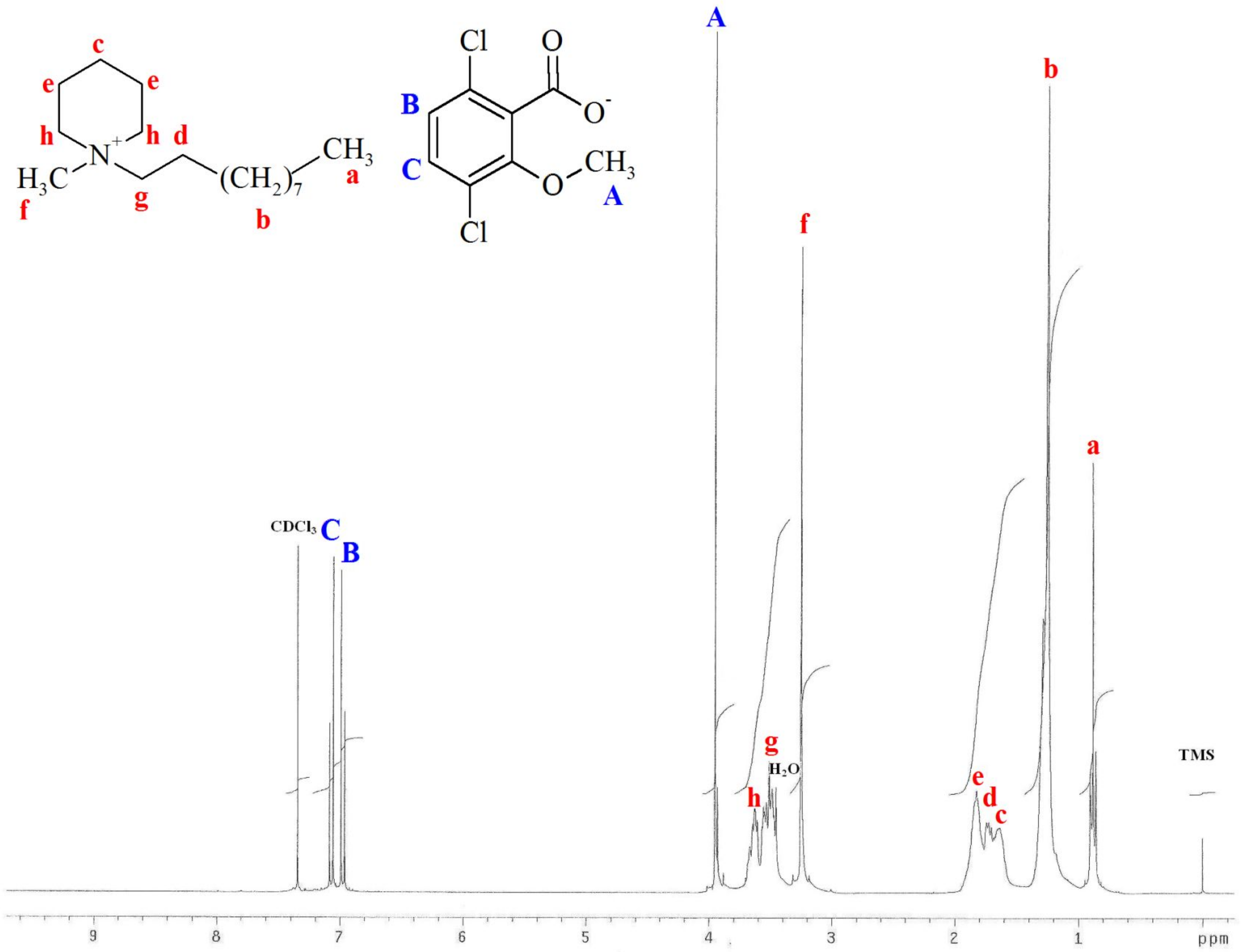


**Figure S.3.** $^1$H NMR spectrum of 1-decyl-1-methylpiperidinium (3,6-dichloro-2-methoxy)benzoate.

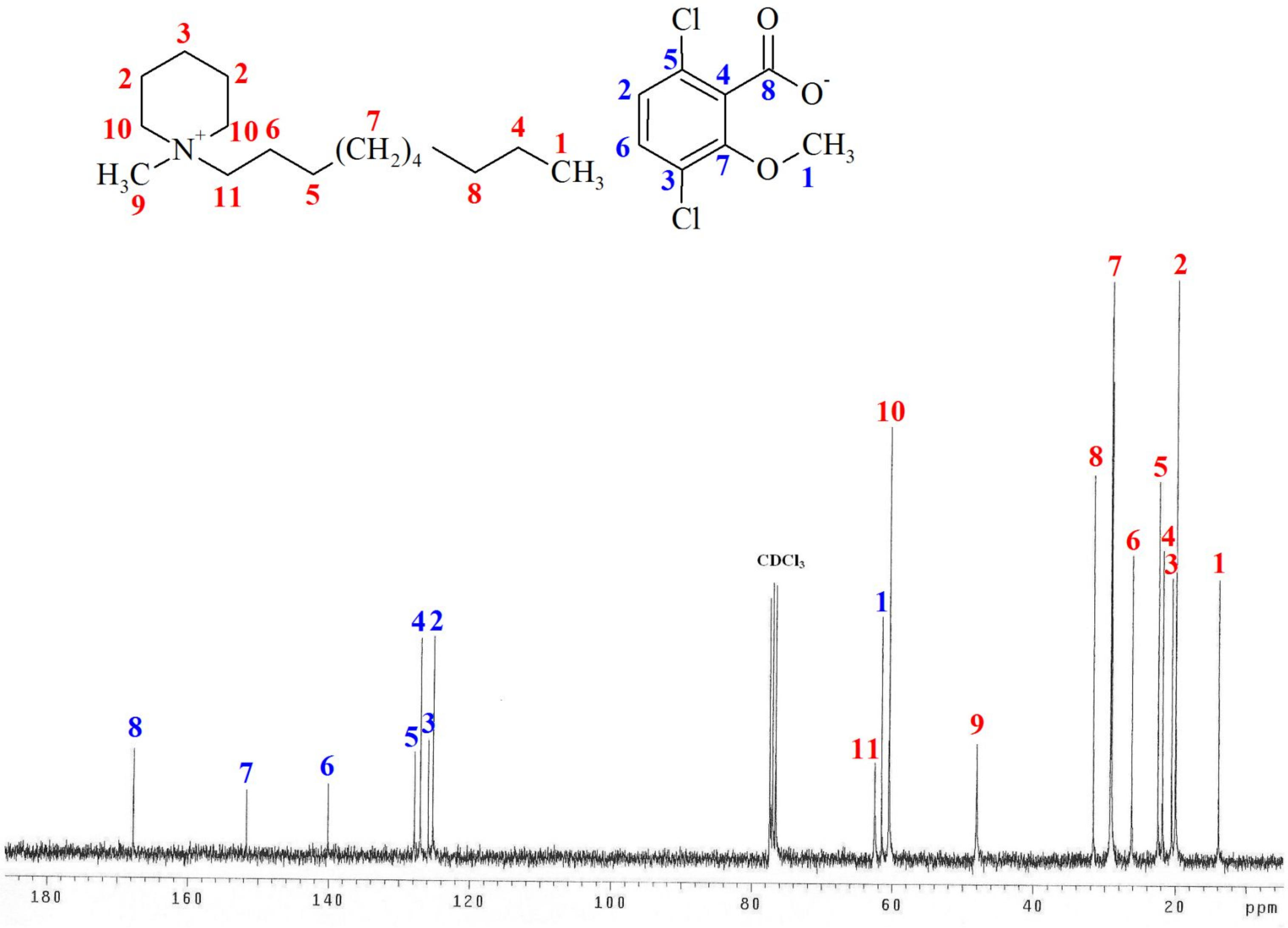


**Figure S.4.** $^{13}C$ NMR spectrum of 1-decyl-1-methylpiperidinium (3,6-dichloro-2-methoxy)benzoate.

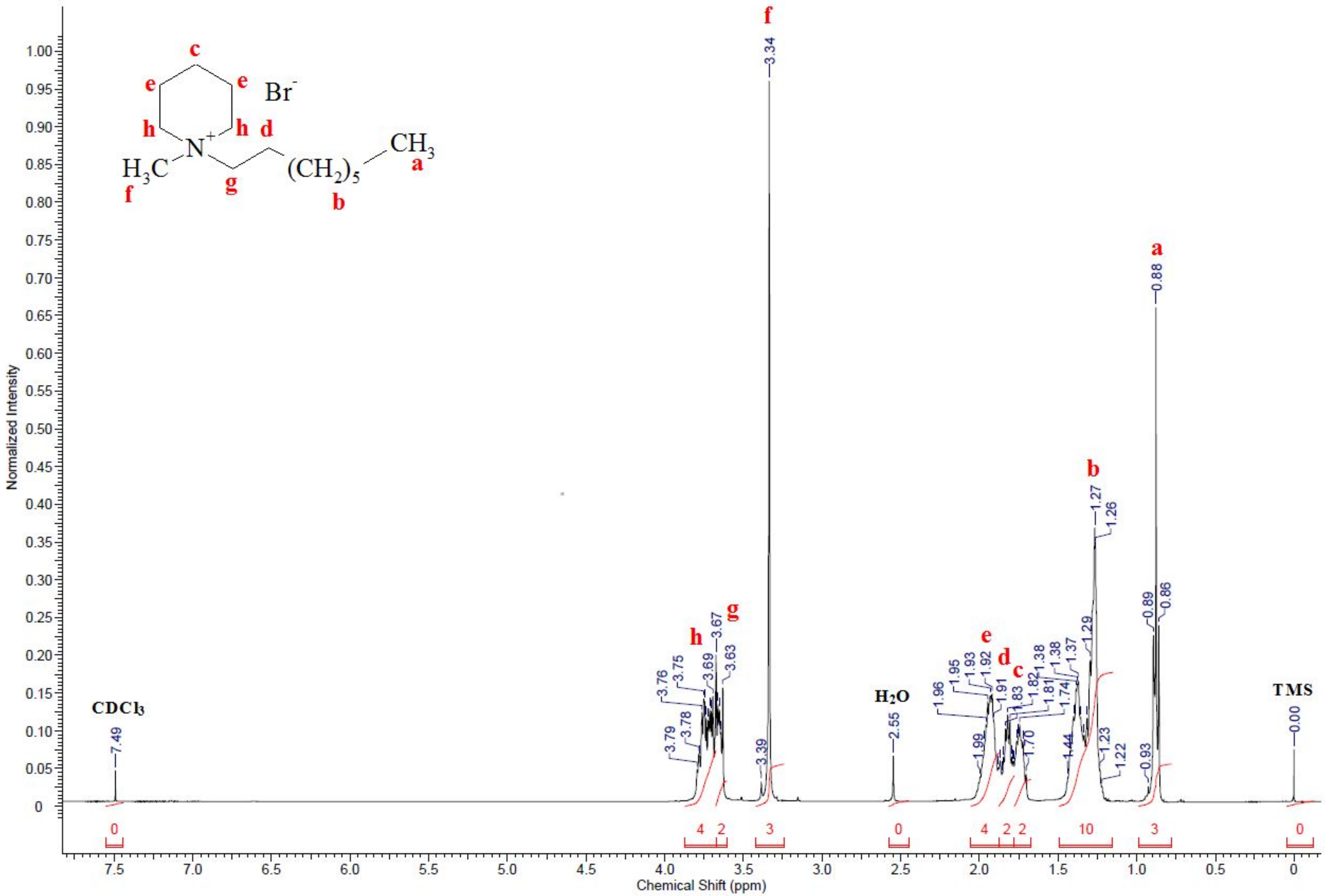


**Figure S.5.** $^{1}$H NMR spectrum of 1-methyl-1-octylpiperidinium bromide.

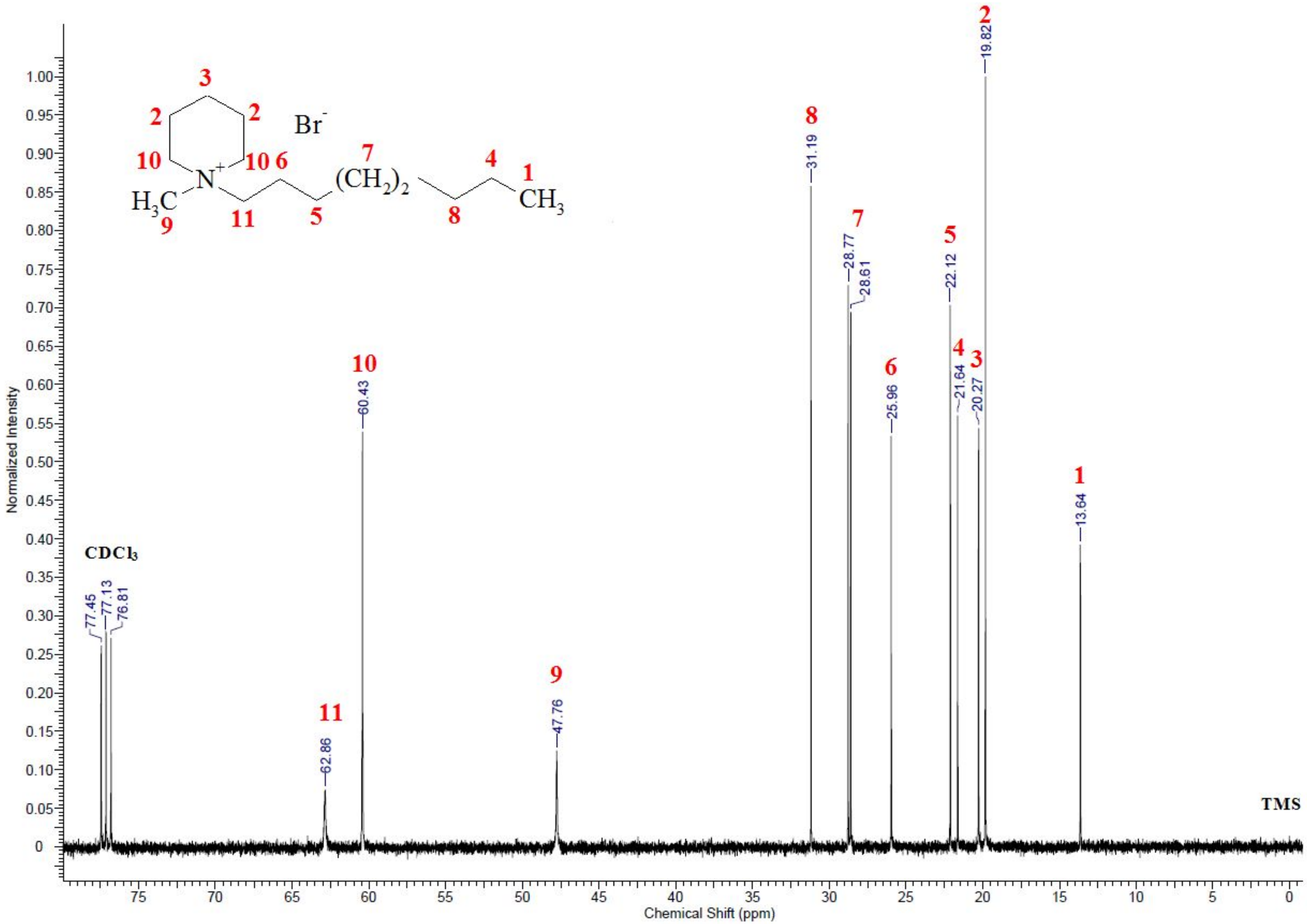


**Figure S.6.** $^{13}C$ NMR spectrum of 1-methyl-1-octylpiperidinium bromide.

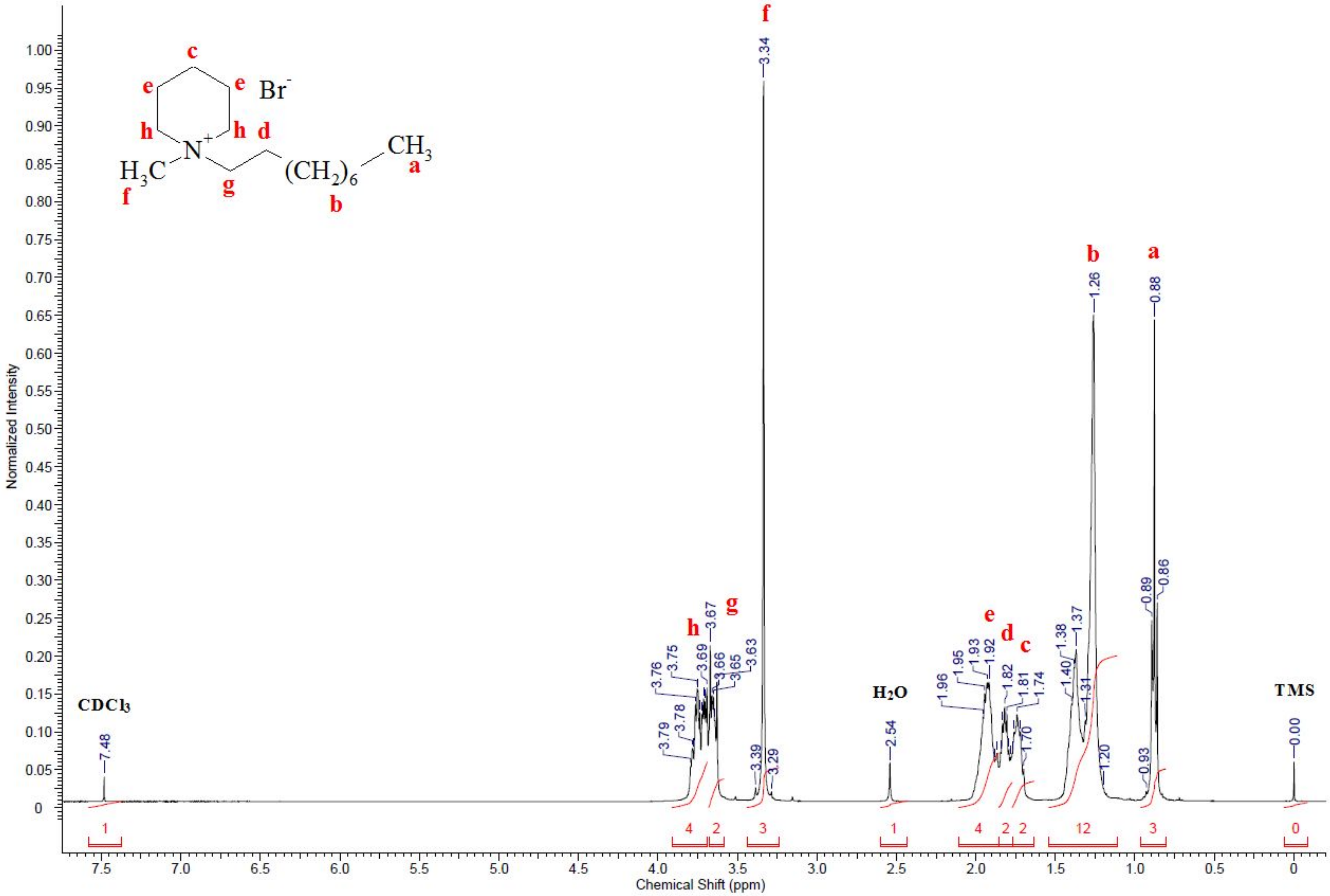


**Figure S.7.** $^{1}$H NMR spectrum of 1-methyl-1-nonylpiperidinium bromide.

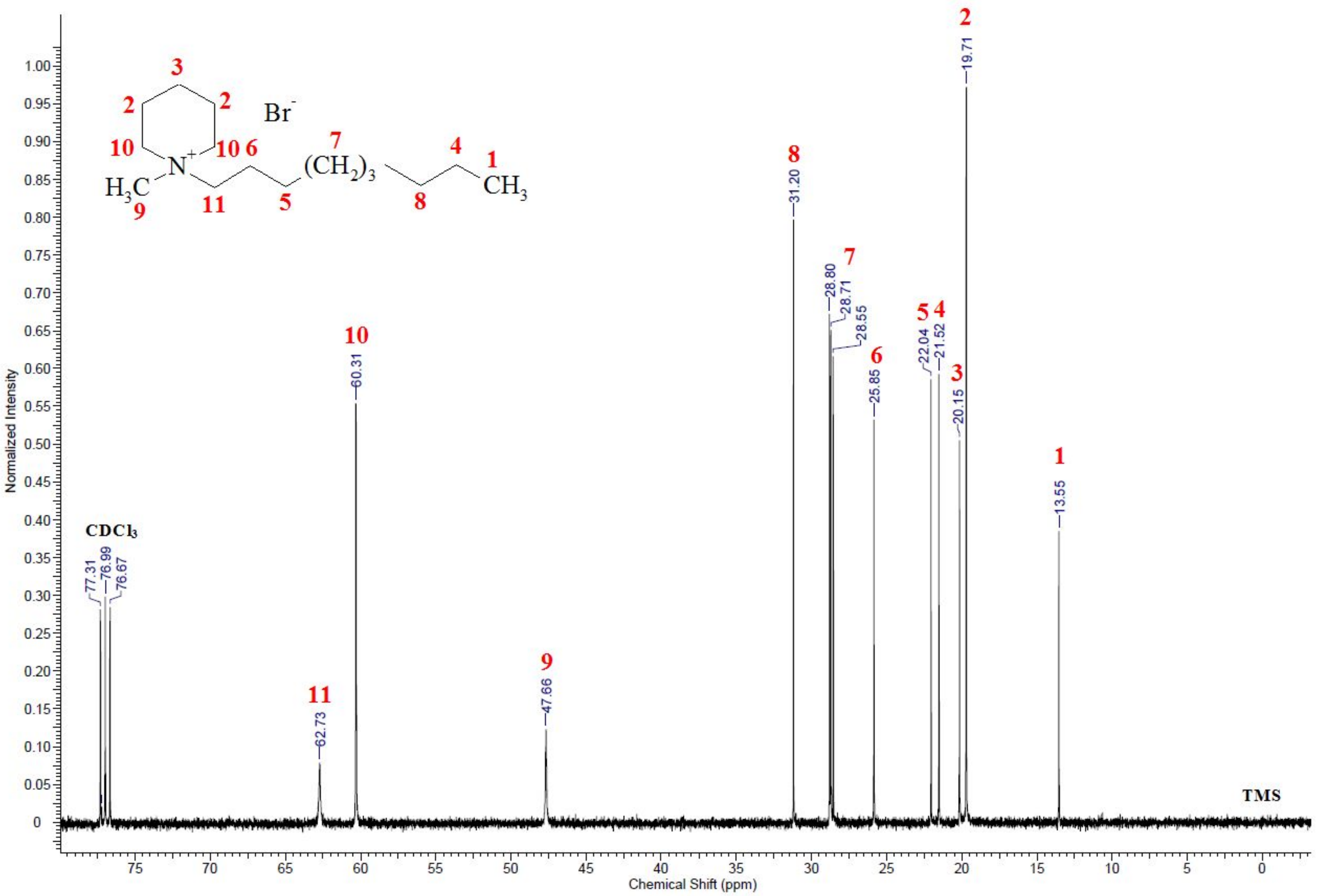


**Figure S.8.** $^{13}C$ NMR spectrum of 1-methyl-1-nonylpiperidinium bromide.

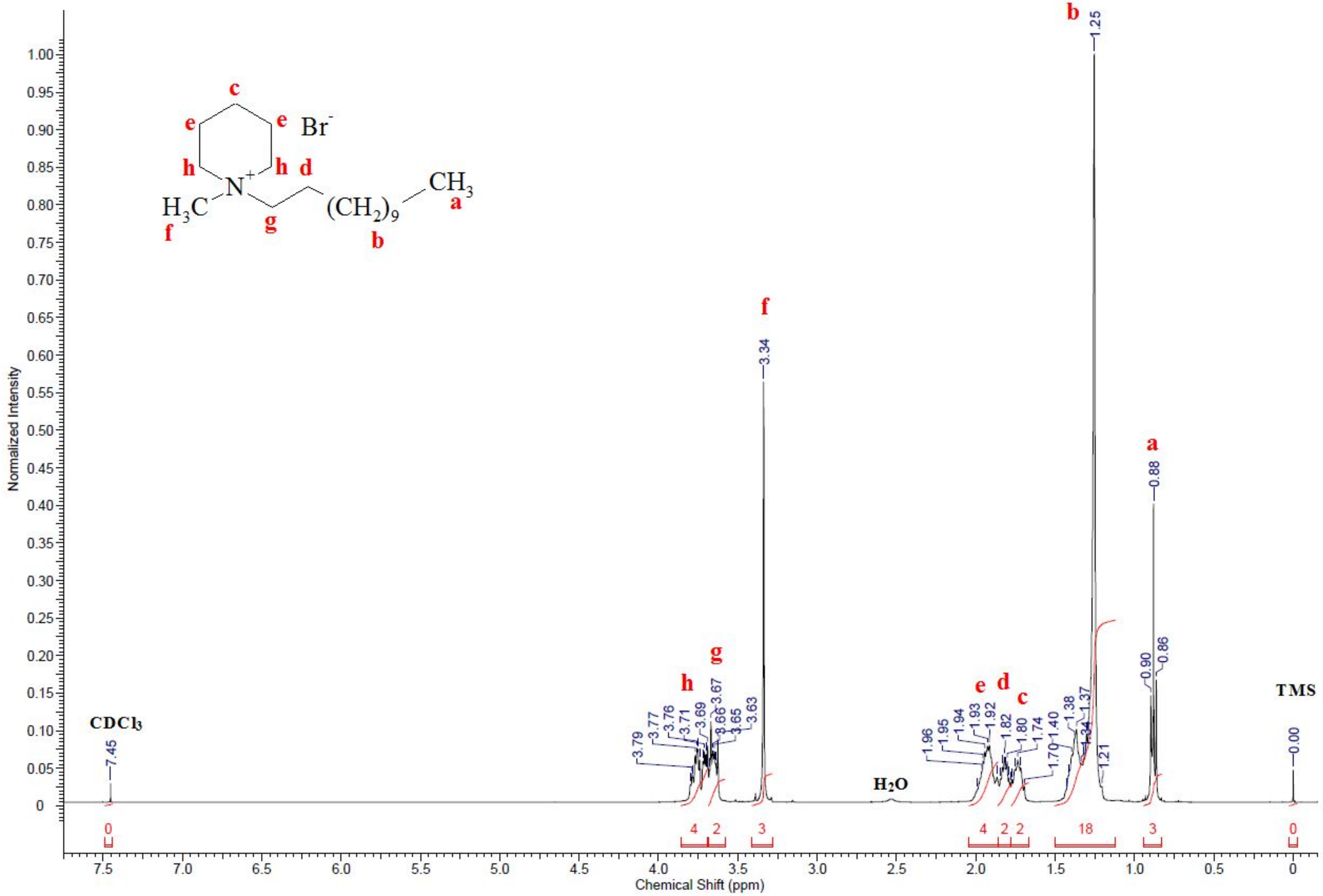


**Figure S.9.** $^{1}$H NMR spectrum of 1-dodecyl-1-methylpiperidinium bromide.

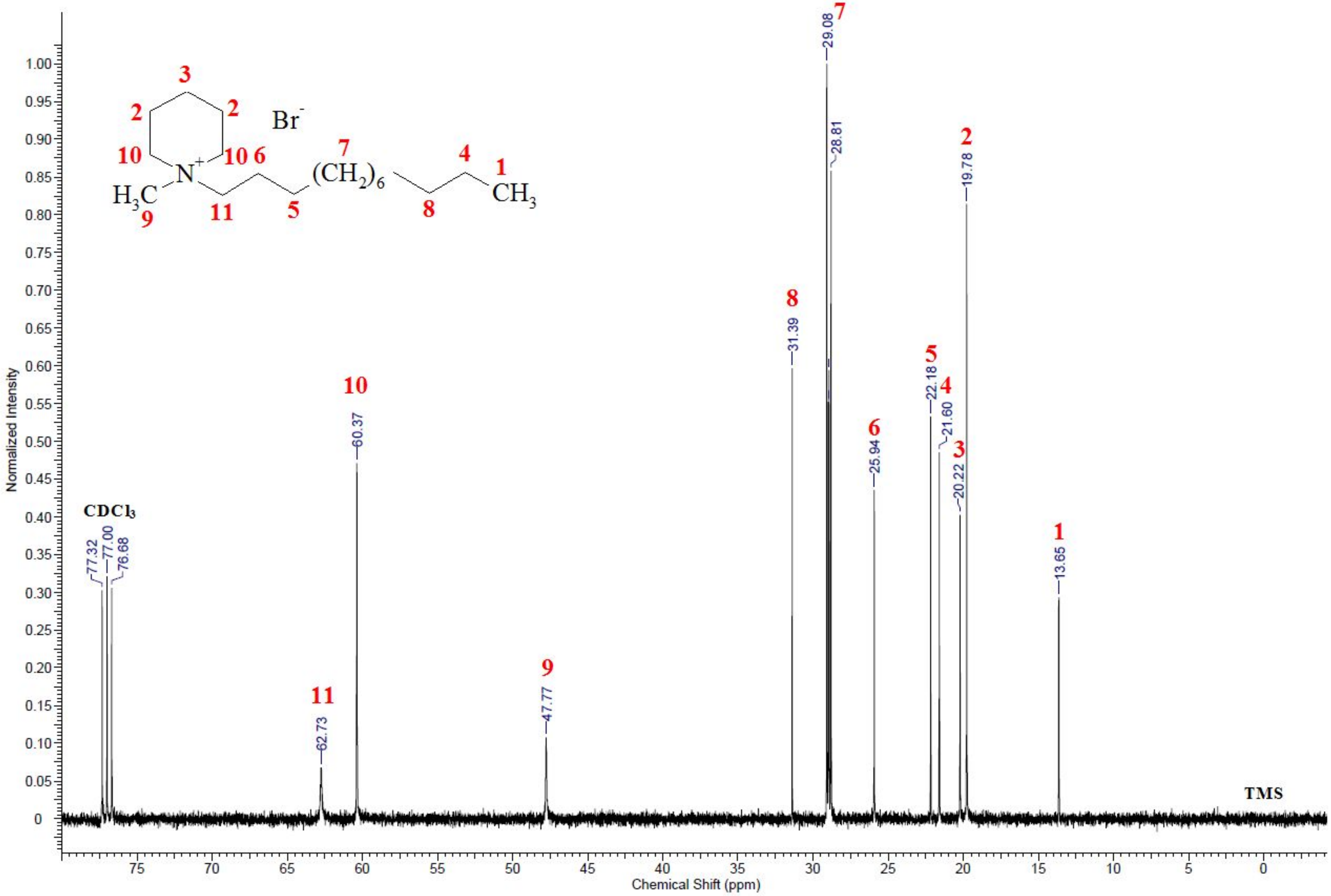


**Figure S.10.** $^{13}C$ NMR spectrum of 1-dodecyl-1-methylpiperidinium bromide.

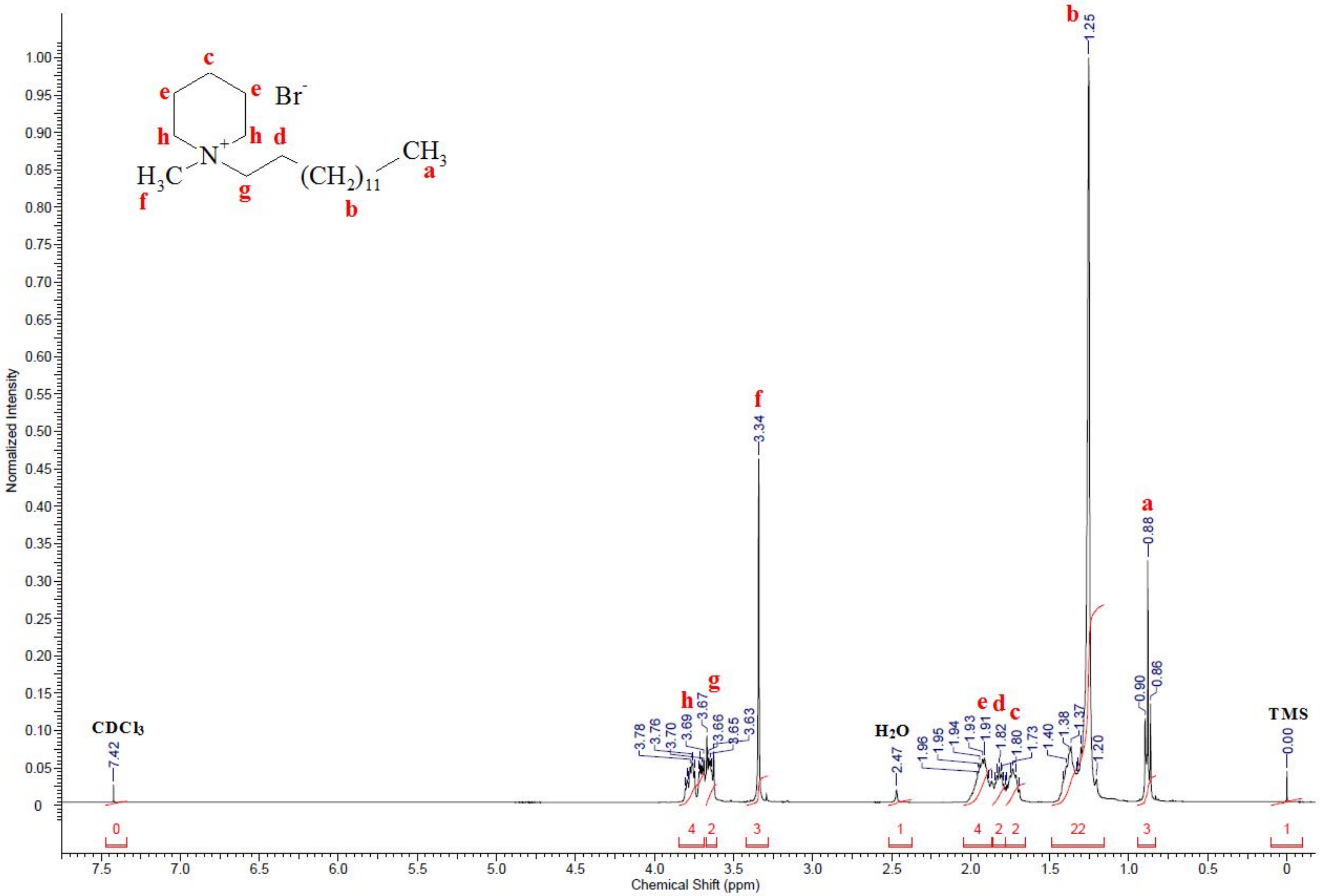


**Figure S.11.** $^1$H NMR spectrum of 1-methyl-1-tetradecylpiperidinium bromide.

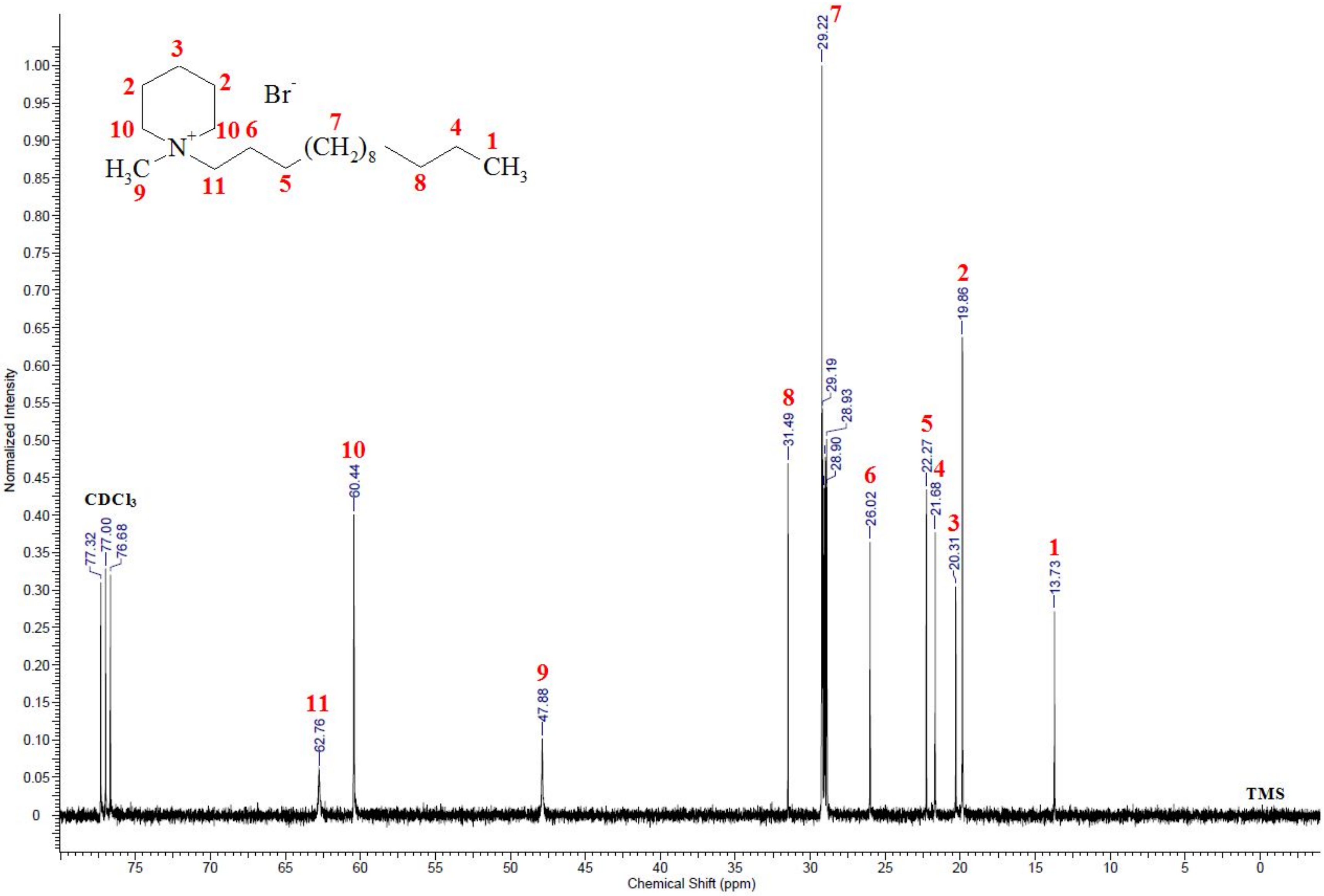


**Figure S.12.** $^{13}C$ NMR spectrum of 1-methyl-1-tetradecylpiperidinium bromide.

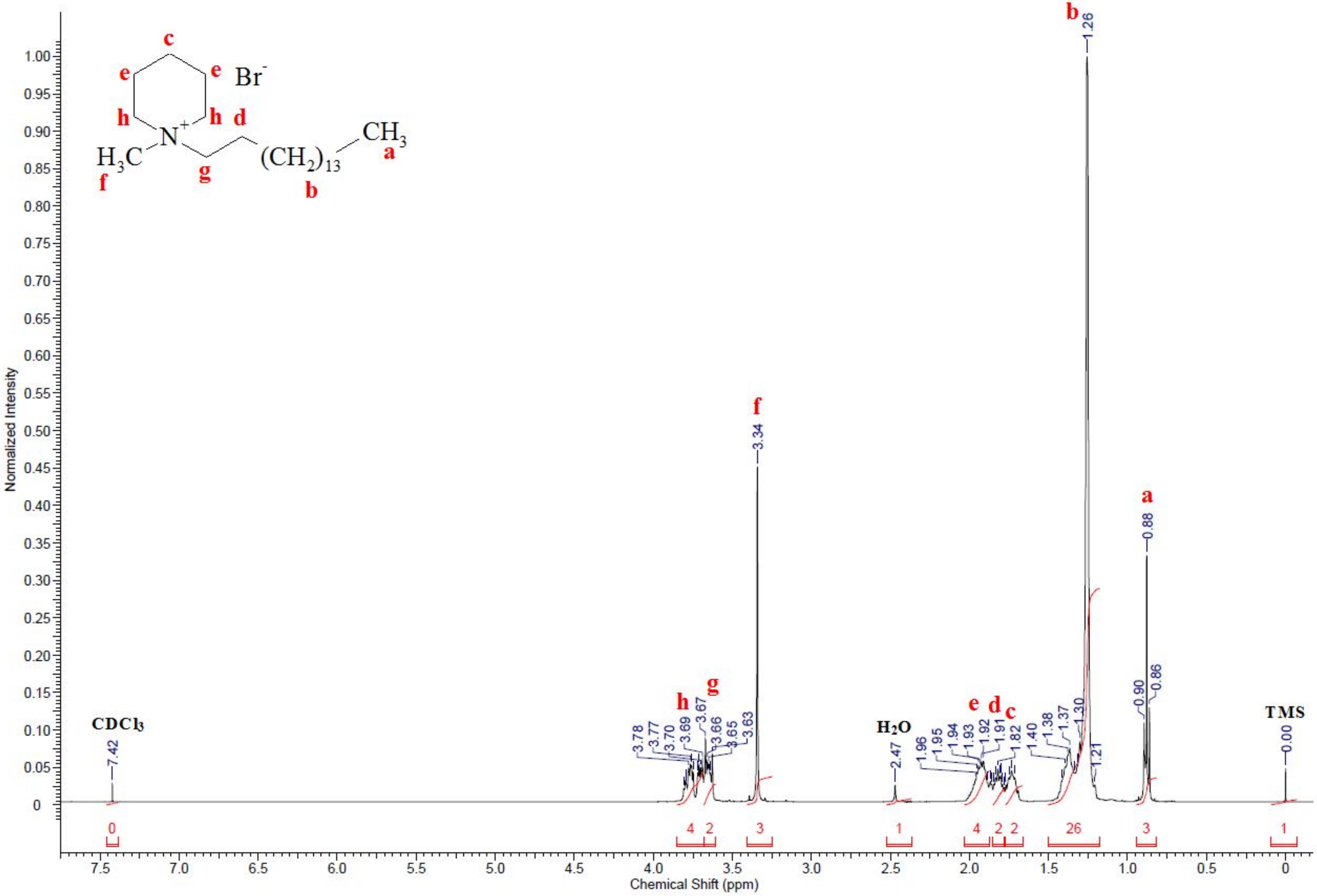


**Figure S.13.** $^1$H NMR spectrum of 1-hexadecyl-1-methylpiperidinium bromide.

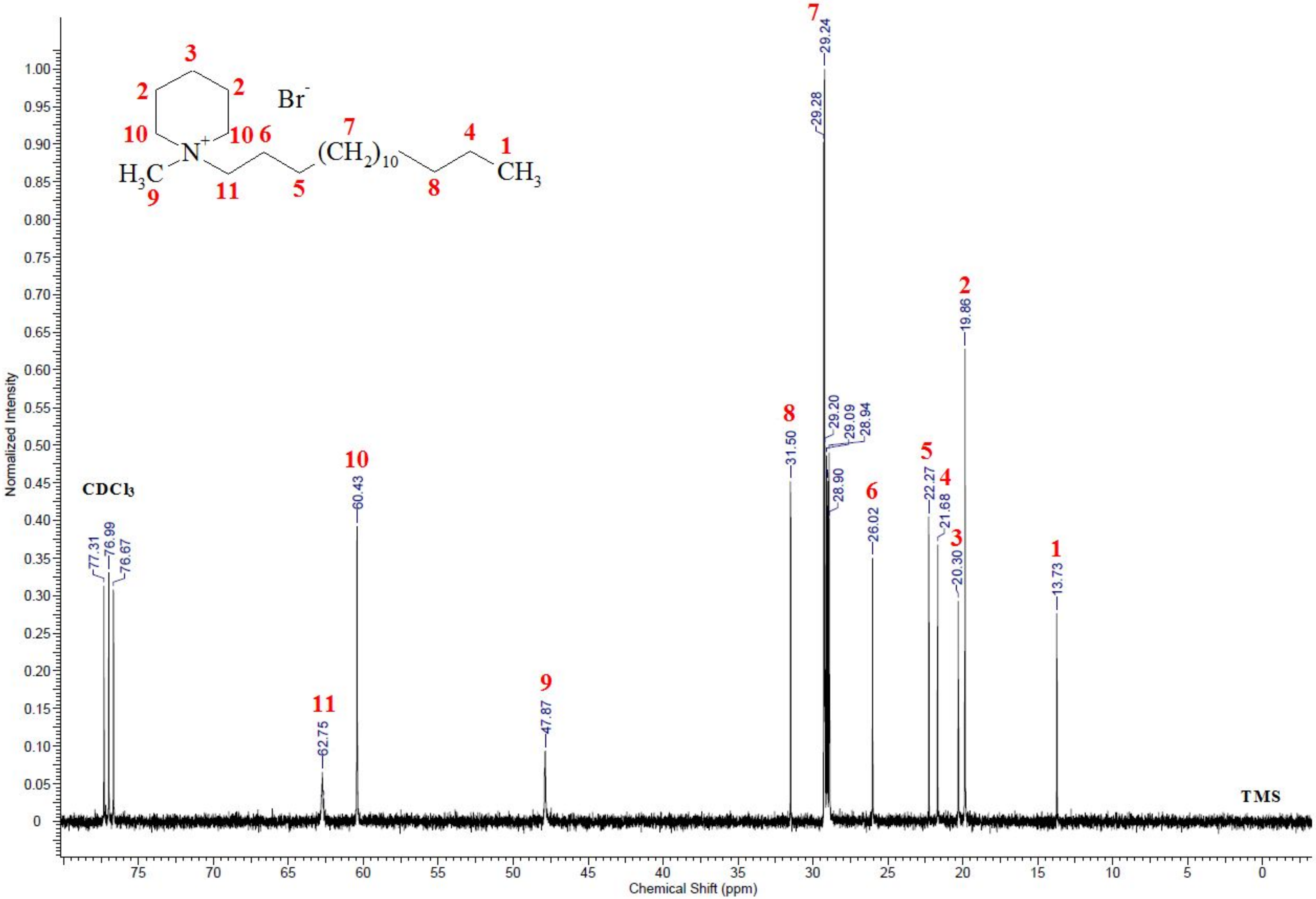

**Figure S.14.** $^{13}C$ NMR spectrum of 1-hexadecyl-1-methylpiperidinium bromide.

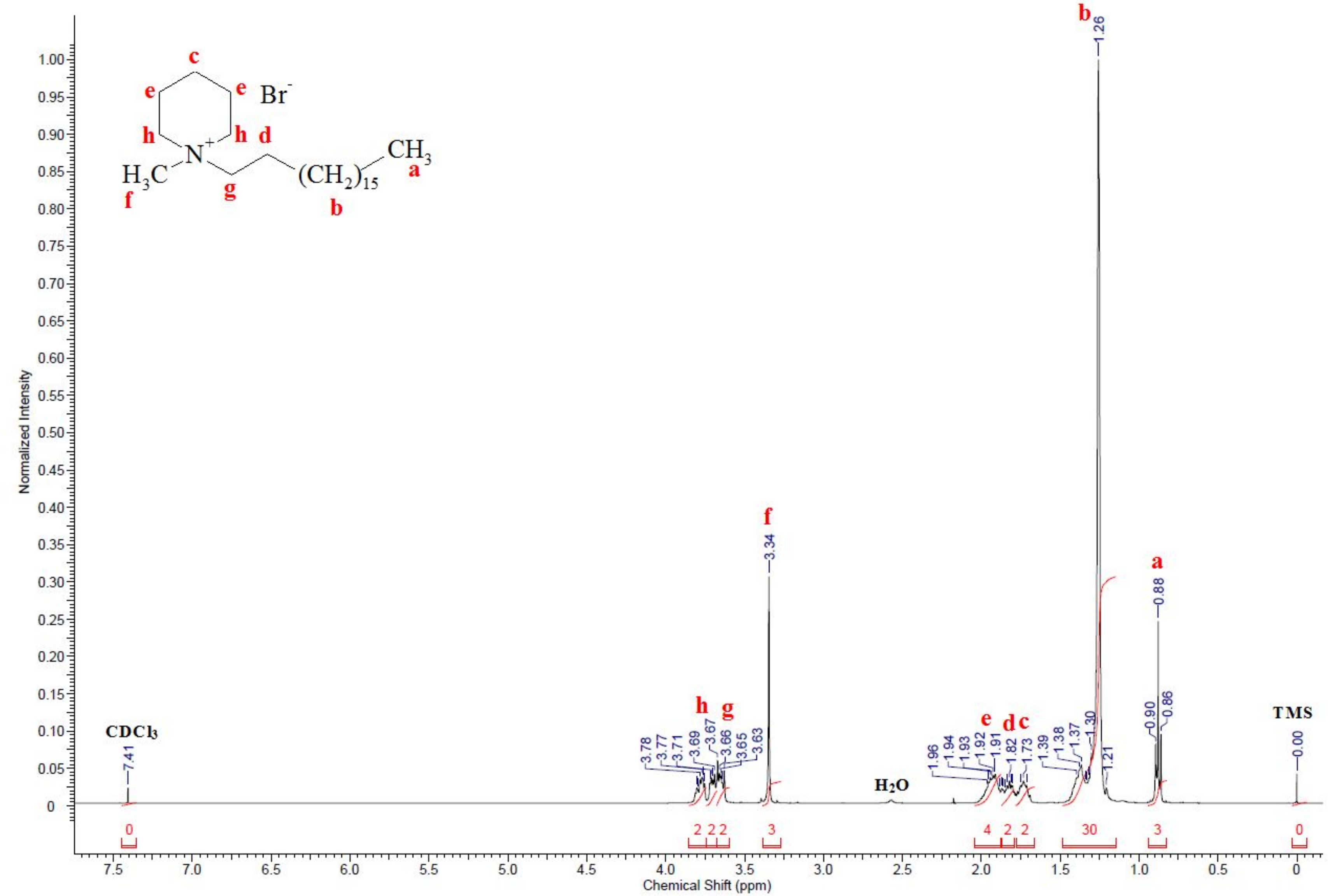


**Figure S.15.** $^{1}H$ NMR spectrum of 1-methyl-1-octadecylpiperidinium bromide.

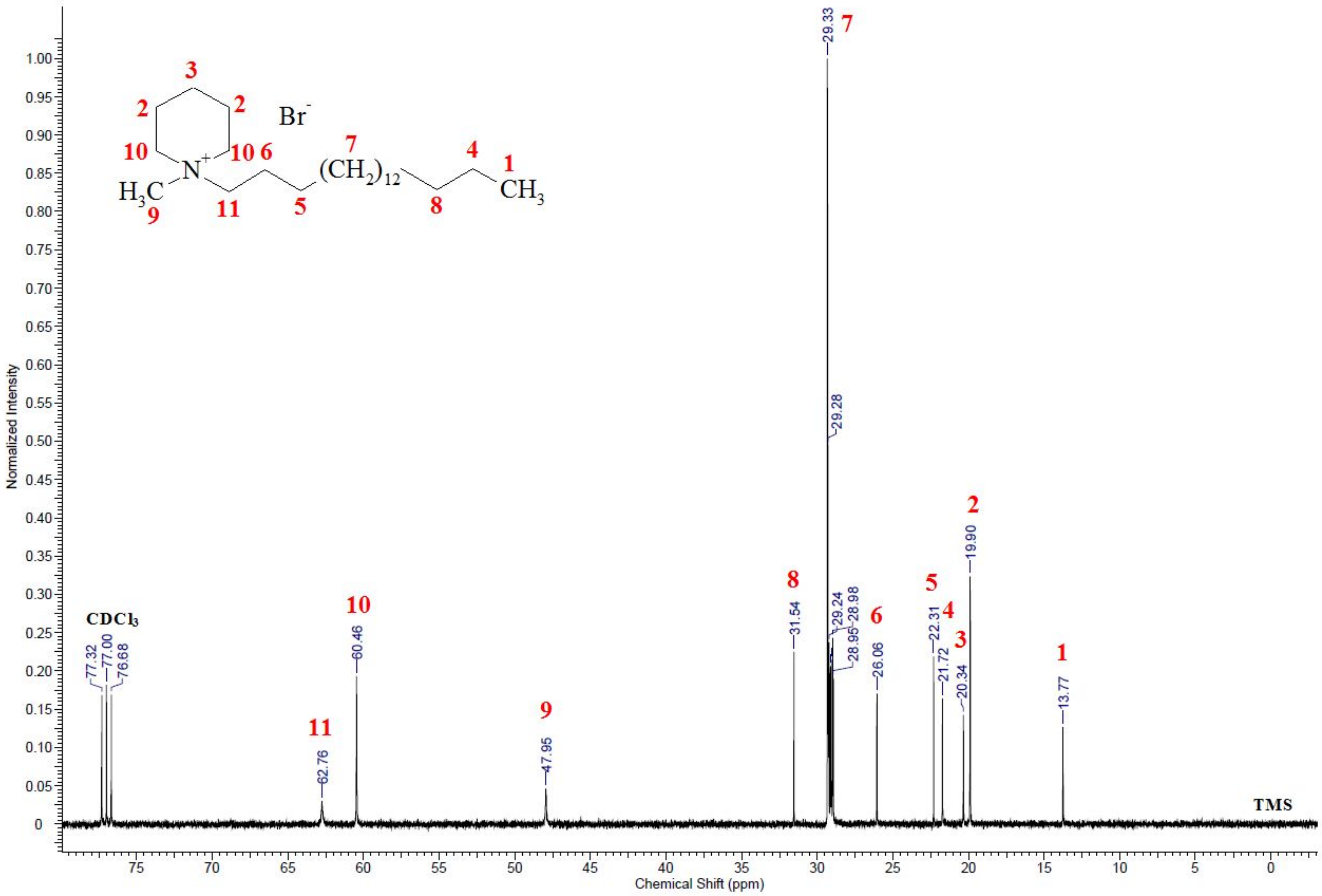


**Figure S.16.** $^{13}C$ NMR spectrum of 1-methyl-1-octadecylpiperidinium bromide.

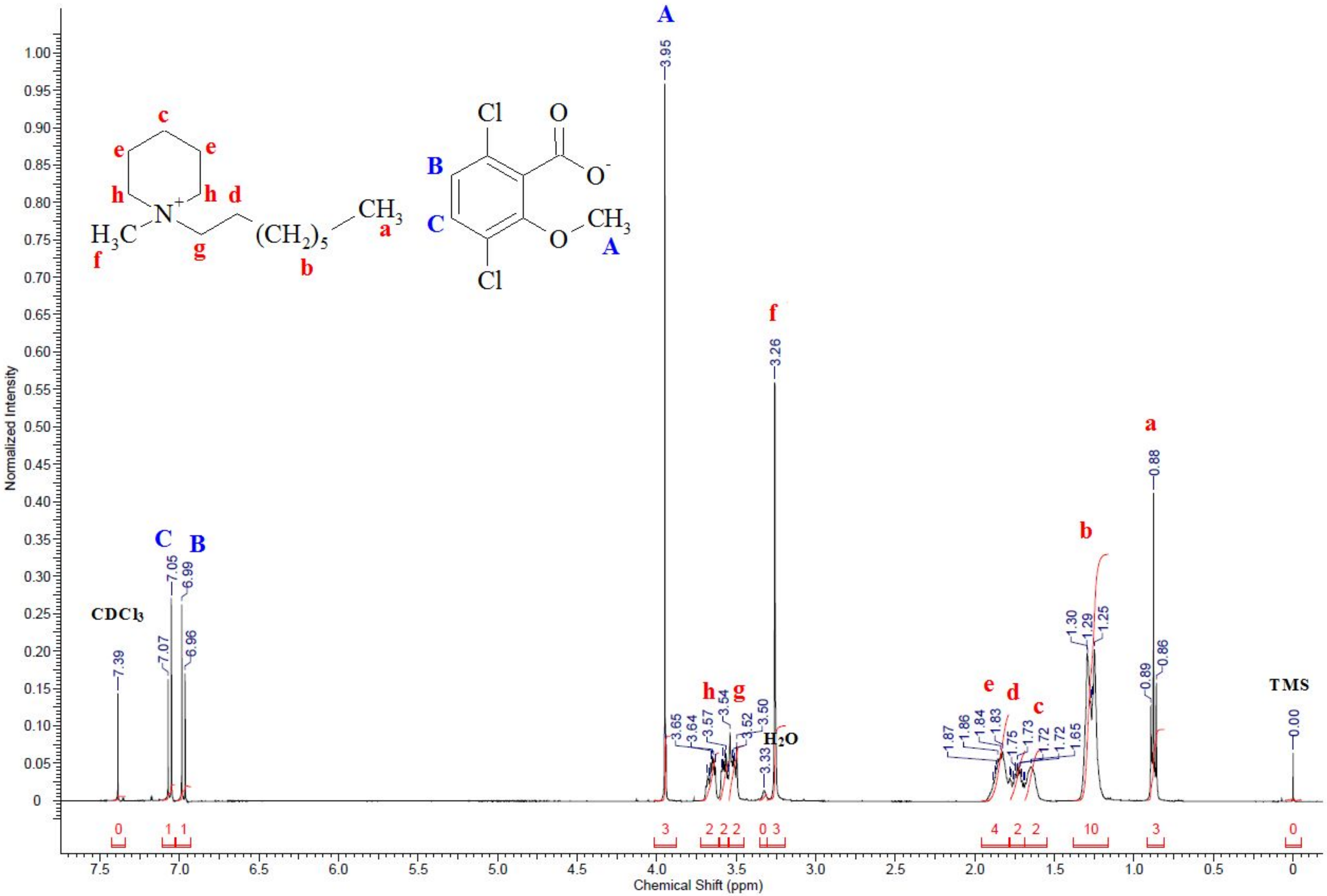


**Figure S.17.** $^{1}H$ NMR spectrum of 1-methyl-1-octylpiperidinium (3,6-dichloro-2-methoxy)benzoate.

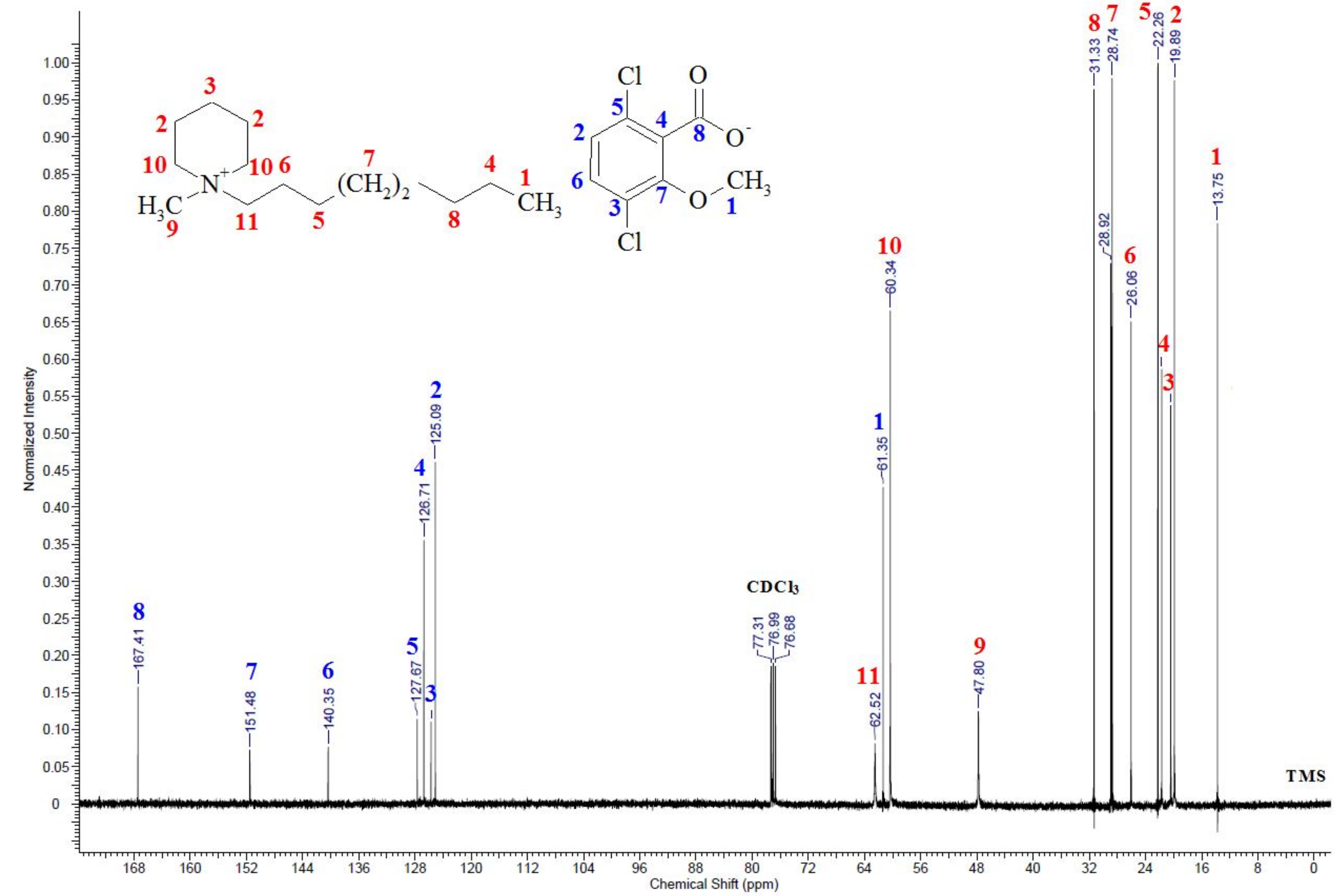


**Figure S.18.** $^{13}C$ NMR spectrum of 1-methyl-1-octylpiperidinium (3,6-dichloro-2-methoxy)benzoate.

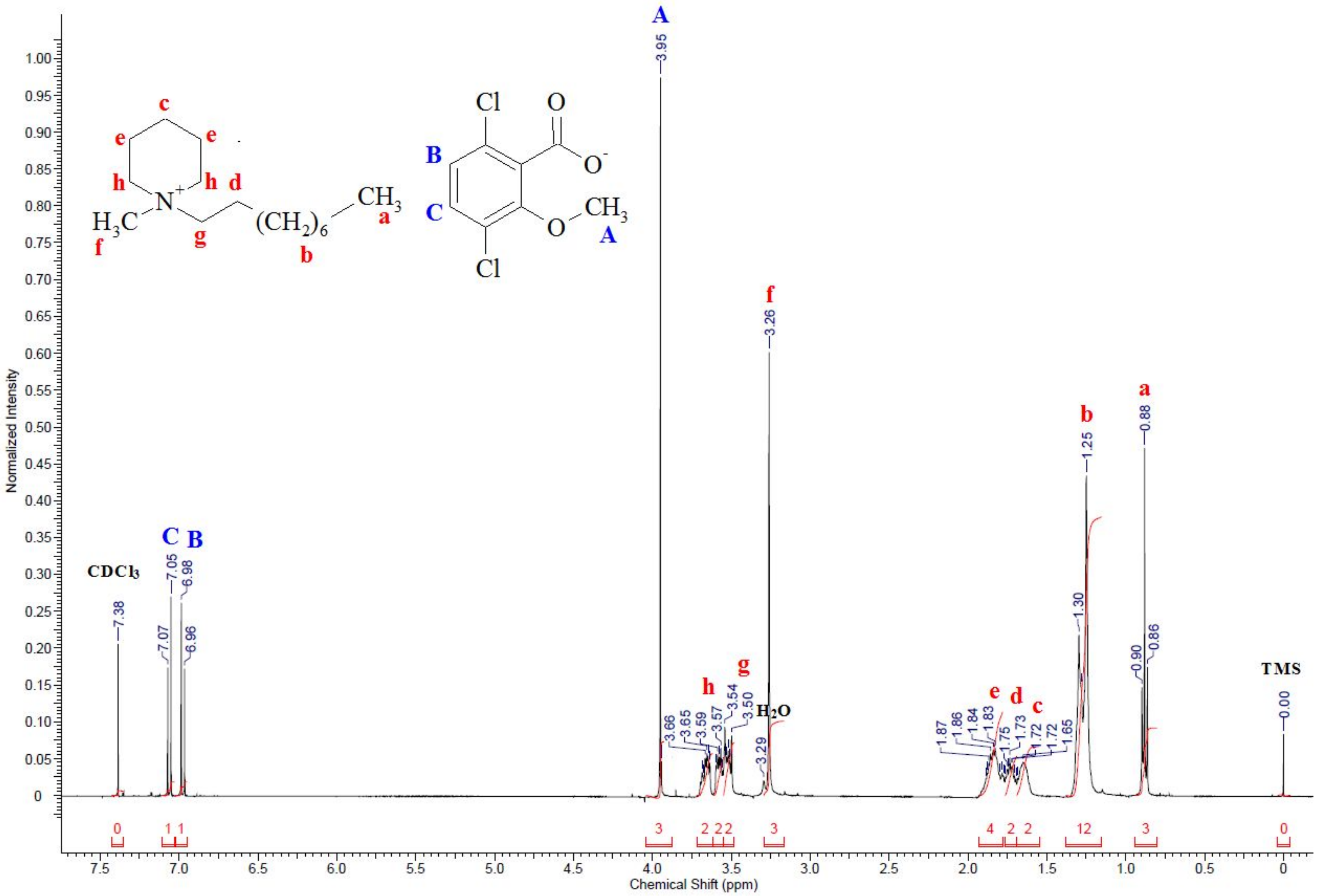


**Figure S.19.** $^{1}$H NMR spectrum of 1-methyl-1-nonylpiperidinium (3,6-dichloro-2-methoxy)benzoate.

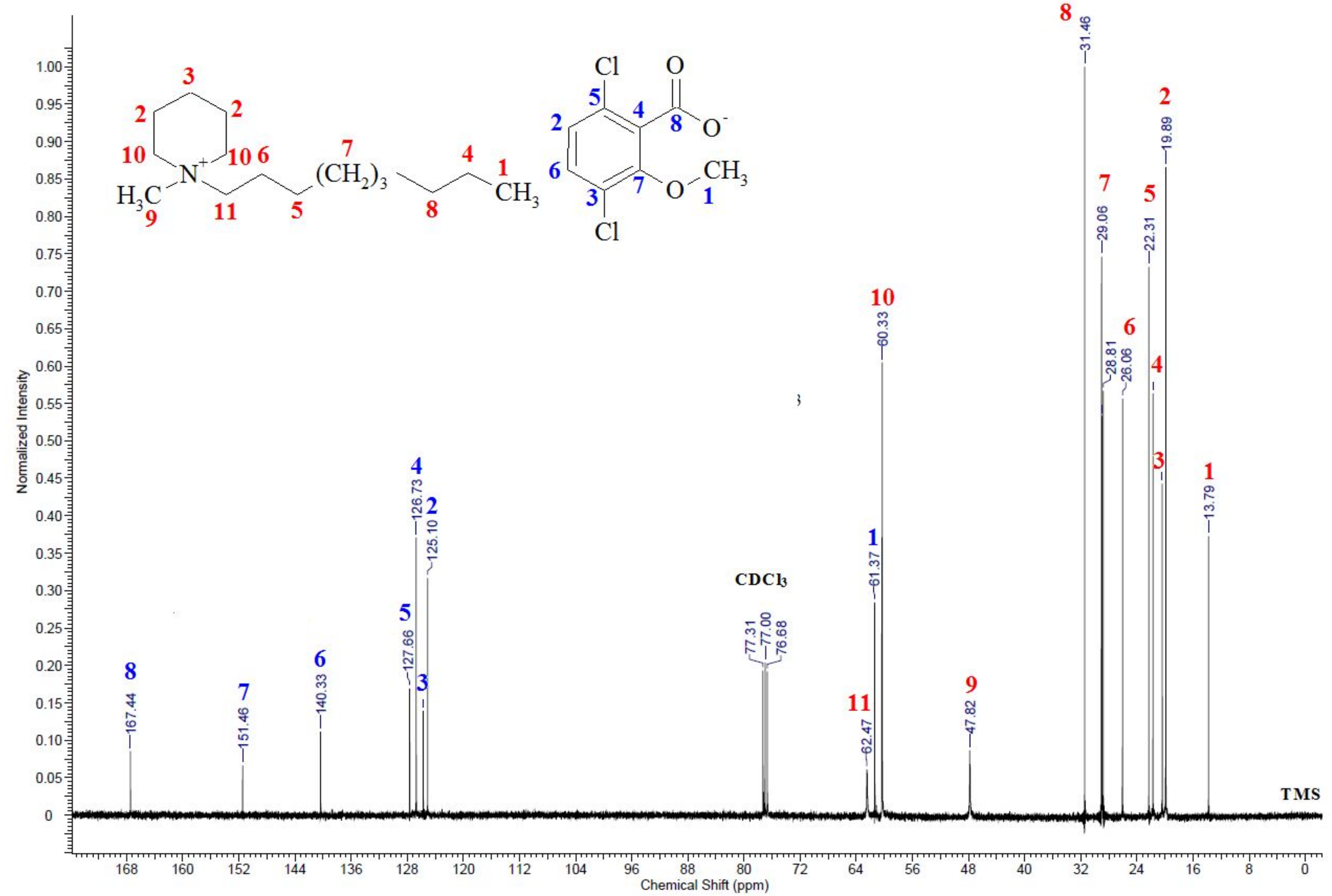


**Figure S.20.** $^{13}$C NMR spectrum of 1-methyl-1-nonylpiperidinium (3,6-dichloro-2-methoxy)benzoate.

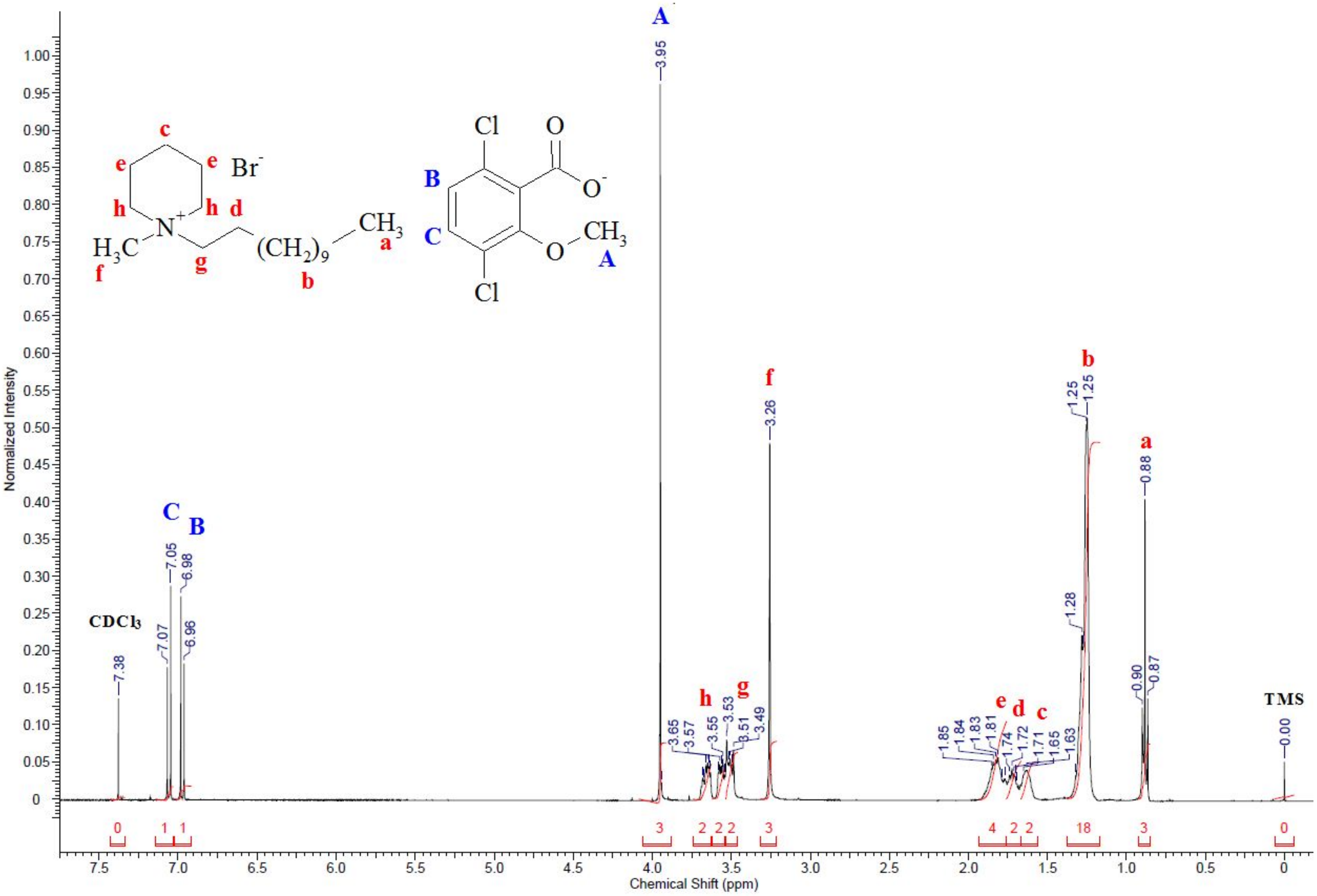


**Figure S.21.** $^1$H NMR spectrum of 1-dodecyl-1-methylpiperidinium (3,6-dichloro-2-methoxy)benzoate.

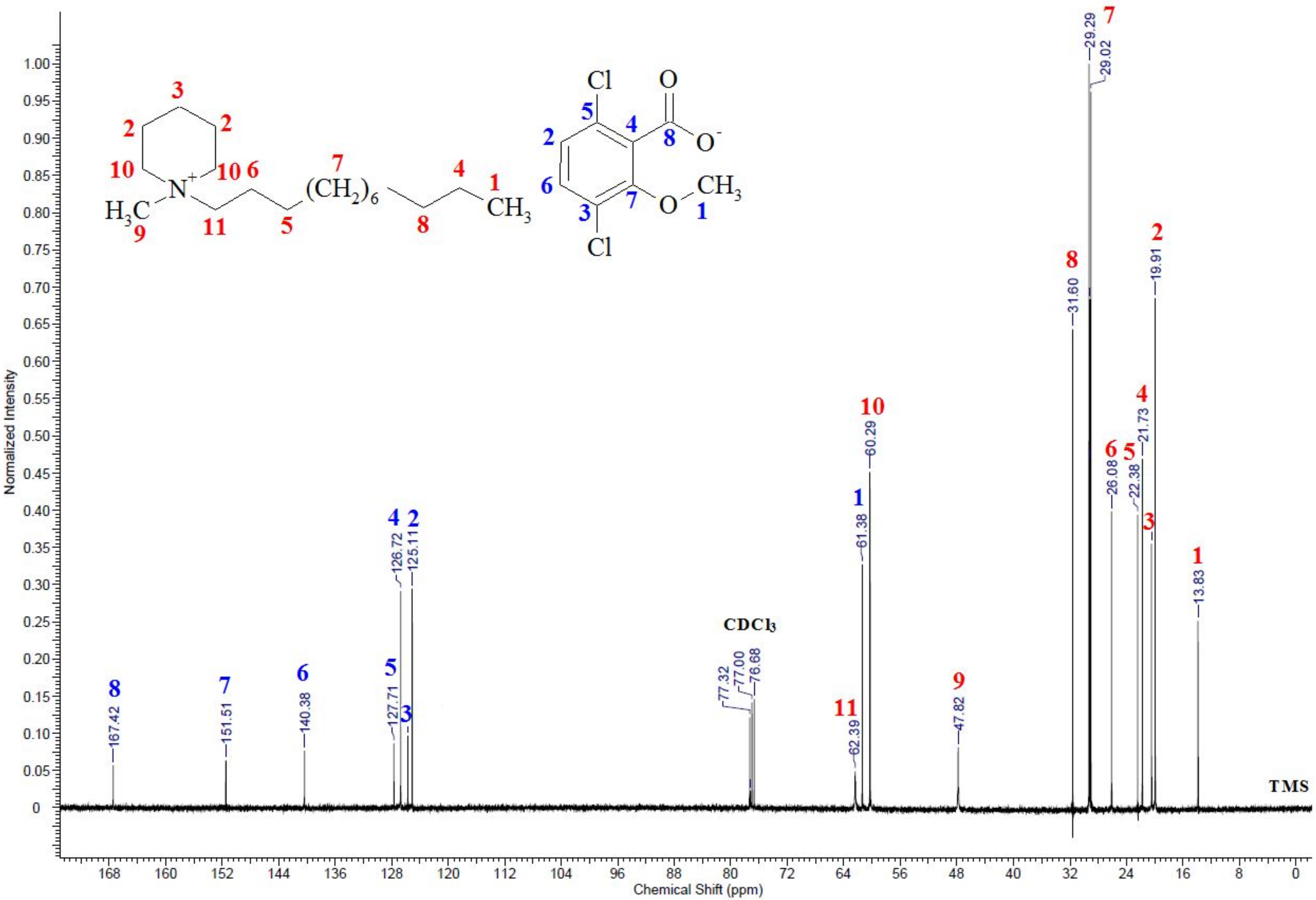


**Figure S.22.** $^{13}C$ NMR spectrum of 1-dodecyl-1-methylpiperidinium (3,6-dichloro-2-methoxy)benzoate.

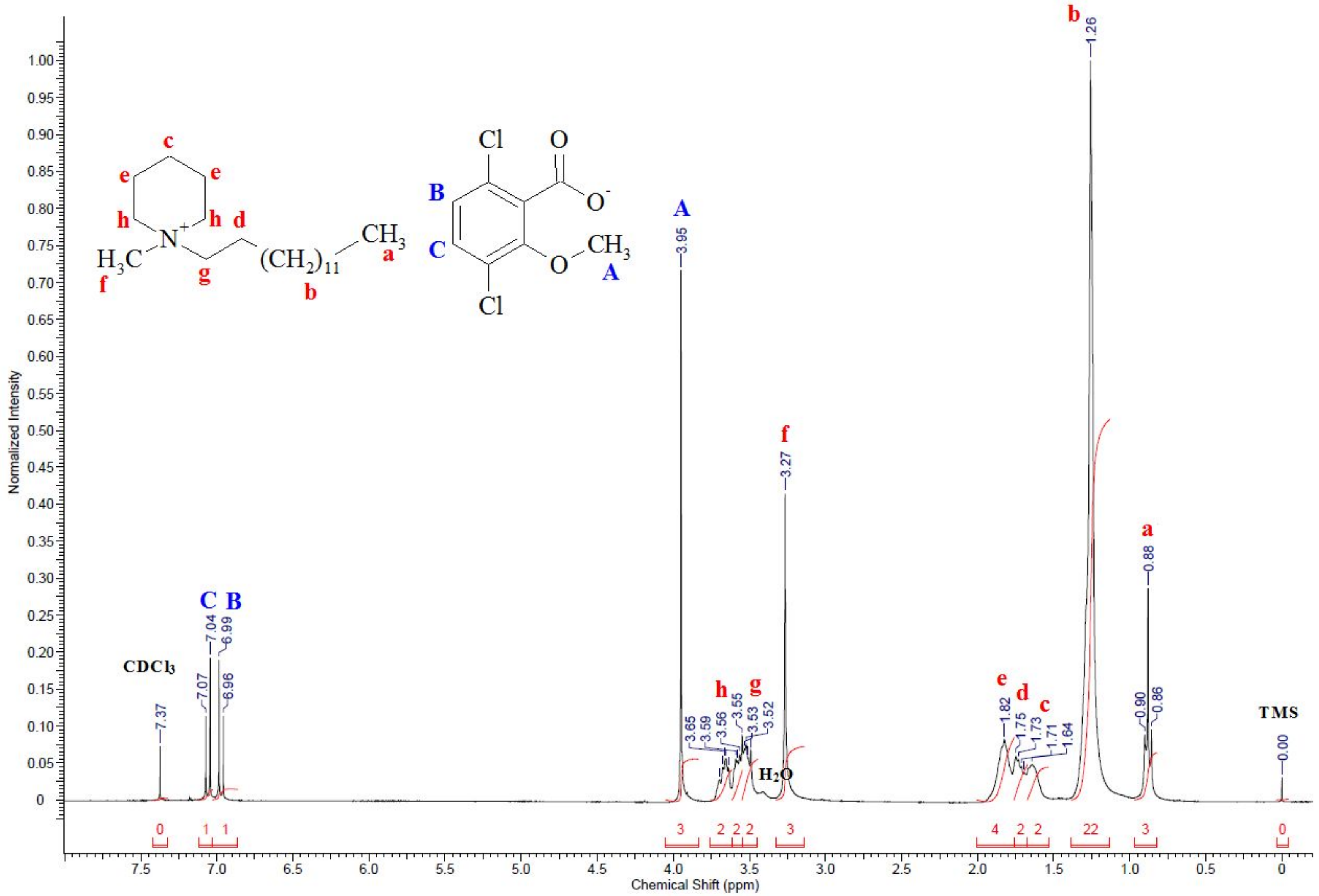


**Figure S.23.** $^1$H NMR spectrum of 1-methyl-1-tetradecylpiperidinium (3,6-dichloro-2-methoxy)benzoate.

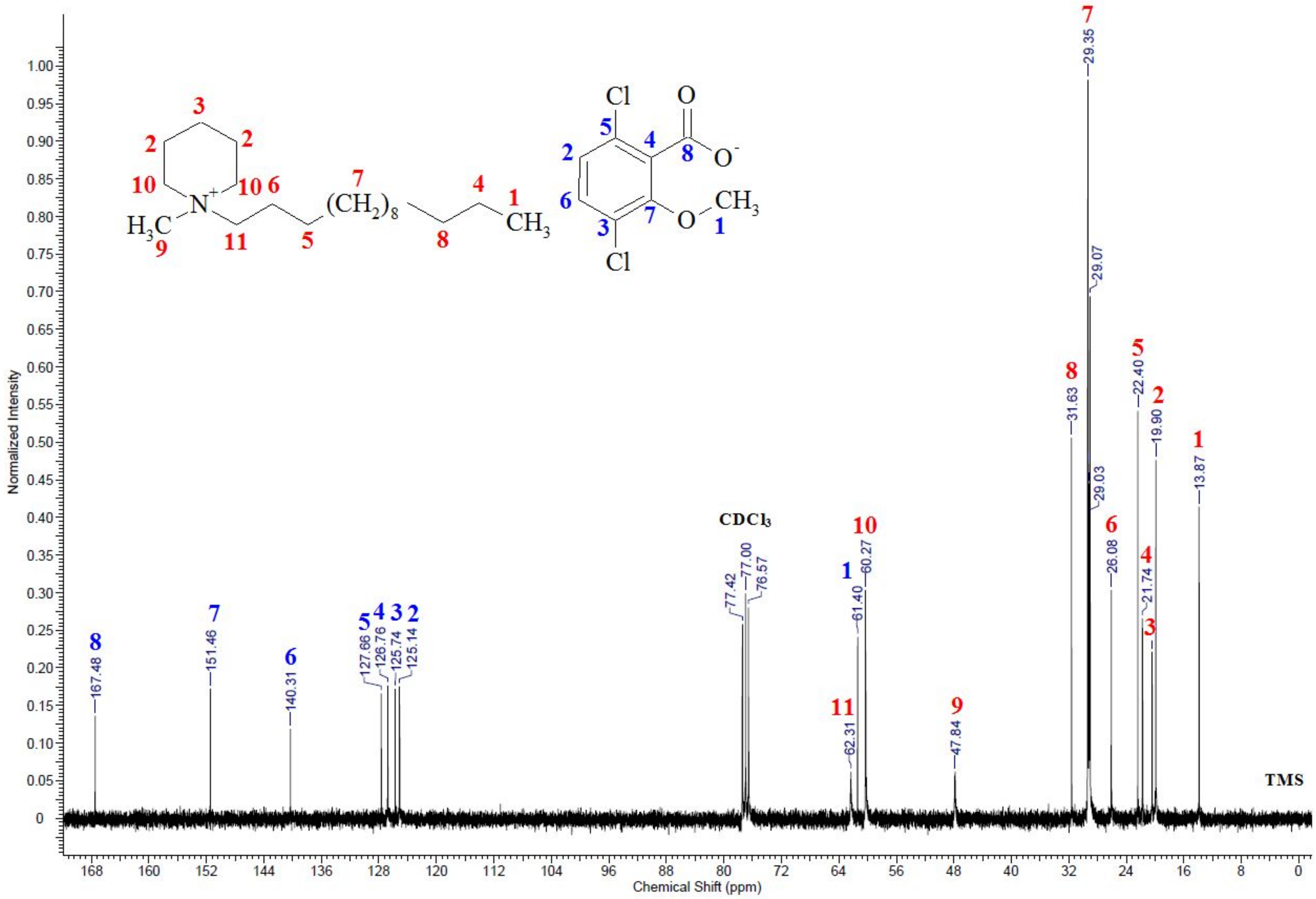


**Figure S.24.** $^{13}$C NMR spectrum of 1-methyl-1-tetradecylpiperidinium (3,6-dichloro-2-methoxy)benzoate.

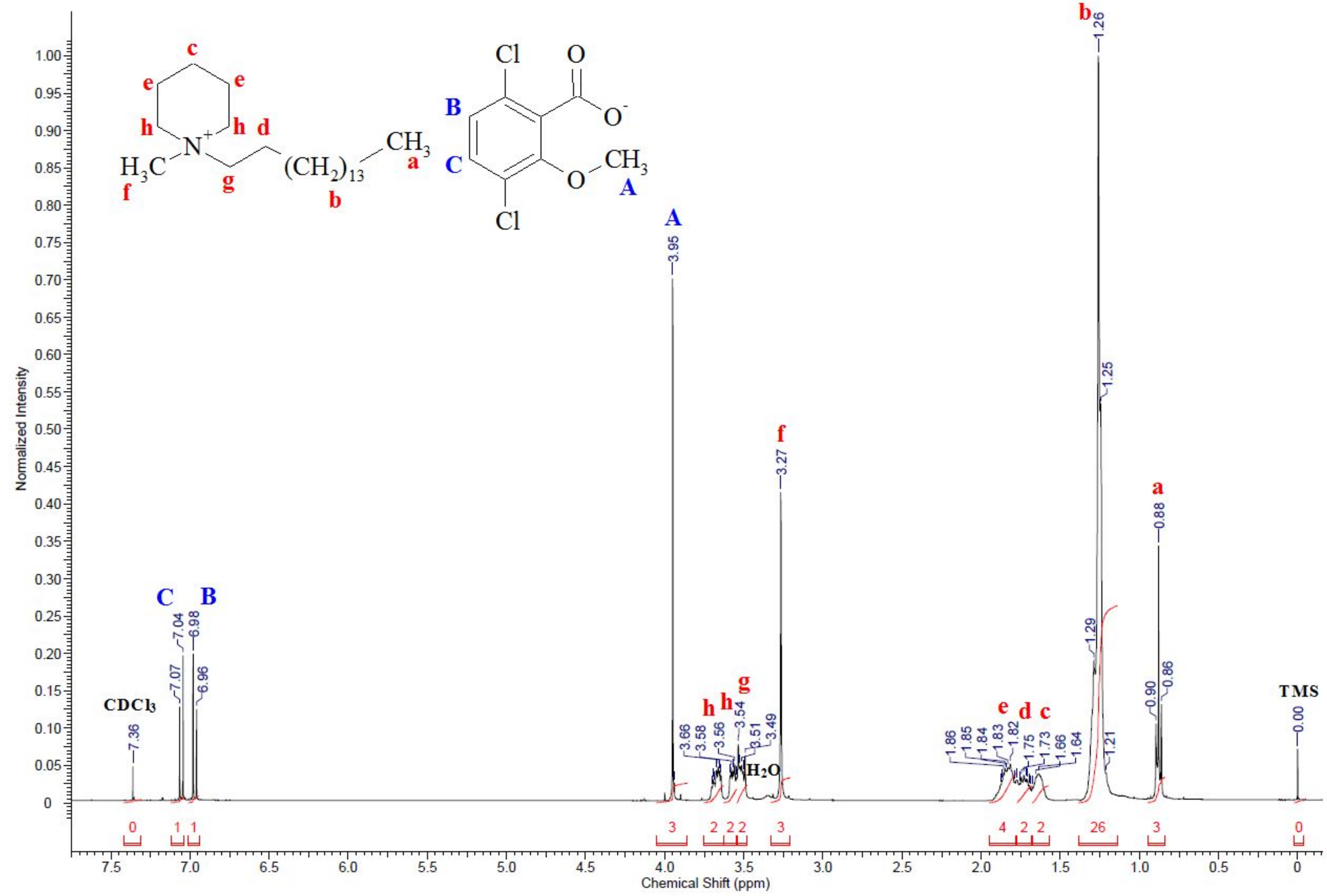


**Figure S.25.** $^{1}$H NMR spectrum of 1-hexadecyl-1-methylpiperidinium (3,6-dichloro-2-methoxy)benzoate.

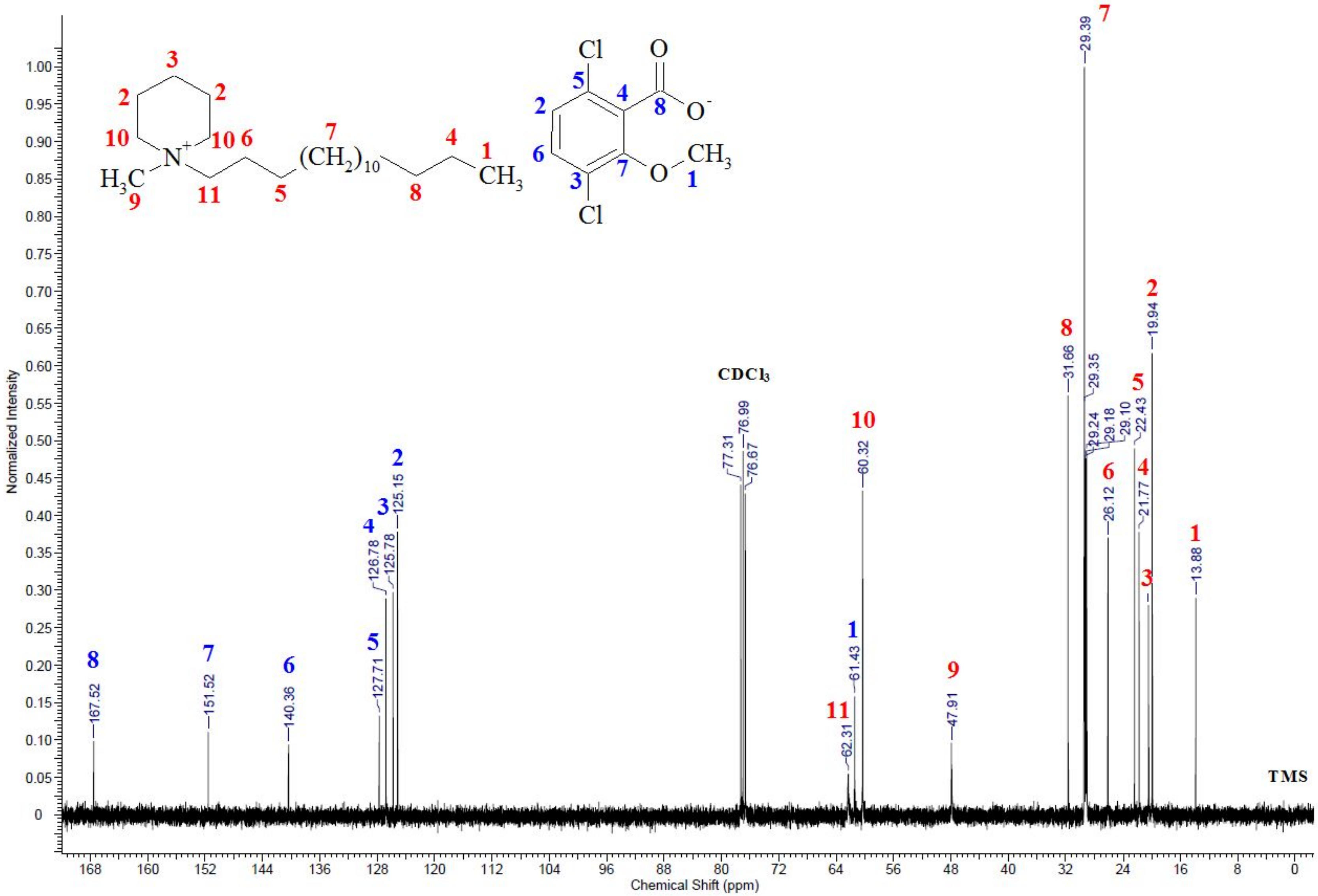


**Figure S.26.** $^{13}C$ NMR spectrum of 1-hexadecyl-1-methylpiperidinium (3,6-dichloro-2-methoxy)benzoate.

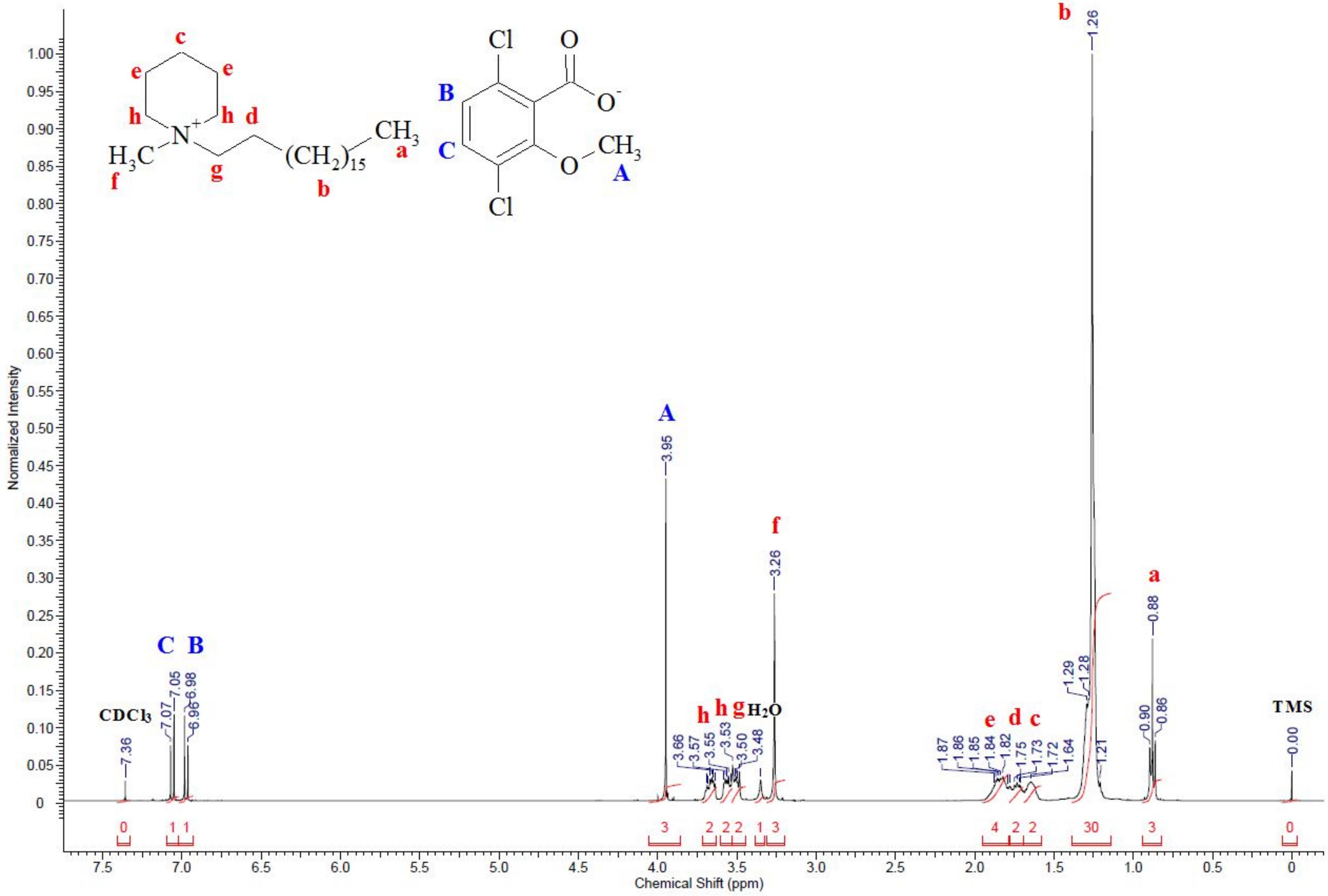


**Figure S.27.** $^{1}$H NMR spectrum of 1-methyl-1-octadecylpiperidinium (3,6-dichloro-2-methoxy)benzoate.

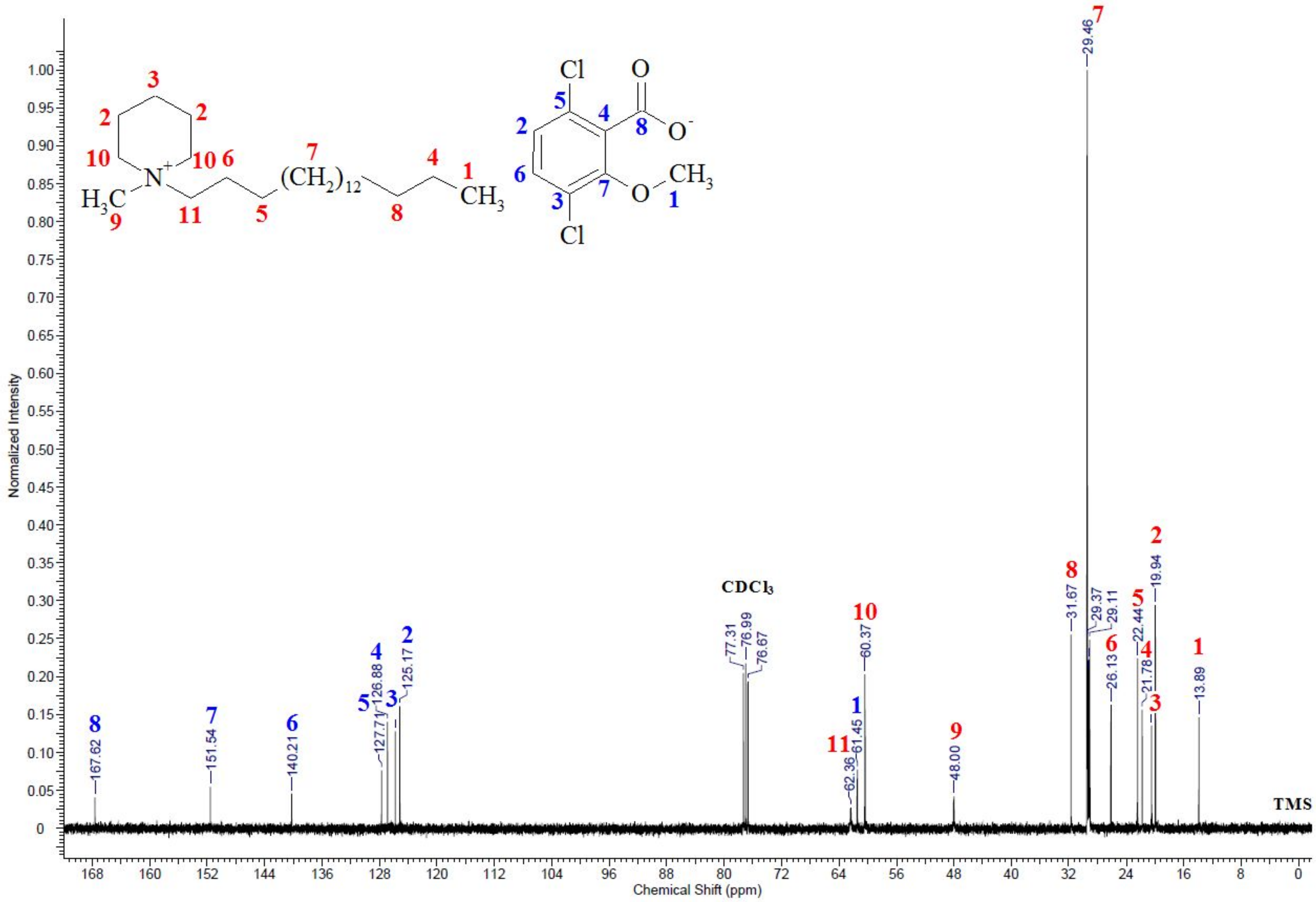


**Figure S.28.** $^{13}$C NMR spectrum of 1-methyl-1-octadecylpiperidinium (3,6-dichloro-2-methoxy)benzoate.

**Table S.2.** Chemical shifts (δ) and coupling constants (*J*) values in $^{1}$H NMR spectra for 1-methyl-1-octylpiperidinium bromide and (3,6-dichloro-2-methoxy)benzoate ($CDCl_3$)

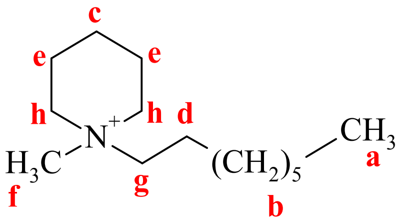


| | | | anion | | | |
|---|---|---|---|---|---|---|
| protons | number of protons | multiplicity of the signal | Br$^-$ | | 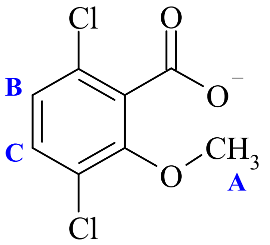  | |
| | | | δ (ppm) | *J* (Hz) | δ (ppm) | *J* (Hz) |
| **a** | 3H | triplet | 0.88 | 6.9 | 0.88 | 6.9 |
| **b** | 10H | multiplet | 1.27 | - | 1.25 | - |
| **c** | 2H | quintet | 1.74 | 7.5 | 1.65 | 7.5 |
| **d** | 2H | quintet | 1.82 | 6.0 | 1.73 | 4.6 |
| **e** | 4H | quintet | 1.93 | 5.7 | 1.84 | 4.8 |
| **f** | 3H | singlet | 3.34 | - | 3.26 | - |
| **g** | 2H | triplet | 3.65 | 8.6 | 3.52 | 8.6 |
| **h** | 4H | multiplet | 3.71, 3.76 | - | 358, 3.66 | - |
| **A** | 3H | singlet | - | - | 3.95 | - |
| **B** | 1H | doublet | - | - | 6.98 | 8.5 |
| **C** | 1H | doublet | - | - | 7.06 | 8.5 |

**Table S.3.** Chemical shift (δ) values in $^{13}C$ NMR spectra for 1-methyl-1-octylpiperidinium bromide and (3,6-dichloro-2-methoxy)benzoate ($CDCl_3$)

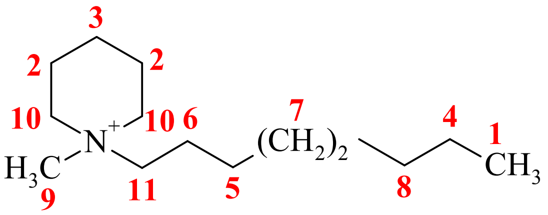

| carbon atoms | anion | |
|---|---|---|
| | $Br^-$ | 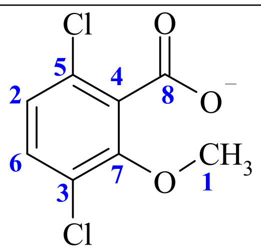 |
| | δ (ppm) | δ (ppm) |
| 1 | 13.6 | 13.8 |
| 2 | 19.8 | 19.9 |
| 3 | 20.3 | 20.4 |
| 4 | 21.6 | 21.7 |
| 5 | 22.1 | 22.3 |
| 6 | 26.0 | 26.1 |
| 7 | 28.6, 28.8 | 28.7, 28.9 |
| 8 | 31.2 | 31.3 |
| 9 | 47.8 | 47.8 |
| 10 | 60.4 | 60.3 |
| 1 | - | 61.4 |
| 11 | 62.9 | 62.5 |
| 2 | - | 125.1 |
| 3 | - | 125.7 |
| 4 | - | 126.7 |
| 5 | - | 127.7 |
| 6 | - | 140.4 |
| 7 | - | 151.5 |
| 8 | - | 167.4 |

**Table S.4.** Chemical shifts (δ) and coupling constants (*J*) values in $^{1}H$ NMR spectra for 1-methyl-1-nonylpiperidinium bromide and (3,6-dichloro-2-methoxy)benzoate ($CDCl_3$)

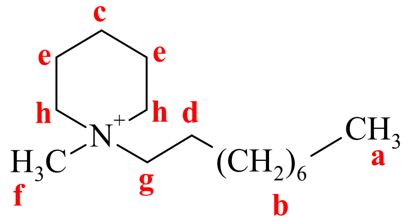


| protons | number of protons | multiplicity of the signal | anion | | | |
|---|---|---|---|---|---|---|
| | | | $Br^-$ | | 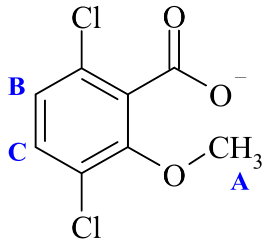  | |
| | | | δ (ppm) | *J* (Hz) | δ (ppm) | *J* (Hz) |
| **a** | 3H | triplet | 0.88 | 6.9 | 0.88 | 6.9 |
| **b** | 12H | multiplet | 1.26 | - | 1.25 | - |
| **c** | 2H | quintet | 1.74 | 7.5 | 1.65 | 7.4 |
| **d** | 2H | quintet | 1.82 | 5.5 | 1.72 | 4.6 |
| **e** | 4H | quintet | 1.93 | 5.5 | 1.84 | 4.9 |
| **f** | 3H | singlet | 3.34 | - | 3.26 | - |
| **g** | 2H | triplet | 3.65 | 8.6 | 3.52 | 8.6 |
| **h** | 4H | multiplet | 3.70, 3.76 | - | 3.58, 3.66 | - |
| **A** | 3H | singlet | - | - | 3.95 | - |
| **B** | 1H | doublet | - | - | 6.97 | 8.6 |
| **C** | 1H | doublet | - | - | 7.06 | 8.5 |

**Table S.5.** Chemical shift (δ) values in $^{13}C$ NMR spectra for 1-methyl-1-nonylpiperidinium bromide and (3,6-dichloro-2-methoxy)benzoate ($CDCl_3$)

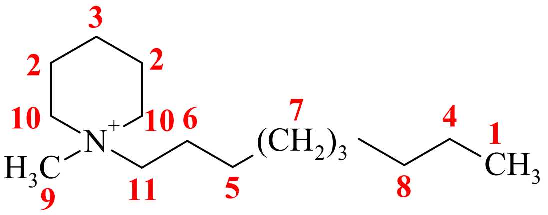


| carbon atoms | anion | |
|---|---|---|
| | Br⁻ | 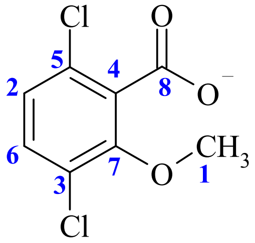 |
| | δ (ppm) | δ (ppm) |
| 1 | 13.6 | 13.8 |
| 2 | 19.7 | 19.9 |
| 3 | 20.2 | 20.4 |
| 4 | 21.5 | 21.7 |
| 5 | 22.0 | 22.3 |
| 6 | 25.9 | 26.1 |
| 7 | 28.6, 28.7, 28.8 | 28.8, 29.0, 29.1 |
| 8 | 31.2 | 31.5 |
| 9 | 47.7 | 47.8 |
| 10 | 60.3 | 60.3 |
| 1 | - | 61.4 |
| 11 | 62.7 | 62.5 |
| 2 | - | 125.1 |
| 3 | - | 125.7 |
| 4 | - | 126.7 |
| 5 | - | 127.7 |
| 6 | - | 140.3 |
| 7 | - | 151.5 |
| 8 | - | 167.4 |

**Table S.6.** Chemical shifts (δ) and coupling constants (*J*) values in $^{1}$H NMR spectra for 1-dodecyl-1-methylpiperidinium bromide and (3,6-dichloro-2-methoxy)benzoate ($CDCl_3$)

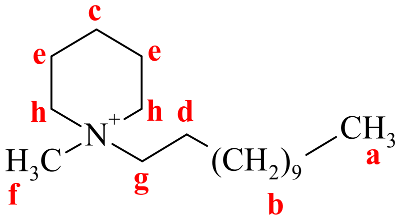


| protons | number of protons | multiplicity of the signal | anion: $Br^-$ | | anion: 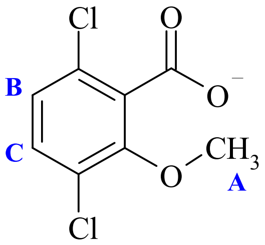 | |
|---|---|---|---|---|---|---|
| | | | δ (ppm) | *J* (Hz) | δ (ppm) | *J* (Hz) |
| a | 3H | triplet | 0.88 | 6.9 | 0.88 | 6.9 |
| b | 18H | multiplet | 1.25 | - | 1.25 | - |
| c | 2H | quintet | 1.74 | 7.7 | 1.63 | 7.5 |
| d | 2H | quintet | 1.82 | 5.3 | 1.71 | 4.8 |
| e | 4H | quintet | 1.93 | 5.8 | 1.83 | 4.8 |
| f | 3H | singlet | 3.34 | - | 3.26 | - |
| g | 2H | triplet | 3.65 | 8.6 | 3.51 | 8.6 |
| h | 4H | multiplet | 3.70, 3.77 | - | 3.56, 3.66 | - |
| A | 3H | singlet | - | - | 3.95 | - |
| B | 1H | doublet | - | - | 6.97 | 8.5 |
| C | 1H | doublet | - | - | 7.06 | 8.5 |

**Table S.7.** Chemical shift (δ) values in $^{13}C$ NMR spectra for 1-dodecyl-1-methylpiperidinium bromide and (3,6-dichloro-2-methoxy)benzoate ($CDCl_3$)

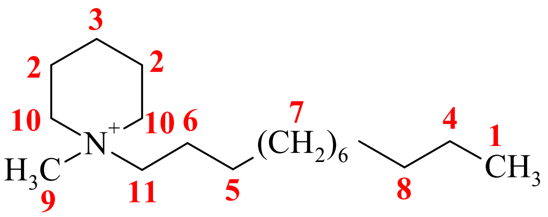


| carbon atoms | anion | |
|---|---|---|
| | Br⁻ | 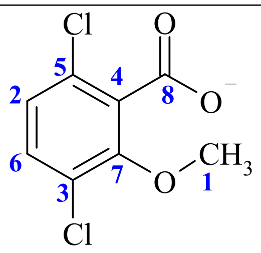  |
| | δ (ppm) | δ (ppm) |
| 1 | 13.7 | 13.8 |
| 2 | 19.8 | 19.9 |
| 3 | 20.2 | 20.4 |
| 4 | 21.6 | 21.7 |
| 5 | 22.2 | 22.4 |
| 6 | 25.9 | 26.1 |
| 7 | 28.6, 28.9, 29.0, 29.1 | 29.0, 29.1, 29.2, 29.3 |
| 8 | 31.4 | 31.6 |
| 9 | 47.8 | 47.8 |
| 10 | 60.4 | 60.3 |
| 1 | - | 61.4 |
| 11 | 62.7 | 62.4 |
| 2 | - | 125.1 |
| 3 | - | 125.7 |
| 4 | - | 126.7 |
| 5 | - | 127.7 |
| 6 | - | 140.4 |
| 7 | - | 151.5 |
| 8 | - | 167.4 |

**Table S.8.** Chemical shifts (δ) and coupling constants (*J*) values in $^{1}$H NMR spectra for 1-methyl-1-tetradecylpiperidinium bromide and (3,6-dichloro-2-methoxy)benzoate ($CDCl_3$)

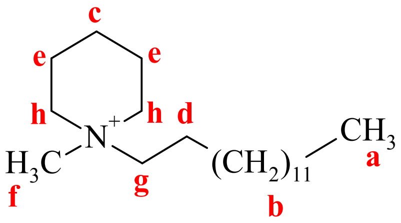


| protons | number of protons | multiplicity of the signal | anion | | | |
|---|---|---|---|---|---|---|
| | | | $Br^-$ | | 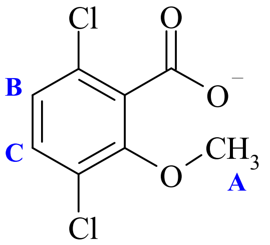 | |
| | | | δ (ppm) | *J* (Hz) | δ (ppm) | *J* (Hz) |
| **a** | 3H | triplet | 0.88 | 6.9 | 0.88 | 6.7 |
| **b** | 22H | multiplet | 1.25 | - | 1.26 | - |
| **c** | 2H | quintet | 1.73 | 7.4 | 1.64 | 7.4 |
| **d** | 2H | quintet | 1.82 | 5.5 | 1.72 | 5.4 |
| **e** | 4H | quintet | 1.93 | 5.0 | 1.82 | 5.0 |
| **f** | 3H | singlet | 3.34 | - | 3.27 | - |
| **g** | 2H | triplet | 3.65 | 8.6 | 3.52 | 8.6 |
| **h** | 4H | multiplet | 3.70, 3.78 | - | 3.65, 3.58 | - |
| **A** | 3H | singlet | - | - | 3.95 | - |
| **B** | 1H | doublet | - | - | 6.97 | 8.7 |
| **C** | 1H | doublet | - | - | 7.06 | 8.4 |

**Table S.9.** Chemical shift (δ) values in $^{13}C$ NMR spectra for 1-methyl-1-tetradecylpiperidinium bromide and (3,6-dichloro-2-methoxy)benzoate ($CDCl_3$)

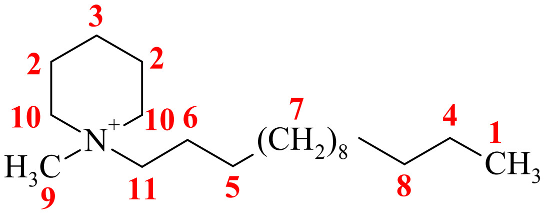


| carbon atoms | anion | |
|---|---|---|
| | $Br^-$ | 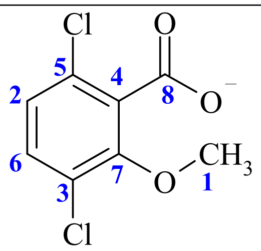  |
| | δ (ppm) | δ (ppm) |
| 1 | 13.7 | 13.9 |
| 2 | 19.9 | 19.9 |
| 3 | 20.3 | 20.4 |
| 4 | 21.7 | 21.7 |
| 5 | 22.3 | 22.4 |
| 6 | 26.0 | 26.1 |
| 7 | 28.90, 28.93, 29.02, 29.08, 29.19, 29.22, 29.25 | 29.03, 29.07, 29.14, 29.21, 29.31, 29.35, 29.38 |
| 8 | 31.5 | 31.6 |
| 9 | 47.9 | 47.8 |
| 10 | 60.4 | 60.3 |
| 1 | - | 61.4 |
| 11 | 62.8 | 62.3 |
| 2 | - | 125.1 |
| 3 | - | 125.7 |
| 4 | - | 126.8 |
| 5 | - | 127.7 |
| 6 | - | 140.3 |
| 7 | - | 151.5 |
| 8 | - | 167.5 |

**Table S.10.** Chemical shifts (δ) and coupling constants (*J*) values in $^{1}$H NMR spectra for 1-hexadecyl-1-methylpiperidinium bromide and (3,6-dichloro-2-methoxy)benzoate ($CDCl_3$)

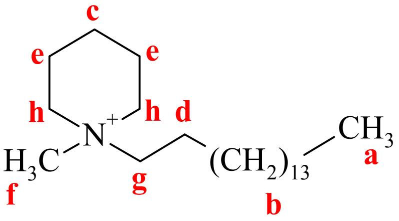


| protons | number of protons | multiplicity of the signal | anion | | | |
|---|---|---|---|---|---|---|
| | | | Br⁻ | | 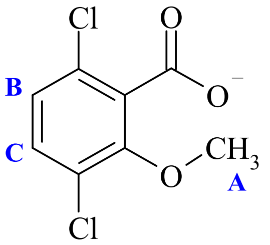  | |
| | | | δ (ppm) | *J* (Hz) | δ (ppm) | *J* (Hz) |
| **a** | 3H | triplet | 0.88 | 6.9 | 0.88 | 6.9 |
| **b** | 26H | multiplet | 1.26 | - | 1.26 | - |
| **c** | 2H | quintet | 1.73 | 7.3 | 1.64 | 8.0 |
| **d** | 2H | quintet | 1.82 | 5.1 | 1.72 | 6.3 |
| **e** | 4H | quintet | 1.93 | 5.1 | 1.84 | 6.0 |
| **f** | 3H | singlet | 3.34 | - | 3.27 | - |
| **g** | 2H | triplet | 3.65 | 8.5 | 3.51 | 8.6 |
| **h** | 4H | multiplet | 3.70, 3.78 | - | 3.57, 3.67 | - |
| **A** | 3H | singlet | - | - | 3.95 | - |
| **B** | 1H | doublet | - | - | 6.97 | 8.5 |
| **C** | 1H | doublet | - | - | 7.06 | 8.5 |

**Table S.11.** Chemical shift (δ) values in $^{13}$C NMR spectra for 1-hexadecyl-1-methylpiperidinium bromide and (3,6-dichloro-2-methoxy)benzoate ($CDCl_3$)

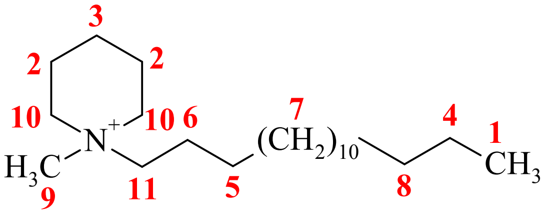

| carbon atoms | anion: $Br^-$ δ (ppm) | anion: 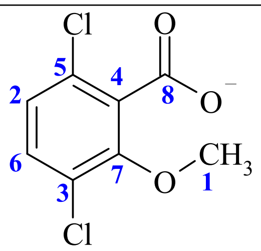 δ (ppm) |
|---|---|---|
| 1 | 13.7 | 13.9 |
| 2 | 19.9 | 19.9 |
| 3 | 20.3 | 20.5 |
| 4 | 21.7 | 21.8 |
| 5 | 22.3 | 22.4 |
| 6 | 26.0 | 26.1 |
| 7 | 28.90, 28.94, 29.02, 29.09, 29.20, 29.24, 29.28 | 29.07, 29.10, 29.18, 29.24, 29.35, 29.39, 29.43, 29.44 |
| 8 | 31.5 | 31.7 |
| 9 | 47.9 | 47.9 |
| 10 | 60.4 | 60.3 |
| 1 | - | 61.4 |
| 11 | 62.8 | 62.3 |
| 2 | - | 125.2 |
| 3 | - | 125.8 |
| 4 | - | 126.8 |
| 5 | - | 127.7 |
| 6 | - | 140.4 |
| 7 | - | 151.5 |
| 8 | - | 167.5 |

**Table S.12.** Chemical shifts (δ) and coupling constants (*J*) values in $^{1}$H NMR spectra for 1-methyl-1-octadecylpiperidinium bromide and (3,6-dichloro-2-methoxy)benzoate ($CDCl_3$)

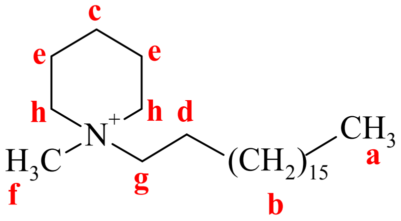


| protons | number of protons | multiplicity of the signal | anion: $Br^-$ | | anion: 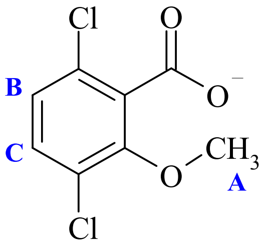 | |
|---|---|---|---|---|---|---|
| | | | δ (ppm) | *J* (Hz) | δ (ppm) | *J* (Hz) |
| **a** | 3H | triplet | 0.88 | 6.9 | 0.88 | 6.9 |
| **b** | 30H | multiplet | 1.26 | - | 1.26 | - |
| **c** | 2H | quintet | 1.73 | 7.5 | 1.64 | 7.5 |
| **d** | 2H | quintet | 1.82 | 5.5 | 1.73 | 6.4 |
| **e** | 4H | quintet | 1.93 | 5.1 | 1.85 | 5.4 |
| **f** | 3H | singlet | 3.34 | - | 3.26 | - |
| **g** | 2H | triplet | 3.65 | 8.5 | 3.50 | 8.6 |
| **h** | 4H | multiplet | 3.70, 3.78 | - | 3.56, 3.67 | - |
| **A** | 3H | singlet | - | - | 3.95 | - |
| **B** | 1H | doublet | - | - | 6.97 | 8.5 |
| **C** | 1H | doublet | - | - | 7.06 | 8.6 |

**Table S.13.** Chemical shift (δ) values in $^{13}C$ NMR spectra for 1-methyl-1-octadecylpiperidinium bromide and (3,6-dichloro-2-methoxy)benzoate ($CDCl_3$)

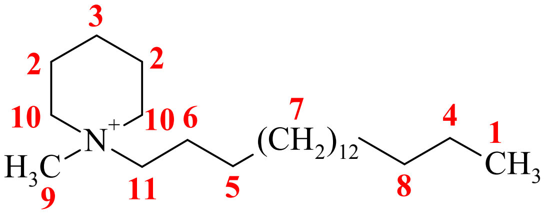


| carbon atoms | anion | |
|---|---|---|
| | $Br^-$ | 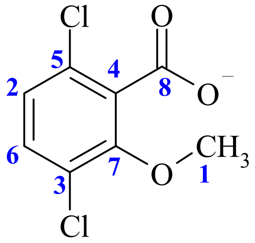 |
| | δ (ppm) | δ (ppm) |
| 1 | 13.8 | 13.9 |
| 2 | 19.9 | 19.9 |
| 3 | 20.3 | 20.5 |
| 4 | 21.7 | 21.8 |
| 5 | 22.3 | 22.4 |
| 6 | 26.1 | 26.1 |
| 7 | 28.95, 28.98, 29.07, 29.13, 29.24, 29.28, 29.33 | 29.07, 29.11, 29.19, 29.26, 29.37, 29.41, 29.46 |
| 8 | 31.5 | 31.7 |
| 9 | 48.0 | 48.0 |
| 10 | 60.5 | 60.4 |
| 1 | - | 61.5 |
| 11 | 62.8 | 62.4 |
| 2 | - | 125.2 |
| 3 | - | 125.8 |
| 4 | - | 126.9 |
| 5 | - | 127.7 |
| 6 | - | 140.2 |
| 7 | - | 151.5 |
| 8 | - | 167.6 |

## COLLECTED DATA

**Table S.14.** Thermal stability of obtained HILs ($T_{5\%}$ and $T_{50\%}$ denote the temperatures corresponding to 5% and 50% mass losses of the sample, respectively)

| $C_n$ | $T_{5\%}$ (°C) | $T_{50\%}$ (°C) |
|---|---|---|
| $C_8$ | 173.3 | 220.1 |
| $C_9$ | 173.8 | 222.7 |
| $C_{10}$ | 174.3 | 230.1 |
| $C_{12}$ | 180.1 | 230.5 |
| $C_{14}$ | 179.5 | 236.8 |
| $C_{16}$ | 178.0 | 257.4 |
| $C_{18}$ | 180.9 | 241.0 |

**Table S.15.** The static contact angles for the adaxial and abaxial sides of leaves and for paraffin

| contact angle (°) | | | | | |
|---|---|---|---|---|---|
| ADAXIAL SIDE | | | | | |
| $C_n$ | cornflower (*Centaurea cyanus* L.) | winter rapeseed (*Brassica napus* L.) | white mustard (*Sinapis alba* L.) | common wheat (*Triticum aestivum L.*) | paraffin |
| $C_8$ | 86.4 | 103.0 | 90.3 | 119.0 | 100.0 |
| $C_9$ | 85.7 | 100.0 | 89.7 | 118.3 | 100.2 |
| $C_{10}$ | 70.0 | 95.6 | 75.4 | 112.7 | 93.5 |
| $C_{12}$ | 57.4 | 88.9 | 63.2 | 99.2 | 69.6 |
| $C_{14}$ | 65.0 | 81.0 | 67.0 | 94.7 | 69.6 |
| $C_{16}$ | 61.0 | 79.0 | 64.8 | 92.5 | 73.3 |
| $C_{18}$ | 57.1 | 75.6 | 65.4 | 85.9 | 73.0 |
| water | 89.7 | 113.1 | 95.4 | 120.0 | 111.0 |
| dicash | 92.2 | 116.7 | 85.4 | 132.0 | 109.3 |

| contact angle (°) | | | | | |
|---|---|---|---|---|---|
| ABAXIAL SIDE | | | | | |
| $C_n$ | cornflower (*Centaurea cyanus* L.) | winter rapeseed (*Brassica napus* L.) | white mustard (*Sinapis alba* L.) | common wheat (*Triticum aestivum L.*) | paraffin |
| $C_8$ | 87.4 | 104.0 | 91.0 | 118.0 | 100.0 |
| $C_9$ | 86.7 | 101.0 | 88.1 | 116.0 | 100.2 |
| $C_{10}$ | 72.2 | 96.6 | 73.4 | 110.7 | 93.5 |
| $C_{12}$ | 56.4 | 88.2 | 64.9 | 99.0 | 69.6 |
| $C_{14}$ | 67.0 | 83.0 | 66.2 | 96.7 | 69.6 |
| $C_{16}$ | 62.3 | 77.1 | 62.8 | 93.5 | 73.3 |
| $C_{18}$ | 54.2 | 74.7 | 61.4 | 86.9 | 73.0 |
| water | 89.0 | 114.3 | 94.4 | 118.0 | 111.0 |
| dicash | 92.0 | 117.1 | 85.0 | 131.0 | 109.3 |

**Table S.16.** The sliding angles for the adaxial and abaxial sides of leaves

| contact angle (°) | | | | |
|---|---|---|---|---|
| ADAXIAL SIDE | | | | |
| $C_n$ | cornflower (*Centaurea cyanus* L.) | winter rapeseed (*Brassica napus* L.) | white mustard (*Sinapis alba* L.) | paraffin |
| $C_8$ | - | - | - | 17.3 |
| $C_9$ | - | - | - | 21.9 |
| $C_{10}$ | - | - | - | 25.6 |
| $C_{12}$ | 33.3 | 33.3 | 41.3 | 24.1 |
| $C_{14}$ | 27.9 | 43.2 | 37.7 | 16.2 |
| $C_{16}$ | 27.4 | 33.9 | 41.5 | 24.3 |
| $C_{18}$ | 37.8 | 43.4 | 36.4 | 24.0 |

| contact angle (°) | | | | |
|---|---|---|---|---|
| **ABAXIAL SIDE** | | | | |
| $C_n$ | cornflower (*Centaurea cyanus* L.) | winter rapeseed (*Brassica napus* L.) | white mustard (*Sinapis alba* L.) | paraffin |
| $C_8$ | - | - | - | 17.3 |
| $C_9$ | - | - | - | 21.9 |
| $C_{10}$ | - | - | - | 25.6 |
| $C_{12}$ | 34.3 | 35.3 | 39.3 | 24.1 |
| $C_{14}$ | 30.9 | 41.1 | 35.2 | 16.2 |
| $C_{16}$ | 25.4 | 30.9 | 44.1 | 24.3 |
| $C_{18}$ | 32.8 | 41.1 | 38.1 | 24.0 |

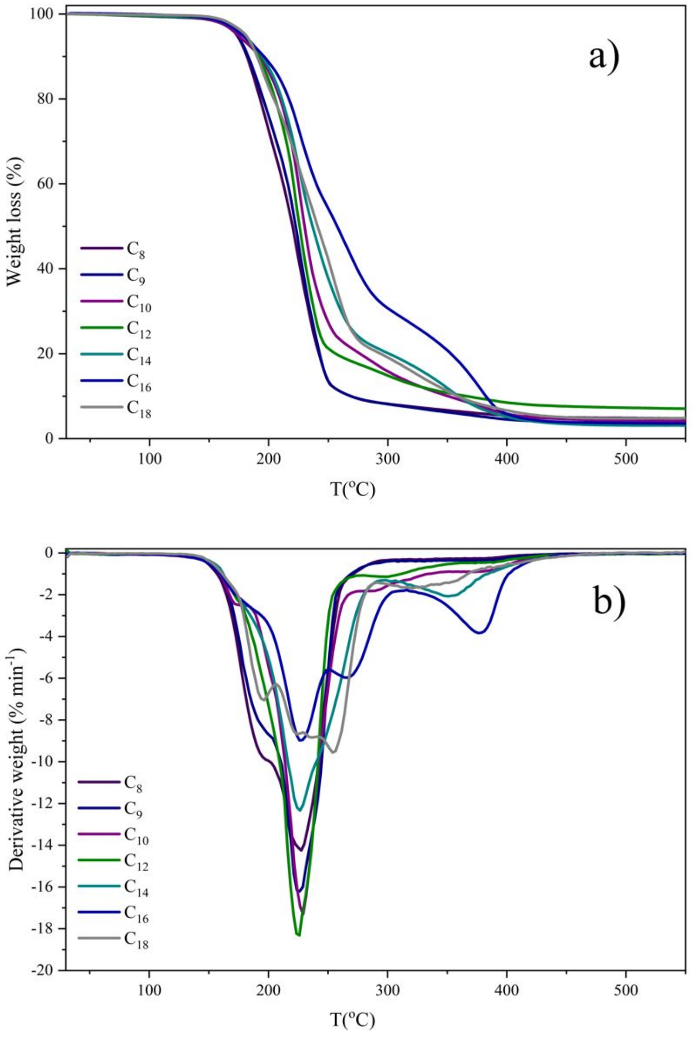


**Figure S.29.** a) TG and b) DTG curves of the investigated HILs. The number of carbon atoms in the alkyl chain of the substituent of the HIL cation are marked on the graphs.

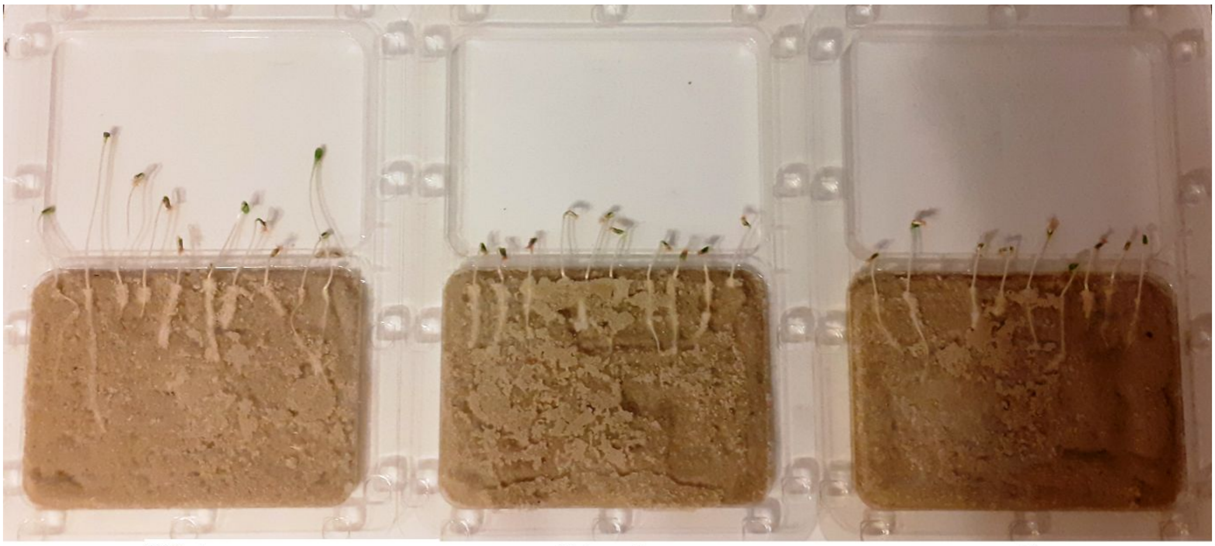

Water Dicash $C_8$

**Figure S.30.** The effect of 1-methyl-1-octylpiperidinium (3,6-dichloro-2-methoxy)benzoate and a commercial product – Dicash on cornflower (*Centaurea cyanus* L.) plants.

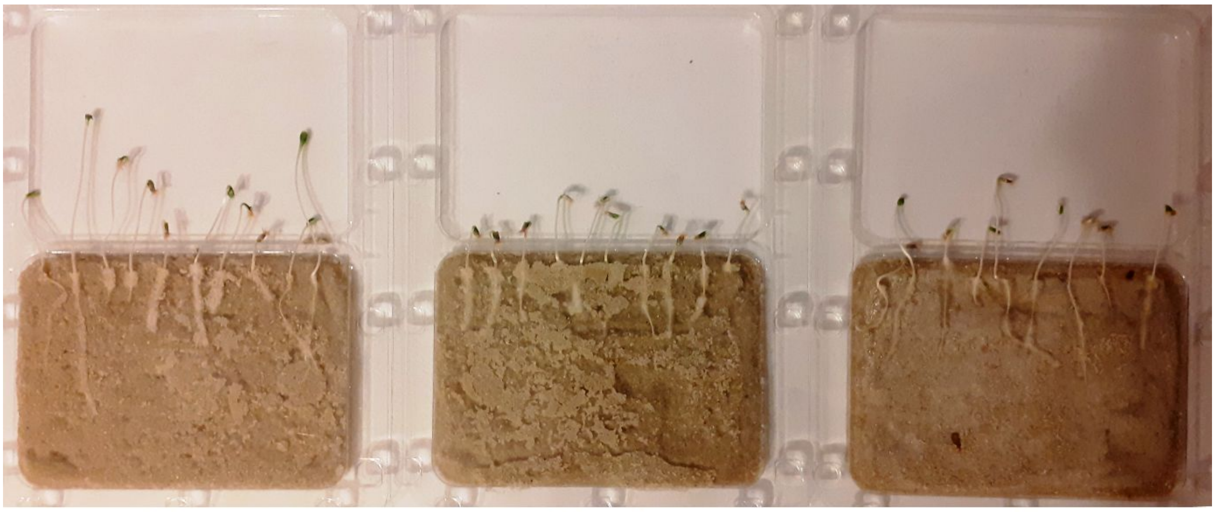

Water Dicash $C_9$

**Figure S.31.** The effect of 1-methyl-1-nonylpiperidinium (3,6-dichloro-2-methoxy)benzoate and a commercial product – Dicash on cornflower (*Centaurea cyanus* L.) plants.

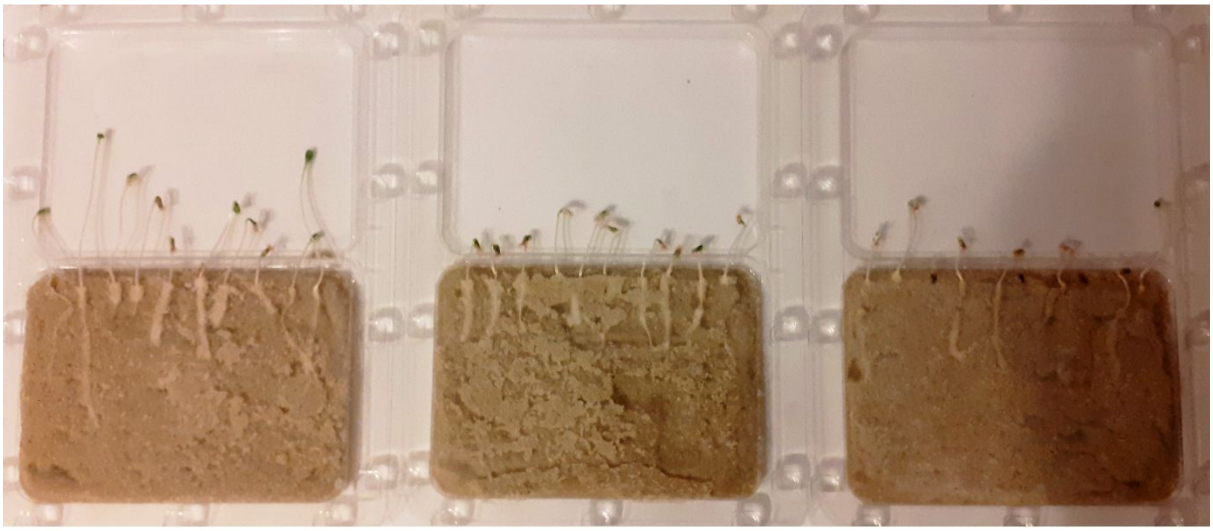

Water Dicash $C_{10}$

**Figure S.32.** The effect of 1-decyl-1-methylpiperidinium (3,6-dichloro-2-methoxy)benzoate and a commercial product – Dicash on cornflower (*Centaurea cyanus* L.) plants.

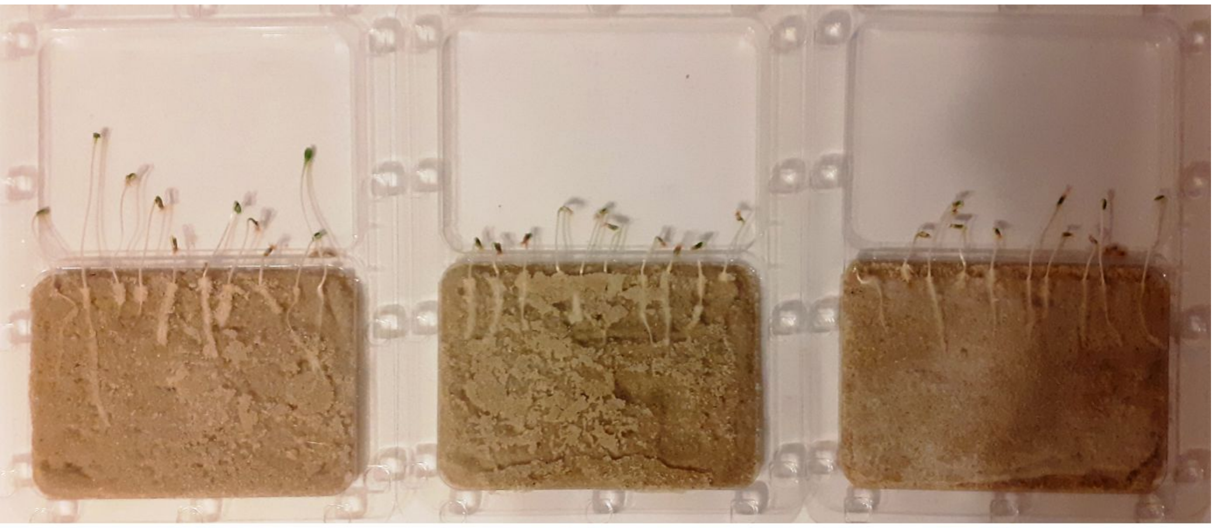

Water Dicash $C_{12}$

**Figure S.33.** The effect of 1-dodecyl-1-methylpiperidinium (3,6-dichloro-2-methoxy)benzoate and a commercial product – Dicash on cornflower (*Centaurea cyanus* L.) plants.

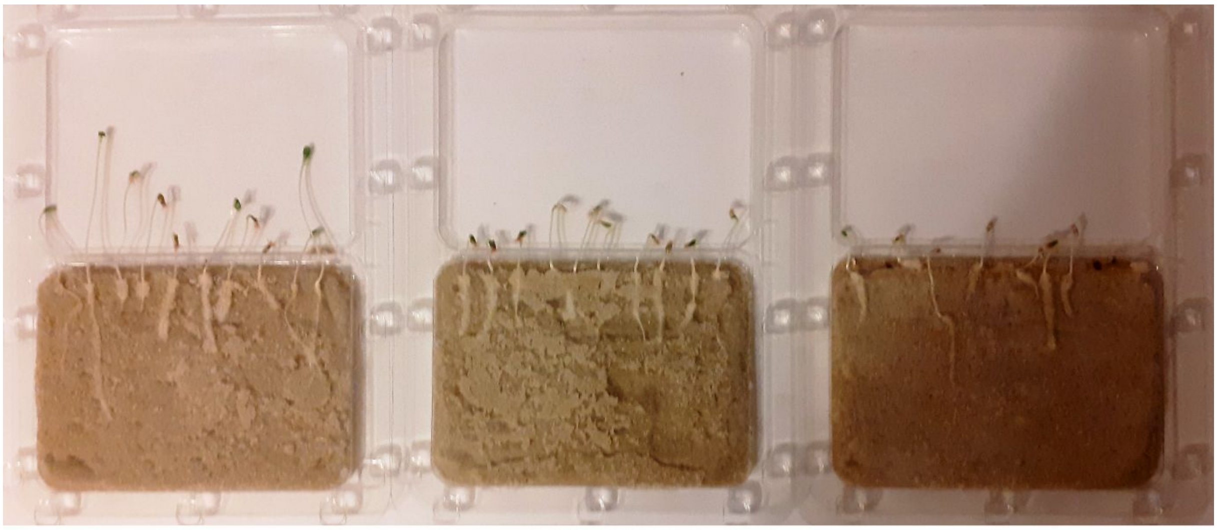

**Water** **Dicash** $C_{14}$

**Figure S.34.** The effect of 1-methyl-1-tetradecylpiperidinium (3,6-dichloro-2-methoxy)benzoate and a commercial product – Dicash on cornflower (*Centaurea cyanus* L.) plants.

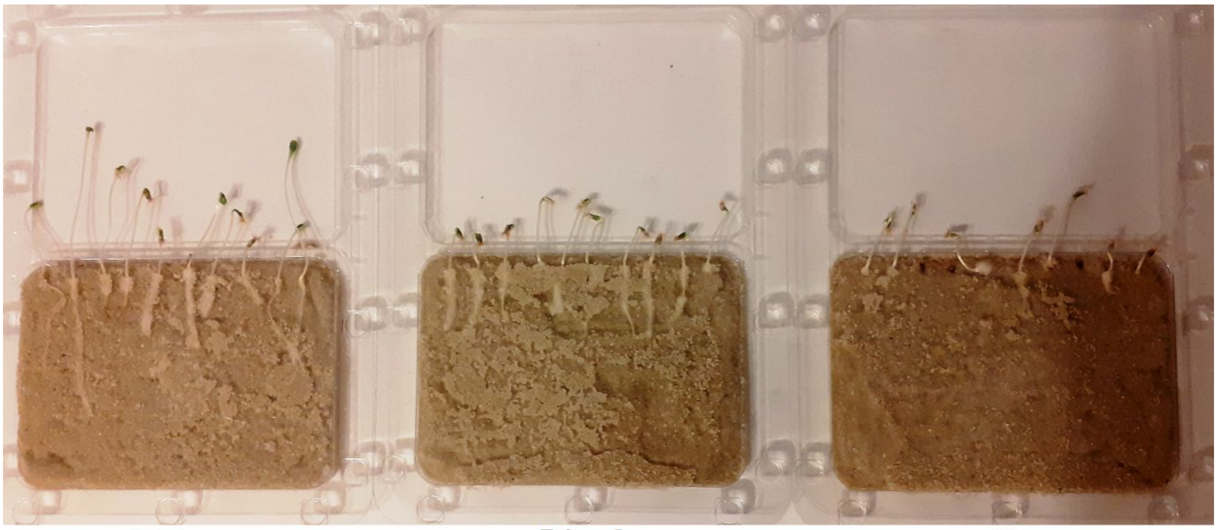

**Water** **Dicash** $C_{16}$

**Figure S.35.** The effect of 1-heksadecyl-1-methylpiperidinium (3,6-dichloro-2-methoxy)benzoate and a commercial product – Dicash on cornflower (*Centaurea cyanus* L.) plants.

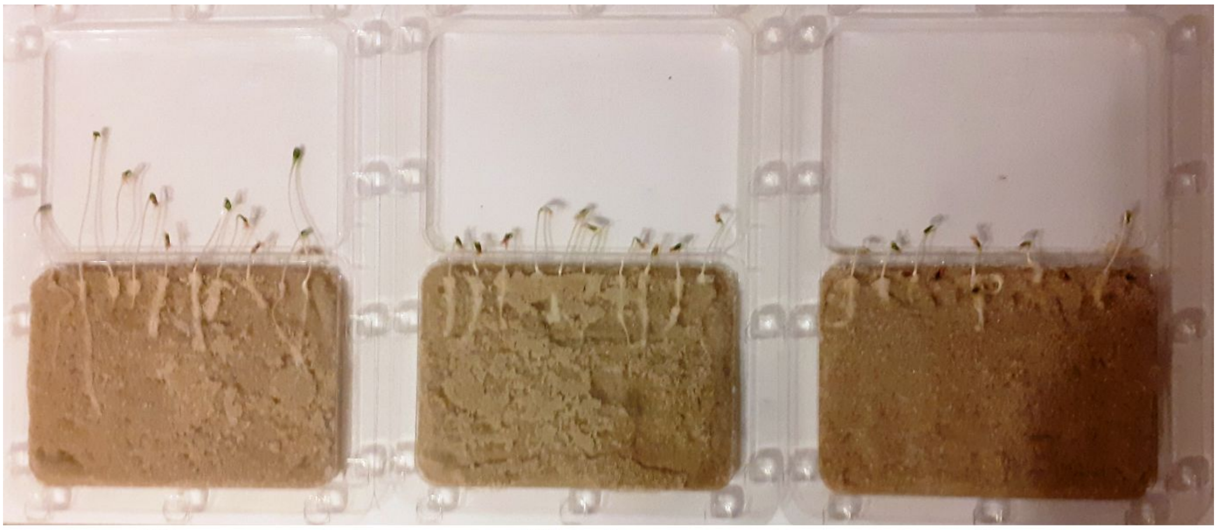

**Water** **Dicash** **$C_{18}$**

**Figure S.36.** The effect of 1-methyl-1-octadecylpiperidinium (3,6-dichloro-2-methoxy)benzoate and a commercial product – Dicash on cornflower (*Centaurea cyanus* L.) plants.
